# Routine versus deferred percutaneous coronary intervention in patients undergoing transcatheter aortic valve implantation: a frequentist and Bayesian meta-analysis of randomized trials with trial sequential analysis

**DOI:** 10.1016/j.ijcha.2026.102017

**Published:** 2026-09-22

**Authors:** Ahmed Ibrahim, Laila Shalabi, Domenico Mario Giamundo, Hadeel Aboueisha, Ahmed Enani, Mariam Assi, Ahmed Mojahed, Lucio Giuseppe Granata, Dan M. Meyer, Pierre Sabouret, Giuseppe Andò

**Affiliations:** a Faculty of Medicine, Alexandria University, Alexandria, Egypt; b Faculty of Medicine, Gharyan University, Gharyan, Libya; c Division of Cardiology, Policlinico Casilino, Rome, Italy; d Faculty of Medical Sciences, University of Groningen, University Medical Center Groningen, Groningen, The Netherlands; e College of Medicine and Health Sciences, UAE University, Al-Ain, United Arab Emirates; f Faculty of Medicine, Al Najah National University, Nablus, Palestine; g Cardiology Division, Garibaldi-Nesima Hospital, ARNAS Garibaldi, 95122 Catania, Italy; h Department of Cardiothoracic Surgery, Baylor Scott & White Health, Baylor University Medical Center, Dallas, TX, USA; i Heart Institute and Action Group, Pitié-Salpétrière, Sorbonne University, Paris, France; j Azienda Ospedaliera Papardo, Messina, Italy; k Department of Clinical and Experimental Medicine, University of Messina, Messina, Italy

**Keywords:** Coronary artery disease, Percutaneous coronary intervention, Randomized controlled trial, Routine PCI, Transcatheter aortic valve implantation

## Abstract

**Background:**

The optimal management of concomitant coronary artery disease (CAD) in patients undergoing transcatheter aortic valve implantation (TAVI) remains debated. Therefore, we performed this meta-analysis of randomized controlled trials (RCTs) to compare routine percutaneous coronary intervention (PCI) versus deferred PCI in patients with severe aortic stenosis and significant coronary artery disease undergoing TAVI.

**Methods:**

We systematically searched major electronic databases for eligible RCTs. The primary efficacy outcome was spontaneous myocardial infarction (MI); the primary safety outcome was major bleeding. Bayesian random-effects meta-analysis models were fitted using weakly informative prior distributions. Effect estimates are reported as posterior median risk ratios (RR) with 95% credible intervals (CrI). The posterior probability of benefit (PPB) for efficacy outcomes or harm (PPH) for safety outcomes was calculated (RR < 1 or RR > 1, respectively).

**Results:**

Three RCTs comprising 1156 patients were included. Routine PCI did not demonstrate a convincing posterior probability of benefit for spontaneous MI (posterior median RR 0.84, 95% CrI 0.43–1.73; PPB: 70.2%) or all-cause mortality (posterior median RR 0.91, 95% CrI 0.60–1.43; PPB: 69.6%). However, routine PCI was associated with a reduced risk of urgent revascularization (posterior median RR 0.37, 95% CrI 0.15–0.91; PPB: 98.1%). For safety outcomes, routine PCI was associated with an increased posterior probability of harm for major bleeding (posterior median RR 1.56, 95% CrI 0.91–2.71; PPH: 95.6%) and life-threatening or disabling bleeding (posterior median RR 1.74, 95% CrI 1.00–3.07; PPH: 97.4%).

**Conclusions:**

Among patients undergoing TAVI with concomitant CAD, routine PCI did not reduce ischemic events or mortality but increased bleeding complications. These findings suggest that a selective revascularization strategy, rather than a routine one, may be preferable.

**PROSPERO registration:**

CRD420261386909.

## Graphical abstract

**Figure:**
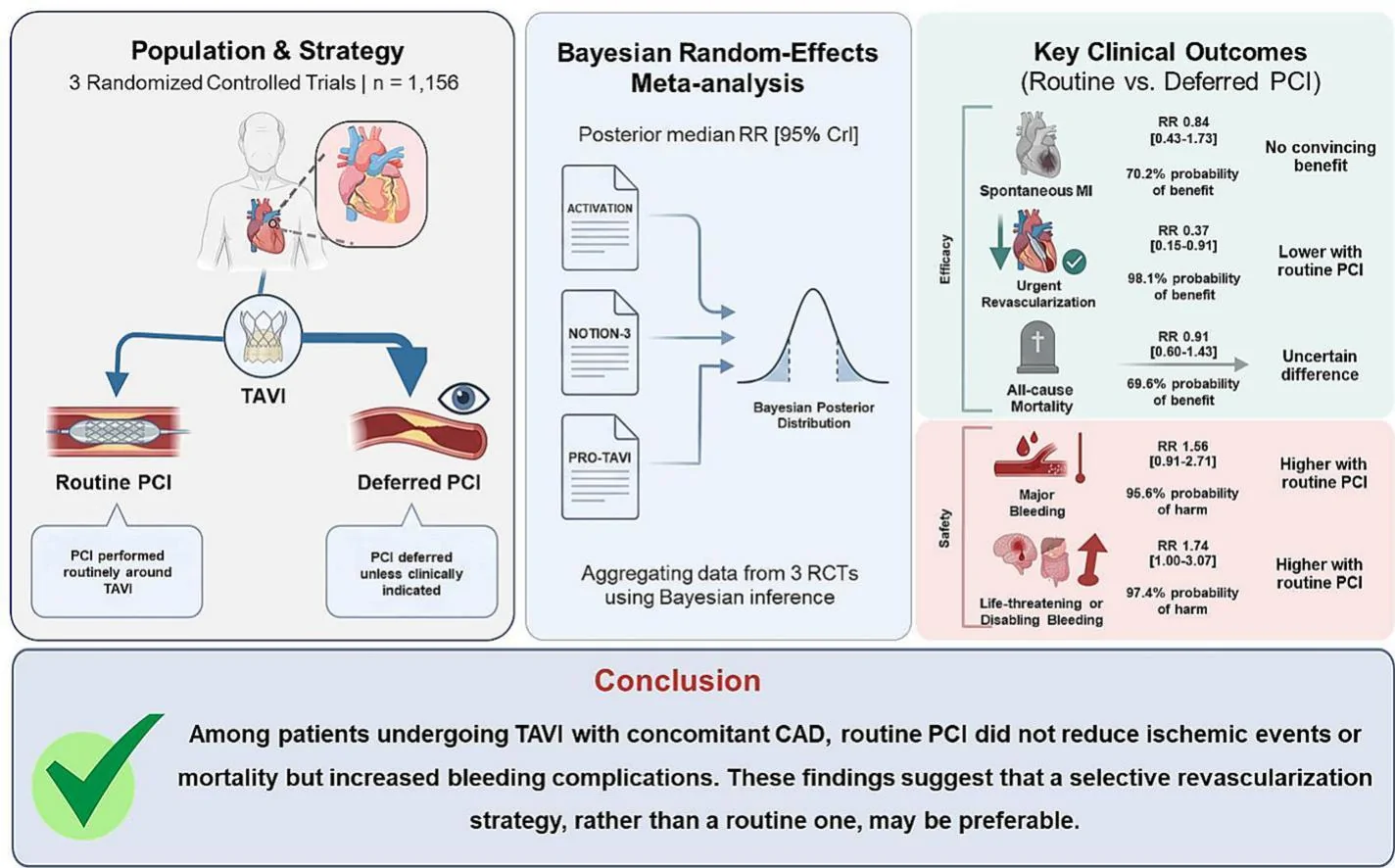

## 1 Introduction

Aortic stenosis represents the most prevalent valvular heart disease worldwide, with increasing incidence driven by population aging [1], [2]. The pathophysiology of aortic stenosis involves progressive leaflet thickening, fibrosis, and calcification. These processes are driven by inflammatory mechanisms that share striking similarities with atherosclerosis, including lipid accumulation, macrophage infiltration, and tissue calcification [2]. This pathophysiological overlap explains the high prevalence of concomitant coronary artery disease (CAD) in patients with severe aortic stenosis, affecting approximately 40–75% of patients undergoing transcatheter aortic valve implantation (TAVI) [1], [3], [4]. The coexistence of these conditions creates complex hemodynamic challenges, as aortic stenosis induces left ventricular hypertrophy, increased diastolic filling pressures, and coronary microvascular dysfunction that can exacerbate myocardial ischemia even in the absence of epicardial CAD [5].

The clinical impact of untreated CAD in TAVI recipients remains controversial. While some studies demonstrate increased rates of myocardial infarction (MI) and unplanned revascularization in patients with CAD, the association with all-cause mortality and major adverse cardiovascular events (MACE) has been inconsistent [6], [7]. Severe CAD appears to confer worse long-term outcomes, though this effect may reflect overall comorbidity burden rather than an independent prognostic factor.

Percutaneous coronary intervention (PCI) aims to restore coronary blood flow by relieving hemodynamically significant stenoses, theoretically reducing ischemic events and improving outcomes after TAVI [8], [9]. The rationale for routine PCI includes preventing periprocedural MI, reducing future coronary events, and ensuring coronary access before valve deployment, which potentially complicates future interventions [9]. However, PCI carries inherent risks, including bleeding complications from dual antiplatelet therapy, procedural complications, and contrast-induced acute kidney injury, particularly in this elderly, high-risk population [8], [10].

Clinical guideline recommendations reflect this clinical dilemma. The 2025 European Society of Cardiology/European Association for Cardio-Thoracic Surgery (ESC/EACTS) guidelines on valvular heart disease, informed by the NOTION-3 trial findings [1], recommend PCI before TAVI primarily for critical anatomical lesions (> 90% diameter stenosis or left main disease; Class IIa, Level B), while adopting a more selective approach for intermediate lesions (70–90% diameter stenosis; Class IIb, Level B) [11]. However, the supporting evidence is still conflicting. The NOTION-3 trial demonstrated that routine PCI reduced the composite endpoint of death, MI, or urgent revascularization compared with conservative management, although this was associated with increased bleeding events [1]. In contrast, the PRO-TAVI trial demonstrated that deferral of PCI was non-inferior to routine PCI before TAVI for the composite of all-cause mortality, MI, stroke, and major bleeding at 1 year [12].

Given these discordant findings, the balance between ischemic efficacy and procedural safety remains unclear. Therefore, we conducted a systematic review and meta-analysis of all available randomized controlled trials (RCTs), employing both frequentist and Bayesian frameworks to compare the efficacy and safety of a routine PCI strategy versus a deferred PCI strategy in patients with severe aortic stenosis and concomitant CAD undergoing TAVI.

## 2 Methods

### 2.1 Study design and registration

This systematic review and meta-analysis was conducted in accordance with the methodological framework established by the Cochrane Handbook for Systematic Reviews of Interventions [13] and reported in compliance with the Preferred Reporting Items for Systematic Reviews and Meta-Analyses (PRISMA) 2020 statement [14]. The study protocol was registered prospectively in the International Prospective Register of Systematic Reviews (PROSPERO; registration identifier: CRD420261386909).

### 2.2 Search strategy

A comprehensive systematic literature search was executed in PubMed, Embase, Scopus, and Web of Science from database inception through April 4, 2026, without language restrictions. The search strategy combined Medical Subject Headings and free-text terms for (“transcatheter aortic valve implantation” OR “TAVI” OR “TAVR” OR “transcatheter aortic valve replacement”) AND (“percutaneous coronary intervention” OR “PCI” OR “coronary revascularization” OR “stenting”) AND (“randomized controlled trial” OR “RCT” OR “randomized”). The detailed search strategies for each database are presented in Table S1.

### 2.3 Eligibility criteria

We included RCTs that enrolled adult patients with severe symptomatic aortic stenosis and concomitant significant CAD, as defined by each trial (Table S2), who were undergoing TAVI and that compared a strategy of routine planned PCI (performed before, during, or shortly after TAVI) versus a strategy of no planned PCI, defined as conservative management or deferral of PCI until clinically indicated. We excluded observational studies, including cohort studies, case-control studies, case series, and case reports, as well as review articles, editorials, commentaries, and study protocols without results.

### 2.4 Endpoints

The primary efficacy endpoint was spontaneous MI. The primary safety endpoint was major bleeding. Secondary endpoints were all-cause mortality, cardiovascular death, any MI (spontaneous and procedural), stroke (any type), any revascularization, urgent revascularization, any bleeding, minor bleeding, life-threatening or disabling bleeding, acute kidney injury (AKI), and trial-defined MACE. Functional outcomes were also assessed using the New York Heart Association (NYHA) functional class (I-IV) at 12 months after TAVI and the Canadian Cardiovascular Society (CCS) angina class (0-IV), with higher classes indicating greater symptom severity. Detailed endpoint definitions for each included trial are presented in Table S3.

### 2.5 Study selection and data extraction

Duplicate records were removed using Mendeley Reference Manager, and the remaining records were screened using Rayyan systematic review software. Two reviewers (A.E and M.A) independently screened titles and abstracts, followed by full-text assessment against the predefined eligibility criteria. Disagreements were resolved through discussion with a third author (L.S). Reference lists of included studies and relevant review articles were hand-searched to identify additional eligible trials not captured by the electronic database search.

Two independent reviewers (A.E and A.M) extracted data using a standardized data collection sheet. Extracted information included trial characteristics (first author, year of publication, trial name, number of centers, countries, enrollment period, sample size, and definition of significant coronary artery disease), patient characteristics, procedural characteristics, and outcome events (number of events and total patients in each arm for all outcomes of interest). All data were subsequently revised by a third author (H.A) for accuracy.

### 2.6 Risk of Bias and certainty assessment

Two reviewers (M.A. and A.M.) independently evaluated the risk of bias for all included studies using the Cochrane Risk of Bias 2 (RoB-2) tool [15]. Assessment covered five domains: the randomization process, deviations from the intended interventions, incomplete outcome data, measurement of outcomes, and bias in selection of the reported results. For each outcome, the certainty of evidence was appraised independently by the same reviewers using the GRADE approach. Evidence from randomized trials was initially rated as high and could be downgraded based on risk of bias, inconsistency, indirectness, imprecision, and publication bias. Certainty was classified as high, moderate, low, or very low. Disagreements were resolved through discussion until consensus was reached [16].

### 2.7 Statistical analysis

All statistical analyses were performed using R version 4.4.2 (R Foundation for Statistical Computing) with the `meta` and `bayesmeta` packages. Trial Sequential Analysis (TSA) was performed using TSA software version 0.9.5.10 Beta (Copenhagen Trial Unit).

#### 2.7.1 Bayesian analysis

Primary analyses were performed under a Bayesian random-effects meta-analytic framework to better characterize parameter uncertainty given the small number of included trials. Weakly informative priors were specified: a normal prior for the log risk ratio (RR) (mean = 0, SD = 2) for the pooled effect parameter (μ), and a half-normal prior for the between-study heterogeneity standard deviation (τ) with scale = 0.5 [17]. Pooled effect sizes are reported as posterior median RRs with 95% credible intervals (CrIs). For efficacy endpoints, we calculated the posterior probability of benefit (RR < 1) and clinically meaningful benefit (RR < 0.8). For safety endpoints, we calculated the posterior probability of harm (RR > 1) and clinically meaningful harm (RR > 1.25). Under the Bayesian framework, effect estimates are partially pooled toward the overall mean, a phenomenon known as shrinkage, which reduces extreme estimates from small or imprecise studies, thereby producing more stable and clinically plausible effect sizes compared to conventional meta-analytic estimators. To assess the robustness of our findings to the prior specification for τ, we conducted a sensitivity analysis using alternative heterogeneity priors: a half-normal distribution with scale = 1.0, a half-Cauchy distribution with scale = 0.5, and a half-Cauchy distribution with scale = 1.0, all compared against the primary half-normal (scale = 0.5) prior. Posterior estimates from all sensitivity models were compared to evaluate the influence of the heterogeneity prior on the pooled effect size and CrIs.

#### 2.7.2 Frequentist analysis

Secondary frequentist meta-analyses were performed under a random-effects framework using the restricted maximum likelihood (REML) estimator to quantify between-study variance (τ^2^). This approach was selected over the DerSimonian-Laird method due to its reduced bias and superior coverage properties when the number of included studies is small [18]. Given that conventional random-effects models may yield overly narrow confidence intervals with few included trials, conventional 95% CIs were complemented with 95% CIs calculated using the Hartung-Knapp-Sidik-Jonkman [HKSJ] adjustment as a sensitivity analysis [19], [20]. Pooled effect sizes were expressed as RRs, calculated via the inverse-variance weighting method. In instances of zero events in both intervention arms, a standard continuity correction of 0.5 was applied to all cells of the affected contingency table to permit variance estimation. Between-study heterogeneity was assessed using the I^2^ statistic. According to the Cochrane Handbook for Systematic Reviews of Interventions, I^2^ values of 0%–40% may represent unimportant heterogeneity, 30%–60% moderate heterogeneity, 50%–90% substantial heterogeneity, and 75%–100% considerable heterogeneity [13]. For I^2^ values that fell within overlapping ranges, both applicable descriptors were reported. Formal assessment of small-study effects, including Egger's regression test and visual inspection of funnel plot asymmetry, was precluded due to the inclusion of only three trials, as such diagnostics are unreliable and lack sufficient statistical power when fewer than ten studies are available [21]. A two-sided *p* < 0.05 threshold defined statistical significance.

#### 2.7.3 Sensitivity analysis

We also performed sensitivity analyses to assess the robustness of the MACE estimate. First, we excluded the NOTION-3 trial because its definition of MACE included urgent revascularization as a component of the composite. Revascularization is a “softer” endpoint that can be influenced by ascertainment and treatment bias, whereas the other trials defined MACE using harder clinical endpoints. Second, we restricted the analysis to 1-year follow-up to harmonize follow-up duration across trials. For this analysis, we used hazard ratios (HRs) as reported in the ACTIVATION and PRO-TAVI trials [12], [22]. For NOTION-3, which did not report 1-year HR [1], we reconstructed time-to-event data from the published Kaplan-Meier curves using the method described by Li et al. [23] to estimate the 1-year HR for MACE.

#### 2.7.4 Trial sequential analysis

Trial sequential analysis (TSA) was performed to adjust for the risk of random error due to sparse data and repetitive testing in cumulative meta-analyses for spontaneous MI, bleeding outcomes (any bleeding, major bleeding, and life-threatening bleeding), and revascularization outcomes (any revascularization and urgent revascularization) [24], [25]. The required information size (RIS) was calculated assuming a 5% (two-sided) type I error rate, 80% power, the observed event proportion in the control arm, and the anticipated relative risk reduction from the pooled estimate. To account for between-study heterogeneity in the required information size calculation, a diversity-adjusted model was used, which incorporates the D^2^ statistic to inflate the required information size when heterogeneity is present. Trial sequential monitoring boundaries were constructed. For outcomes where the cumulative *Z*-curve crossed the trial sequential monitoring boundary for benefit or harm, the finding was considered conclusive regardless of whether the diversity-adjusted required information size (DARIS) was reached. For outcomes where the Z-curve did not cross the boundary, and DARIS was not reached, the evidence was deemed inconclusive and potentially subject to type II error.

## 3 Results

### 3.1 Study selection and characteristics

The systematic literature search identified 1199 records. After removal of duplicates, 871 unique records remained. Title and abstract screening yielded 23 articles for full-text assessment, of which 3 RCTs met the inclusion criteria [1], [12], [22] (Fig. 1).

**Fig. 1:**
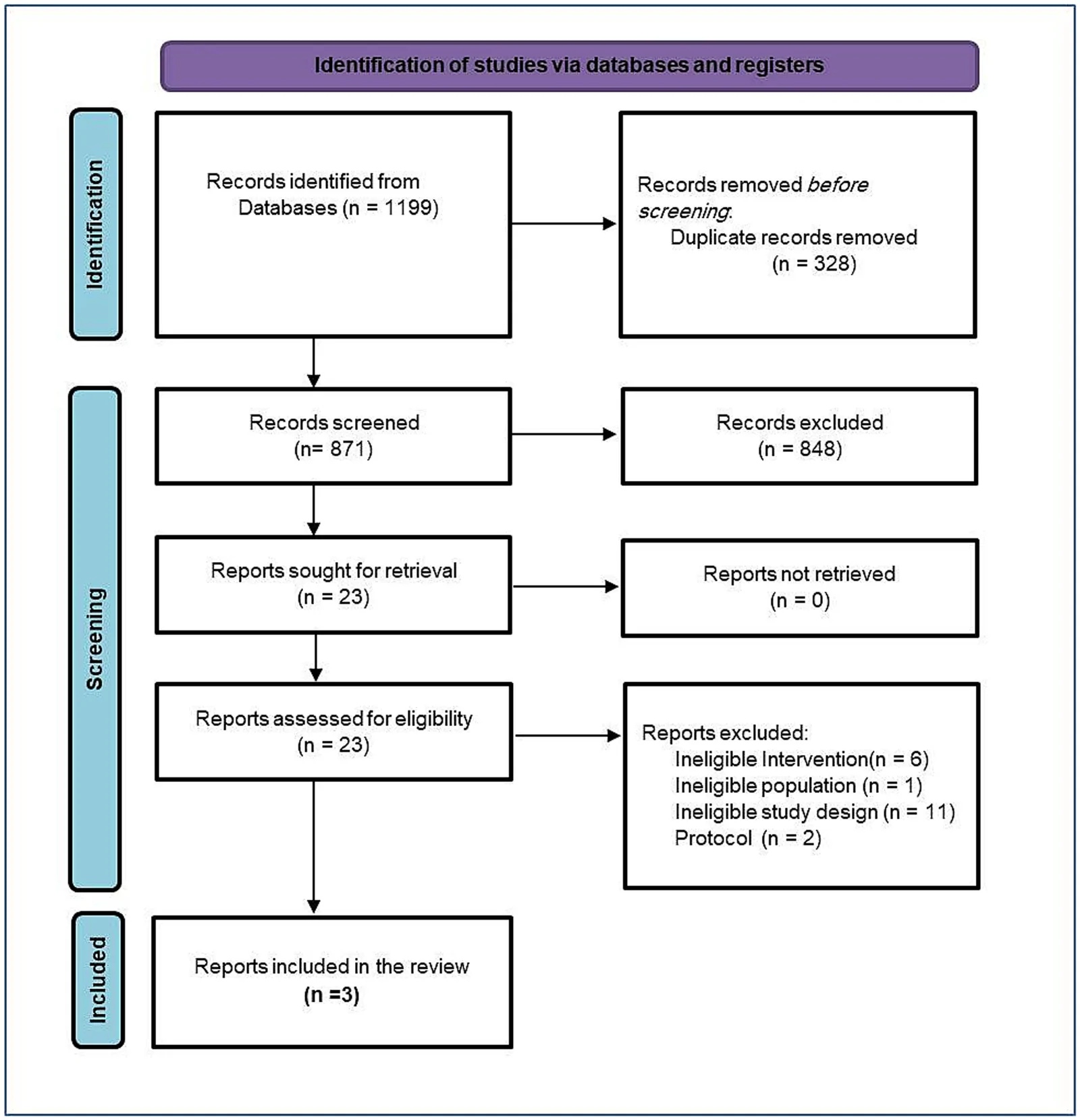
PRISMA 2020 Flow Diagram of Study Selection.

A total of 1156 patients were included across 3 RCTs. Of these, 579 patients (50.1%) underwent TAVI with preceding PCI, whereas 577 patients (49.9%) with significant CAD underwent TAVI only.

Baseline characteristics are summarized in Table 1, Table 2, and S4. The pooled cohort was elderly (median age 81–84 years) and predominantly male (64%). Most patients (90%) underwent transfemoral TAVI, with balloon-expandable device use ranging from 40% to 93% across trials. Surgical risk varied substantially between trials: the ACTIVATION trial enrolled a higher-risk cohort (median EuroSCORE 11.3–13.9%), whereas NOTION-3 and PRO-TAVI enrolled low-to-intermediate risk patients (median STS-PROM 3.0–3.1%) [1], [12]. CAD complexity was low overall, with a median SYNTAX score of 9–10 in NOTION-3 and PRO-TAVI; however, SYNTAX scores were not reported in the ACTIVATION trial [22]. Cardiovascular risk factors were highly prevalent, including hypertension (74%), atrial fibrillation (34%), and diabetes mellitus (28%). The use of oral anticoagulation was documented in 32% of patients.

**Table 1:** Baseline Characteristics of Included Randomized Controlled Trials.

Trial	Registry number	Country	No. of Centers	Blinding status	Randomization Method	Recruitment period	PCI	No PCI	No. of participants	Follow-up	Definition of Significant CAD	Main Inclusion Criteria	Main Exclusion Criteria	Primary Outcome
ACTIVATION	ISRCTN75836930	UK, France, Germany	17	Open-label	Electronic system, permuted block method	Dec 2012 – Jan 2019	119	116	235	1 year	Stenosis ≥70% (≥50% left main/vein graft)	Severe AS suitable for TAVR; significant CAD	ACS <30d; CCS class ≥III; unprotected left main; DAPT prohibition	Composite of all-cause mortality and rehospitalization
NOTION-3	NCT03058627	Denmark, Finland, Sweden	12	Open-label	Permuted blocks, stratified by center	Sep 2017 – Oct 2022	227	228	455	Median 2 years	FFR ≤0.80 or diameter stenosis ≥90%	Severe AS and significant coronary stenosis	Life expectancy <1y; ACS <14d; left main stenosis	Composite of death, MI, or urgent revascularization
PRO-TAVI	NCT05078619	Netherlands	12	Open-label	Web-based system (Castor), random blocks	Oct 2021 – Nov 2024	233	233	466	1 year	Stenosis 70–99% or 40–70% with positive physiology	Indication for TAVI and clinically significant CAD	Unprotected left-main CAD; solitary chronic total occlusion	Composite of death, MI, stroke, or major bleeding

**Abbreviations:** AS: Aortic Stenosis; ACS: Acute Coronary Syndrome; CAD: Coronary Artery Disease; CCS: Canadian Cardiovascular Society; DAPT: Dual Antiplatelet Therapy; FFR: Fractional Flow Reserve; MI: Myocardial Infarction; PCI: Percutaneous Coronary Intervention; TAVI/TAVR: Transcatheter Aortic Valve Implantation/Replacement.

**Table 2:** Baseline Clinical Characteristics of the Included Patients.

Study ID	Age, mean (SD) or median (IQR), y	Male, No. (%)	BMI (kg/m^2^)	NYHA Class III/IV, No. (%)	Angina (CCS class I–IV), No. (%)	Prior Myocardial Infarction, No. (%)	Prior PCI, No. (%)	Prior CABG, No. (%)	Atrial Fibrillation, No. (%)	Hypertension, No. (%)	Diabetes Mellitus, No. (%)	Hypercholesterolemia, No. (%)	Current or Former Smoking, No. (%)	Peripheral Vascular Disease, No. (%)	Surgical Risk Score (STS-PROM/EuroSCORE^#^) % (median, IQR)	SYNTAX Score (median, IQR)
**ACTIVATION**																
PCI (*n* = 119)	83.6 ± 5.0	69 (58.0)	28.7 ± 6.1	71 (61.2)	38 (31.9)	14 (11.8)	28 (23.5)	8 (6.7)	–	94 (79.0)	35 (29.4)	40 (33.6)	6 (5.0)	15 (12.6)	11.3 (1.4–63.8)* #	–
No PCI (*n* = 116)	84.3 ± 5.0	75 (64.7)	26.6 ± 5.0	65 (58.1)	35 (30.7)	26 (22.8)	24 (21.1)	11 (9.6)	–	90 (77.6)	27 (23.3)	34 (29.3)	3 (2.6)	24 (21.1)	13.9 (1.2–77.4)*#	–
**NOTION-3**																
PCI (*n* = 227)	82 (78–85)*	154 (68)	26 (24–29)*	105 (46)	125 (55)	14 (6)	28 (12)	7 (3)	81 (36)	158 (70)	59 (26)	158 (70)	113 (50)	19 (8)	3 (2–4)*	9 (6–14)*
No PCI (*n* = 228)	81 (78–85)*	153 (67)	26 (24–29)*	95 (42)	128 (56)	24 (11)	37 (16)	10 (4)	74 (32)	168 (74)	61 (27)	169 (74)	133 (58)	26 (11)	3 (2–4)*	9 (5–14)*
**PRO-TAVI**																
PCI (*n* = 233)	81 (78–84)*	151 (65)	26.0 (23.8–29.3)*	116 (50)	103 (44)	57 (24)	51 (22)	19 (8)	82 (35)	157 (67)	75 (32)	151 (65)	–	40 (17)	3.1 (2.0–5.2)*	10 (5–17)*
No PCI (n = 233)	81 (77–84)*	149 (64)	25.9 (23.6–29.0)*	123 (53)	85 (36)	46 (20)	47 (20)	13 (6)	80 (34)	154 (66)	75 (32)	148 (64)	–	41 (18)	3.1 (1.9–4.9)*	10 (6–17)*

**Abbreviations:** BMI, body mass index; CABG, coronary artery bypass grafting; CCS, Canadian Cardiovascular Society; IQR, interquartile range; NYHA, New York Heart Association; PCI, percutaneous coronary intervention; SD, standard deviation; STS-PROM, Society of Thoracic Surgeons Predicted Risk of Mortality; SYNTAX, Synergy between Percutaneous Coronary Intervention with Taxus and Cardiac Surgery. *Data presented as median and interquartile range. # Surgical risk score is reported as logistic EuroSCORE (%) for the ACTIVATION trial, and as STS-PROM score (%) for the NOTION-3 and PRO-TAVI trials.

### 3.2 Risk of Bias assessment

All three trials were assessed as having a low risk of bias using the Cochrane Risk of Bias 2 tool [1], [12], [22]. Although the trials were open-label, independent clinical events committees blinded to treatment allocation adjudicated all hard endpoints, which minimize detection bias (Fig. S1).

### 3.3 Primary endpoints

The primary and secondary endpoint results are summarized in Fig. 2, Fig. 3, Fig. 4, Fig. 5 and S2–S57.

**Fig. 2:**
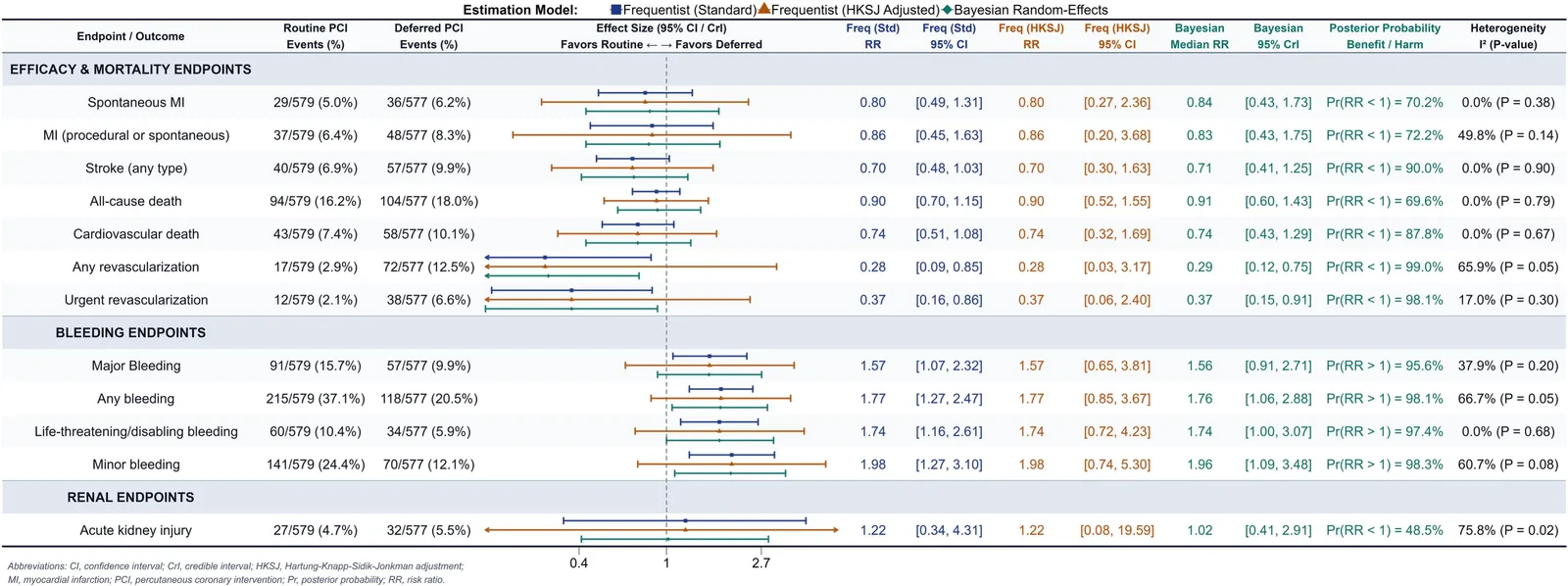
Summary of Primary and Secondary Outcomes: Frequentist and Bayesian Meta-Analyses. For each endpoint, event counts and total patients are shown for both treatment groups (Routine PCI vs. Deferred PCI). The frequentist random-effects model reports the pooled risk ratio with 95% confidence interval and the I^2^ heterogeneity statistic. The Bayesian model reports the posterior median risk ratio with 95% credible interval and the posterior probability of benefit (RR <1) or harm (RR >1).

**Fig. 3:**
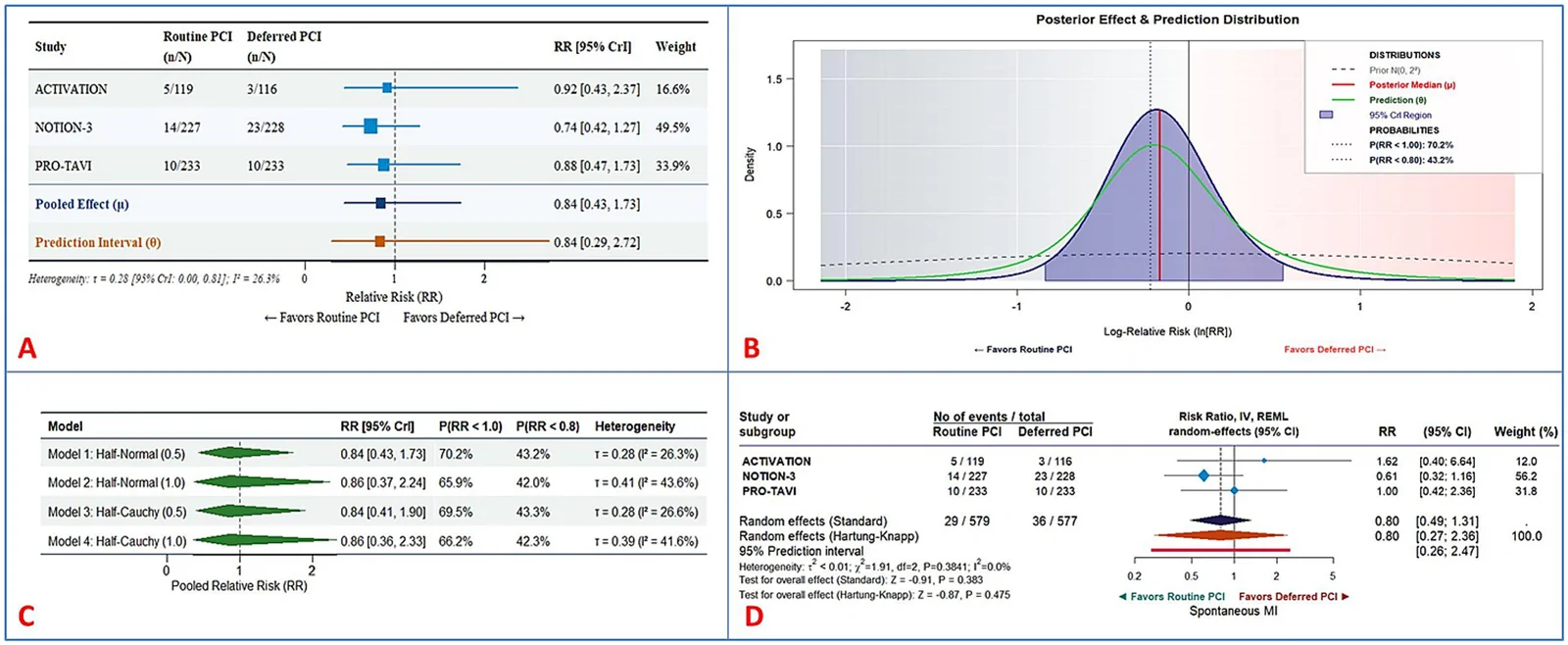
Primary Efficacy Endpoint: Spontaneous Myocardial Infarction (MI) (A) Bayesian Forest Plot (Shrinkage Estimates). Forest plot showing trial-level posterior shrinkage estimates for spontaneous MI comparing Routine percutaneous coronary intervention (PCI) vs. Deferred PCI. Each trial is represented by a point estimate with 95% credible interval (CrI). The pooled posterior median RR and 95% CrI are presented at the bottom. (B) Bayesian Posterior Distribution. Density plot of log-relative risk [ln(RR)] for spontaneous MI. The plot displays three distributions: the prior distribution [dashed gray line, N(0,2^2^)], the posterior distribution of the pooled effect [solid blue line], and the predictive distribution for a future trial [solid orange line]. The shaded blue area indicates the 95% CrI region. The x-axis is annotated to indicate the direction of effect favoring Routine PCI and Deferred PCI. A legend box reports the prior specification and posterior probabilities for P(RR < 1.0) and P(RR < 0.80). (C) Bayesian Meta-Analysis of Pooled (RR) Using Different Prior Distributions for Between-Study Heterogeneity. Forest plot showing pooled RR estimates from four Bayesian random-effects models with varying priors for the between-study heterogeneity parameter τ. For each model, the posterior median RR is shown with 95% CrI. Posterior probabilities for P(RR < 1.0) and P(RR < 0.80) are reported. Heterogeneity is quantified by τ and I^2^. The vertical line at RR = 1.0 indicates no difference between groups. (D) Frequentist Forest Plot. Forest plot displaying the RR for spontaneous MI for each individual trial with 95% confidence intervals (CIs) [horizontal lines]. The size of each square is proportional to the trial weight in the pooled analysis. The diamond represents the pooled estimate from the random-effects model, with the width of the diamond indicating the 95% CI.

**Fig. 4:**
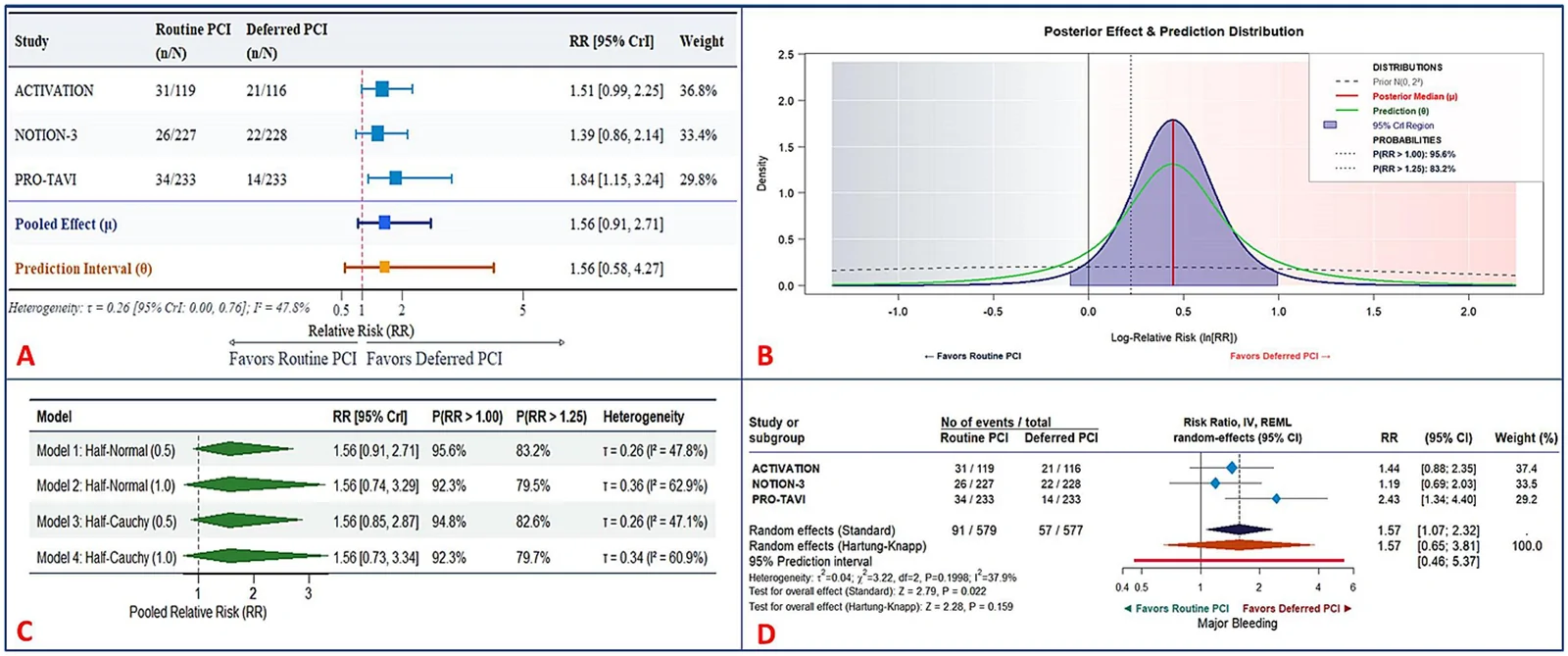
Primary Safety Endpoint: Major Bleeding (A) Bayesian Forest Plot (Shrinkage Estimates). Forest plot showing trial-level posterior shrinkage estimates for major bleeding comparing Routine percutaneous coronary intervention (PCI) vs. Deferred PCI. Each trial is represented by a point estimate with 95% credible interval (CrI). The pooled posterior median RR and 95% CrI are presented at the bottom. (B) Bayesian Posterior Distribution. Density plot of log-relative risk [ln(RR)] for major bleeding. The plot displays three distributions: the prior distribution [dashed gray line, N(0, 2^2^)], the posterior distribution of the pooled effect [solid blue line], and the predictive distribution for a future trial [solid orange line]. The shaded blue area indicates the 95% CrI region. The x-axis is annotated to indicate the direction of effect favoring Routine PCI and Deferred PCI. A legend box reports the prior specification and posterior probabilities for P(RR > 1.0) and P(RR > 1.25). (C) Bayesian Meta-Analysis of Pooled RR Using Different Prior Distributions for Between-Study Heterogeneity. Forest plot showing pooled RR estimates from four Bayesian random-effects models with varying priors for the between-study heterogeneity parameter τ. For each model, the posterior median RR is shown with 95% CrI. Posterior probabilities for P(RR > 1.0) and P(RR > 1.25) are reported. Heterogeneity is quantified by τ and I^2^. The vertical line at RR = 1.0 indicates no difference between groups. (D) Frequentist Forest Plot. Forest plot displaying the RR for major bleeding for each individual trial with 95% confidence intervals (CIs) [horizontal lines]. The size of each square is proportional to the trial weight in the pooled analysis. The diamond represents the pooled estimate from the random-effects model, with the width of the diamond indicating the 95% CI.

**Fig. 5:**
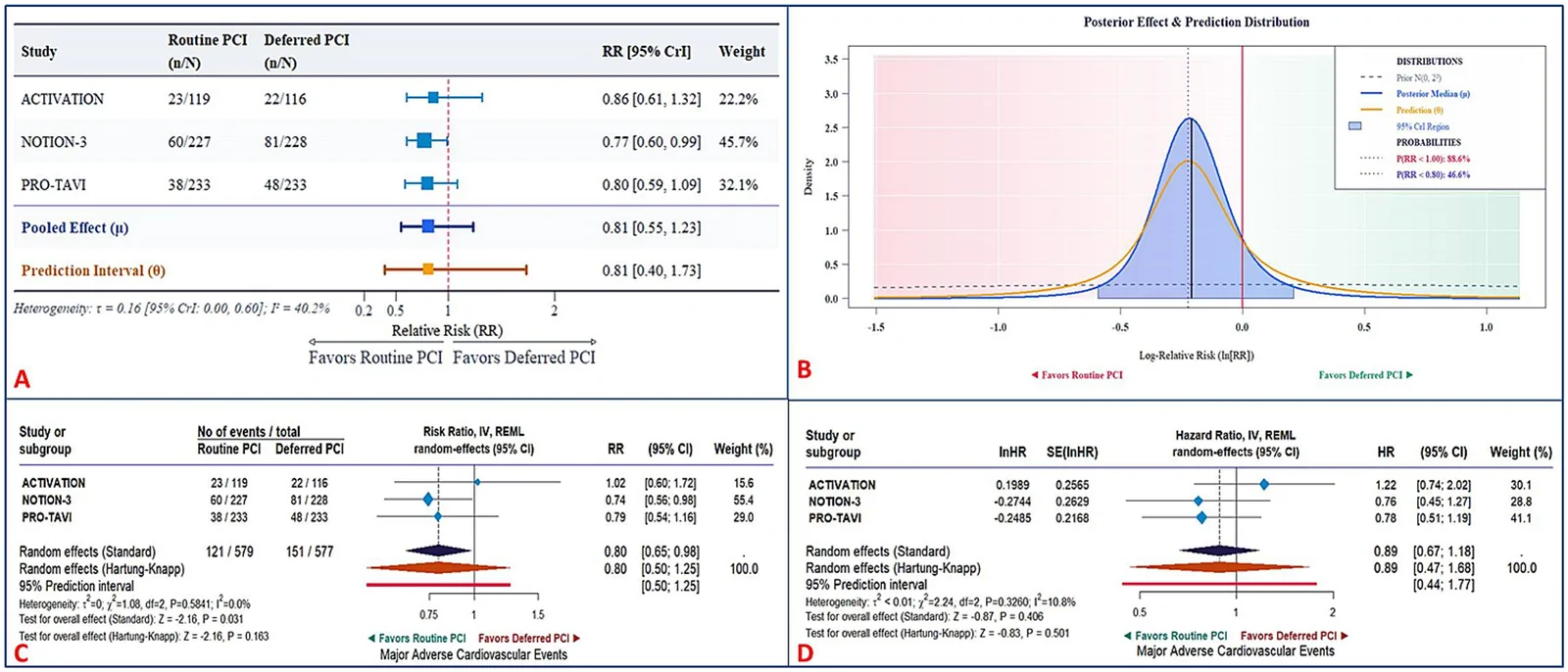
Major Adverse Cardiovascular Events. (A) Bayesian Forest Plot (Shrinkage Estimates). Forest plot showing trial-level posterior shrinkage estimates for MACE comparing Routine percutaneous coronary intervention (PCI) vs. Deferred PCI. Each trial is represented by a point estimate with 95% credible interval (CrI). The pooled posterior median RR and 95% CrI are presented at the bottom. (B) Bayesian Posterior Distribution. Density plot of log-relative risk [ln(RR)] for MACE. The plot displays three distributions: the prior distribution [dashed gray line, N(0, 2^2^)], the posterior distribution of the pooled effect [solid blue line], and the predictive distribution for a future trial [solid orange line]. The shaded blue area indicates the 95% CrI region. The x-axis is annotated to indicate the direction of effect favoring Routine PCI and Deferred PCI. A legend box reports the prior specification and posterior probabilities for P(RR < 1.0) and P(RR < 0.80). (C) Frequentist Forest Plot. Forest plot displaying the RR for MACE for each individual trial with 95% confidence intervals (CIs) [horizontal lines]. The size of each square is proportional to the trial weight in the pooled analysis. The diamond represents the pooled estimate from the random-effects model, with the width of the diamond indicating the 95% CI. (D) Frequentist Forest Plot - 1-Year Landmark Analysis. Forest plot displaying the hazard ratio (HR) for MACE at 1-year follow-up for each individual trial with 95% CIs [horizontal lines]. The size of each square is proportional to the trial weight in the pooled analysis using the inverse variance (IV) method. The diamond represents the pooled estimate, with the width indicating the 95% CI.

#### 3.3.1 Primary efficacy endpoint: spontaneous MI

Across the three included trials, spontaneous MI occurred in 29 of 579 patients (5.0%) with routine PCI versus 36 of 577 patients (6.2%) with deferred PCI. Bayesian random-effects meta-analysis comparing routine versus deferred PCI demonstrated a posterior median RR of 0.84 (95% CrI: 0.43–1.73), with a 70.2% posterior probability of any reduction (RR < 1.0) and a 43.2% probability of a clinically meaningful reduction (RR < 0.8). The 95% prediction interval crossed the null (0.29–2.72), and heterogeneity was low (τ = 0.28, 95% CrI: 0.00–0.81; I^2^ = 26.3%). Sensitivity analyses using alternative priors produced consistent estimates (RR: 0.84–0.86; posterior probability of any reduction: 65.9%–70.2%), supporting the robustness of the primary findings.

In the frequentist random-effects analysis, there was no statistically significant difference between routine and deferred PCI either with or without 95% CI correction (random-effects: RR 0.80, 95% CI: 0.49–1.31; *p* = 0.38; I^2^ = 0.0%; random-effects with HKSJ correction: RR 0.80, 95% CI: 0.27–2.36; *p* = 0.48; Fig. 3). The overall grade of evidence is moderate, downgraded for imprecision attributable to wide intervals (Table S5).

#### 3.3.2 Primary safety endpoint: major bleeding

Major bleeding occurred in 91 of 579 patients (15.7%) assigned to routine PCI versus 57 of 577 patients (9.9%) assigned to deferred PCI. Bayesian random-effects meta-analysis yielded a posterior median RR of 1.56 (95% CrI: 0.91–2.71), with a 95.6% posterior probability of any increased risk (RR > 1.0) and an 83.2% probability of a clinically meaningful increase (RR > 1.25). The 95% prediction interval crossed the null (0.58–4.27), and heterogeneity was moderate (τ = 0.26, 95% CrI: 0.00–0.76; I^2^ = 47.8%). Sensitivity analyses employing alternative priors produced consistent estimates (RR: 1.56 across all models; posterior probability of increased risk: 92.3%–95.6%), affirming the robustness of the primary findings.

In the frequentist random-effects model, routine PCI was associated with a significantly higher risk of major bleeding relative to deferred PCI (RR 1.57, 95% CI: 1.07–2.32; *p* = 0.02), with low-to-moderate between-study heterogeneity (I^2^ = 37.9%). However, after HKSJ correction, the 95% CI widened considerably and was no longer statistically significant (1.57, 95% CI: 0.65–3.81; *p* = 0.16; Fig. 4). The overall grade of evidence is moderate, downgraded for imprecision attributable to wide intervals (Table S5).

### 3.4 Secondary endpoints

Bayesian random-effects meta-analysis demonstrated that routine PCI compared with deferred PCI yielded a median RR of 0.91 (95% CrI: 0.60–1.43) for all-cause mortality, with a 69.6% posterior probability of benefit [PPB] (RR < 1.0). Corresponding estimates for cardiovascular death, any MI, AKI, and stroke were 0.74 (95% CrI: 0.43–1.29; PPB: 87.8%), 0.83 (95% CrI: 0.43–1.75; PPB: 72.2%), 1.02 (95% CrI: 0.41–2.91; PPB: 48.5%), and 0.71 (95% CrI: 0.41–1.25; PPB: 90.0%), respectively.

Routine PCI was associated with a reduced risk of any revascularization (RR 0.29, 95% CrI: 0.12–0.75; PPB: 99.0%) and urgent revascularization (RR 0.37, 95% CrI: 0.15–0.91; PPB: 98.1%). Conversely, bleeding risks were elevated with routine PCI: any bleeding (RR 1.76, 95% CrI: 1.06–2.88; posterior probability of harm [PPH]: 98.1%), life-threatening or disabling bleeding (RR 1.74, 95% CrI: 1.00–3.07; PPH: 97.4%), and minor bleeding (RR 1.96, 95% CrI: 1.09–3.48; PPH: 98.3%) (Figs. S2–S21). Sensitivity analyses using alternative priors (half-normal and half-Cauchy; scales 0.5 and 1.0) confirmed robustness across all secondary endpoints. Effect estimates, posterior probabilities, and heterogeneity estimates remained generally stable (Figs. S22–S31).

Frequentist random-effects models yielded consistent results with Bayesian analyses (Fig. 2). There was no statistically significant difference between routine and deferred PCI for all-cause mortality (*p* = 0.40), cardiovascular mortality (*p* = 0.12), MI (*p* = 0.64), stroke (*p* = 0.07), or AKI (*p* = 0.76). However, routine PCI was associated with a statistically significant reduction in any revascularization (*p* = 0.02) and urgent revascularization (*p* = 0.02). Conversely, routine PCI was associated with a statistically significant increase in any bleeding (*p* < 0.01), disabling or life-threatening bleeding (*p* = 0.01), and minor bleeding (*p* = 0.003). After HKSJ correction, the associations for revascularization and bleeding were no longer statistically significant (Figs. S32–S41).

### 3.5 Health-related and functional outcomes

Two trials reported functional and anginal outcomes [1], [12]. For functional status, the proportion of patients with NYHA class I-II was similar between groups (PCI 88.6% vs deferred 88.7%; RR 1.00, 95% CrI: 0.67–1.49), and NYHA class III-IV occurred in 11.4% of PCI patients vs 11.3% of deferred patients (RR 1.00, 95% CrI: 0.52–1.97). Frequentist analyses demonstrated no statistically significant differences (*p* = 0.94 and *p* = 0.99, respectively).

For anginal symptoms, the proportion of patients with CCS class I-II was numerically lower with routine PCI (10.7% vs 12.8%; RR 0.42, 95% CrI: 0.07–2.41), and CCS class III occurred in 0.3% of PCI patients vs 1.0% of deferred patients (RR 0.43, 95% CrI: 0.07–2.49), though credible intervals were wide. The proportion of patients with no angina (CCS class 0) was similar between groups (88.8% vs 86.2%; RR 1.03, 95% CrI: 0.69–1.54). Frequentist analyses were non-significant for all CCS endpoints. Sensitivity analyses confirmed robustness across all Bayesian models (Figs. S42–S56).

### 3.6 Major adverse cardiovascular events

Under Bayesian random-effects meta-analysis, the median RR for MACE with routine PCI versus deferred PCI was 0.81 (95% CrI: 0.55–1.23), with an 88.6% PPB (RR < 1.0) and a 46.6% probability of a clinically meaningful reduction (RR < 0.8). The 95% prediction interval crossed the null (0.40–1.73). The frequentist pooled analysis yielded an RR of 0.80 (95% CI: 0.65–0.98; *p* = 0.03; I^2^ = 0.0%); however, after HKSJ correction, the estimate was rendered non-significant (*p* = 0.16). In sensitivity analyses, the estimate was attenuated: exclusion of NOTION-3 resulted in an RR of 0.86 (95% CI: 0.63–1.18; *p* = 0.36; I^2^ = 0.0%), with corresponding Bayesian estimates of 0.88 (95% CrI: 0.47–1.68; PPB: 70.4%). Restriction to one-year landmark data to align follow-up durations across trials yielded an HR of 0.89 (95% CI: 0.67–1.18; *p* = 0.41; I^2^ = 10.8%). Bayesian sensitivity analyses using alternative priors confirmed robustness (Figs. 5 and S57).

### 3.7 Trial sequential analysis

In the TSA, the cumulative *Z*-curve did not meet the required information size for the primary endpoints, any revascularization, or all-cause death. In contrast, the analysis was conclusive for any bleeding and for life-threatening or disabling bleeding, or for urgent revascularization, having surpassed the trial sequential monitoring boundaries, which suggests the current evidence is robust against type I and type II errors (Figs. S58–S64).

## 4 Discussion

In the present meta-analysis of 3 RCTs including 1156 patients with concomitant CAD undergoing TAVI, routine revascularization with PCI was not associated with a clear reduction in hard clinical endpoints, including spontaneous MI, stroke, or all-cause mortality, nor did it improve NYHA functional class or CCS angina class. While PCI reduced the need for subsequent revascularization, it was associated with a higher bleeding burden, although the estimate for major bleeding remained imprecise.

The neutral effect of routine PCI on spontaneous MI observed in our analysis challenges the pathophysiological rationale for performing pre-TAVI coronary revascularization. The theoretical concern that untreated significant CAD may predispose patients to peri-procedural or late ischemic events has been a central justification for routine PCI in this population [9], [26]. However, our analysis did not provide clear evidence that this theoretical benefit translates into improved hard ischemic outcomes, with no clear reduction in overall MI, stroke, or cardiovascular death. This interpretation is further supported by the Bayesian analysis, which showed a 70.2% posterior probability of any reduction in spontaneous MI but only a 43.2% probability of a clinically meaningful reduction, indicating substantial residual uncertainty regarding the efficacy of routine PCI.

Several mechanisms may explain this observation. First, the hemodynamic improvements following TAVI, including rapid afterload reduction, regression of left ventricular hypertrophy, and improved coronary perfusion pressure, may mitigate the ischemic burden attributable to fixed coronary stenoses [27], [28]. Second, coronary disease complexity was relatively low in the two trials reporting SYNTAX scores, with median values of 9–10 in NOTION-3 and PRO-TAVI. This relatively limited anatomical burden may partly explain the absence of a clear ischemic benefit from routine PCI. Third, contemporary medical therapy, such as statins and antiplatelets, may provide sufficient plaque stabilization and protection, making revascularization unnecessary in the short term. Fourth, the relatively short follow-up duration in the included studies may be insufficient to capture late ischemic events.

In terms of safety, Routine PCI was associated with a higher risk of major bleeding, although the precision of this effect differed according to the analytical approach. In the Bayesian analysis, the posterior median RR was 1.56 (95% CrI 0.91–2.71), with a 95.6% PPH. The conventional frequentist analysis yielded a similar point estimate, whereas the HKSJ-adjusted CI was wider and crossed the null, underscoring the imprecision associated with the limited number of available trials. The bleeding signal was consistent across secondary bleeding endpoints, including any bleeding (RR 1.76), life-threatening or disabling bleeding (RR 1.74), and minor bleeding (RR 1.96). The mechanisms underlying this excess bleeding cannot be determined from the available aggregate data. Additional antiplatelet exposure following PCI is likely to contribute, but oral anticoagulation use was also common in the study population, being reported in approximately one-third of patients. Therefore, the combination of anticoagulant and antiplatelet therapy may also have contributed to the observed bleeding risk. Differences in bleeding definitions, PCI timing, vascular access, and duration of antithrombotic therapy across trials may further explain the variability in treatment effect. The magnitude of the bleeding effect varied across trials, being greater in PRO-TAVI (RR 2.43) than in NOTION-3 (RR 1.19), contributing to the moderate between-study heterogeneity observed for this endpoint. This variability may be partially explained by differences in bleeding definitions, as NOTION-3 used VARC-2 criteria whereas PRO-TAVI employed VARC-3 definitions. The broader capture of bleeding events under VARC-3 may have contributed to the higher event rates observed in PRO-TAVI [29]. TSA crossed the monitoring boundaries for any bleeding and life-threatening or disabling bleeding, suggesting that these secondary bleeding signals are less likely to be explained by random error under the prespecified TSA assumptions. In contrast, the required information size was not reached for major bleeding, and this primary safety endpoint therefore remains subject to imprecision.

Regarding revascularization, Routine PCI was associated with a lower risk of subsequent revascularization, including both any revascularization (RR 0.29) and urgent revascularization (RR 0.37). However, urgent revascularization should be interpreted differently from hard clinical endpoints. Only 12 versus 38 events were observed, and treatment decisions were made within open-label trials, making this endpoint potentially susceptible to ascertainment and operator-related treatment bias. Although the cumulative *Z*-curve for urgent revascularization crossed the trial sequential monitoring boundary, TSA addresses the risk of random error and does not eliminate the possibility of bias related to endpoint ascertainment or clinical decision-making. This finding is biologically plausible and aligns with the established benefits of coronary revascularization in other clinical contexts; patients with untreated significant coronary stenosis may develop progressive angina or acute coronary syndromes necessitating unplanned revascularization [1]. However, the clinical significance of this finding warrants caution, as the decreased rate of revascularization in the PCI arm did not translate into benefit in either hard clinical outcomes (spontaneous MI, cardiovascular death, or all-cause mortality) or symptom and health-status outcomes.

A critical methodological consideration is the heterogeneity in the definition of MACE across the included trials. While ACTIVATION and PRO-TAVI did not include revascularization in their MACE definition [12], [22], NOTION-3 incorporated urgent revascularization as a component of its primary endpoint [1]. This distinction is highly relevant, as urgent revascularization represents a softer, procedure-driven outcome that may be more susceptible to clinical decision-making and follow-up strategies. Accordingly, the observed reduction in MACE in our pooled analysis was largely driven by the inclusion of the NOTION-3 trial [1]. When this study was excluded, or when analyses were restricted to one-year follow-up, the association was no longer statistically significant (RR 0.86, 95% CI: 0.63–1.18; *p* = 0.36). This attenuation may be interpreted in two ways. From a methodological standpoint, it suggests that differences in follow-up duration across the included trials may have introduced temporal confounding. From a clinical standpoint, it raises the alternative possibility that the clinical benefit of routine PCI, driven predominantly by a reduction in late revascularization events, may require longer follow-up to fully manifest, as evidenced by the progressive divergence in event rates seen in NOTION-3 beyond the first year [1]. Accordingly, the pooled MACE estimate should not be interpreted as evidence of a uniform benefit on hard cardiovascular outcomes.

When considered collectively, these findings highlight a fundamental trade-off between ischemic protection and procedural harm. The PRO-TAVI trial further refines the interpretation of these data by showing that a strategy of PCI deferral is non-inferior to routine PCI before TAVI for a composite endpoint including both ischemic and bleeding events [12]. Importantly, the non-inferiority of the conservative strategy appears to be largely driven by a significant reduction in major bleeding, whereas ischemic events were numerically higher in patients managed without routine revascularization. This observation underscores the importance of endpoint composition, as the inclusion of bleeding events within composite outcomes may substantially influence the overall interpretation of treatment effects.

The available RCTs do not necessarily provide conflicting evidence but rather reflect important differences in study design, patient selection, and lesion assessment. In particular, the definition of significant CAD varies across trials. While ACTIVATION relied solely on angiographic criteria [22], both PRO-TAVI and NOTION-3 permitted physiological assessment (FFR/iFR) for intermediate stenoses [1], [12], with NOTION-3 uniquely mandating revascularization of lesions with stenosis ≥90% or fractional flow reserve (FFR) ≤0.80 [1]. This distinction is clinically relevant, as lesions with demonstrable hemodynamic significance are more likely to benefit from revascularization. The overall complexity of coronary disease also appears to play a key role. In NOTION-3 and PRO-TAVI, median SYNTAX scores were relatively low, reflecting limited anatomical burden, whereas this information was not available for ACTIVATION [1], [12], [22]. This finding is consistent with previous observational studies and meta-analyses, which have reported modest coronary complexity in TAVI populations and have not demonstrated a clear benefit of routine PCI [30], [31]. These differences also have implications for the design of future trials. Standardized criteria for lesion selection should be prespecified, with separate consideration of anatomical severity, lesion location, and physiological significance. In addition, the timing of physiological assessment relative to TAVI should be reported, as severe aortic stenosis itself may influence coronary hemodynamics and the interpretation of functional indices.

In this context, the lack of a mortality benefit with PCI aligns with evidence from patients with stable coronary artery disease, in whom revascularization does not reduce death or MI when added to optimal medical therapy [32], [33], [34], [35]. Another important aspect is the timing of revascularization. The PRO-TAVI trial demonstrated that a deferred strategy is feasible, with a relatively small proportion of patients requiring PCI during follow-up and without major procedural complications [12]. These findings support a staged or selective approach, in which PCI is reserved for patients with persistent symptoms or objective evidence of ischemia. Advances in transcatheter valve technology and implantation techniques, particularly those aimed at preserving coronary access, further enhance the feasibility of this strategy [36], [37].

The 2025 ESC/EACTS guidelines reflect an increasingly selective approach to coronary revascularization in patients undergoing TAVI. PCI should be considered in patients with a primary indication for TAVI and ≥ 90% coronary artery stenosis in a segment with a reference diameter ≥ 2.5 mm (Class IIa, Level B), whereas PCI for ≥70% proximal stenosis in a main vessel may be considered according to symptom status (Class IIb, Level B) [11]. These recommendations are consistent with the concept that the benefit of PCI is likely to depend on lesion severity and patient characteristics rather than on the presence of angiographically significant CAD alone. This distinction is also supported by exploratory analyses from NOTION-3, in which the excess events observed with conservative management of FFR ≤0.80 lesions were driven mainly by higher rates of myocardial infarction and urgent revascularization, whereas outcomes for FFR >0.80 lesions were similar to those who underwent PCI [38].

Clinical Implications.

The findings of this meta-analysis may inform Heart Team decision-making but should not be interpreted as supporting a uniform strategy of PCI deferral. The available randomized evidence primarily applies to stable patients with relatively low-complexity coronary disease, and the wide uncertainty around several pooled estimates indicates that clinically relevant benefit or harm cannot be excluded in all settings. The wide prediction intervals for spontaneous MI and major bleeding, both crossing unity, further caution against extrapolating the pooled estimates to all TAVI candidates. Within the populations represented in the available trials, routine PCI did not show a clear reduction in MI or mortality, while reducing subsequent revascularization at the cost of a higher bleeding burden. These findings therefore provide additional support for an individualized and selective approach to coronary revascularization rather than a change in treatment paradigm. Important evidence gaps remain. Patients with left main disease, proximal left anterior descending disease, complex multivessel CAD, or low-flow low-gradient aortic stenosis were excluded or poorly represented in the available trials, and the present findings should not be extrapolated to these groups. Frailty may represent an additional factor in treatment selection; however, the available evidence is derived from a post hoc analysis of NOTION-3 and should therefore be regarded as hypothesis-generating [39]. Decisions regarding PCI should continue to integrate symptoms, coronary anatomy, ischemic burden, bleeding risk, frailty, and the feasibility of future coronary access after TAVI.

Strengths and Limitations.

Unlike previous pooled analyses [40], this meta-analysis has several important strengths. First, by restricting inclusion to RCTs, we minimized the risk of confounding by indication. Second, the use of complementary frequentist and Bayesian analytic frameworks provided a more comprehensive characterization of uncertainty and enhanced the interpretability of findings for clinicians. Third, the application of TSA offered valuable insights regarding the conclusiveness of the available evidence and the potential need for future randomized trials. Fourth, the comprehensive assessment of bleeding outcomes across the full spectrum of severity provided granular insights into the safety profile of routine PCI that are directly relevant to clinical decision-making.

However, several limitations should be considered. First, the evidence base is limited to three RCTs, two of which were terminated early. This restricts statistical power for low-frequency outcomes and precludes reliable assessment of publication bias. Second, there was heterogeneity in trial design and follow-up. Follow-up was 1 year in ACTIVATION and PRO-TAVI versus a median of 2 years in NOTION-3, and MACE definitions varied, limiting direct comparability. In addition, NOTION-3 and PRO-TAVI incorporated physiological assessment with FFR/iFR, whereas ACTIVATION relied on angiographic criteria. Bleeding definitions and antithrombotic strategies were not fully standardized across trials. Third, generalizability is limited by the exclusion or limited representation of patients with left main disease, high SYNTAX scores, complex multivessel disease, hemodynamic instability, or low-flow low-gradient aortic stenosis. Finally, although trial sequential monitoring boundaries were crossed for selected secondary outcomes, the required information size was not reached for the primary efficacy and safety endpoints. Additional adequately powered randomized trials with longer follow-up are therefore needed.

## 5 Conclusion

Among patients with severe aortic stenosis and concomitant CAD undergoing TAVI, routine PCI, while reducing subsequent revascularization rates, did not lower the risk of ischemic outcomes, including spontaneous MI and stroke, but did substantially increase bleeding complications across the full spectrum of severity. These findings provide additional support for a selective revascularization strategy in carefully selected TAVI candidates with low-complexity coronary disease. Future adequately powered randomized trials with extended follow-up are needed to evaluate long-term outcomes and refine patient selection through physiological and geriatric assessment, particularly in patients with proximal LAD disease, left main disease, complex multivessel CAD, and low-flow, low-gradient aortic stenosis, who were excluded or poorly represented in the available trials.

## CRediT authorship contribution statement

**Ahmed Ibrahim:** Writing – review & editing, Writing – original draft, Resources, Project administration, Methodology, Formal analysis, Conceptualization. **Laila Shalabi:** Writing – review & editing, Writing – original draft, Resources, Methodology, Conceptualization. **Domenico Mario Giamundo:** Writing – review & editing, Writing – original draft, Resources, Methodology. **Hadeel Aboueisha:** Writing – review & editing, Writing – original draft, Resources, Methodology, Funding acquisition. **Ahmed Enani:** Writing – review & editing, Visualization, Resources, Methodology. **Mariam Assi:** Writing – review & editing, Resources, Methodology, Data curation. **Ahmed Mojahed:** Writing – review & editing, Resources, Methodology. **Lucio Giuseppe Granata:** Writing – review & editing, Resources, Methodology, Data curation. **Dan M. Meyer:** Writing – review & editing, Supervision, Methodology. **Pierre Sabouret:** Writing – review & editing, Supervision, Methodology, Conceptualization. **Giuseppe Andò:** Writing – review & editing, Writing – original draft, Supervision, Methodology, Conceptualization.

## Funding

This research did not receive any specific grant from funding agencies in the public, commercial, or not-for-profit sectors.

## Declaration of competing interest

The authors declare that they have no known competing financial interests or personal relationships that could have appeared to influence the work reported in this paper.
