# Supplementary material for: Routine versus deferred percutaneous coronary intervention in patients undergoing transcatheter aortic valve implantation: a frequentist and Bayesian meta-analysis of randomized trials with trial sequential analysis
Source: Int J Cardiol Heart Vasc. 2026 Sep 22;66:102017. doi: 10.1016/j.ijcha.2026.102017 (PMC13634343; doi:10.1016/j.ijcha.2026.102017)

SUPPLEMENTARY APPENDIX

Routine versus Deferred Percutaneous Coronary Intervention in Patients Undergoing Transcatheter Aortic Valve Implantation: A Frequentist and Bayesian Meta-Analysis of Randomized Trials with Trial Sequential Analysis

Ahmed Ibrahim, MBBCh[1]; Laila Shalabi, MBBCh[2]; Domenico Mario Giamundo, MD[3]; Hadeel Aboueisha, MD[4]; Ahmed Enani, MBBCh[1]; Mariam Assi, MBBCh[5]; Ahmed Mojahed, MBBCh[1]; Lucio Giuseppe Granata, MD[6]; Dan M. Meyer, MD[7]; Pierre Sabouret, MD[8]*; Giuseppe Andò, MD, PhD[9,10]*

[1] Faculty of Medicine, Alexandria University, Alexandria, Egypt; [2] Faculty of Medicine, Gharyan University, Gharyan, Libya; [3] Division of Cardiology, Policlinico Casilino, Rome, Italy; [4] Faculty of Medical Sciences, University of Groningen, University Medical Center Groningen, Groningen, The Netherlands; [5] Faculty of Medicine, Al Najah National University, Nablus, Palestine; [6] Cardiology Division, Garibaldi-Nesima Hospital, ARNAS Garibaldi, 95122 Catania, Italy; [7] Department of Cardiothoracic Surgery, Baylor Scott & White Health, Baylor University Medical Center, Dallas, TX, USA; [8] Heart Institute and Action Group, Pitié-Salpétrière, Sorbonne University, Paris, France; [9] Azienda Ospedaliera Papardo, Messina, Italy; [10] Department of Clinical and Experimental Medicine, University of Messina, Messina, Italy

* Drs. Sabouret and Andò are co-senior authors and contributed equally to this work.

This appendix has been provided by the authors to give readers additional information about the work.

Index of Supplementary Material

Item	Title / Content Description	Page
**Table S1**	Comprehensive Literature Search Strategies for Electronic Databases (PubMed, Embase, Scopus, Web of Science)	Page 5
**Table S2**	Definitions of Significant Coronary Artery Disease and Indications for PCI in the Conservative Arms	Page 6
**Table S3**	Detailed Clinical Endpoint Definitions Across Included Randomized Controlled Trials	Page 7
**Table S4**	Additional Baseline Characteristics of Included Studies	Page 10
**Table S5**	GRADE Evidence Profile and Summary of Certainty of Evidence Across Primary and Secondary Endpoints	Page 11
**Figure S1**	Cochrane Risk of Bias 2 (RoB 2) Tool Detailed Domain Evaluations and Summary Plot	Page 13
**Figure S2**	Bayesian Random-Effects Meta-Analysis & Posterior Shrinkage Plot: All-Cause Mortality	Page 13
**Figure S3**	Bayesian Posterior Probability Density Distribution: All-Cause Mortality	Page 13
**Figure S4**	Bayesian Random-Effects Meta-Analysis & Posterior Shrinkage Plot: Cardiovascular Death	Page 14
**Figure S5**	Bayesian Posterior Probability Density Distribution: Cardiovascular Death	Page 14
**Figure S6**	Bayesian Random-Effects Meta-Analysis & Posterior Shrinkage Plot: Total Myocardial Infarction	Page 14
**Figure S7**	Bayesian Posterior Probability Density Distribution: Total Myocardial Infarction	Page 15
**Figure S8**	Bayesian Random-Effects Meta-Analysis & Posterior Shrinkage Plot: Acute Kidney Injury (AKI)	Page 15
**Figure S9**	Bayesian Posterior Probability Density Distribution: Acute Kidney Injury (AKI)	Page 15
**Figure S10**	Bayesian Random-Effects Meta-Analysis & Posterior Shrinkage Plot: Stroke (Any Type)	Page 16
**Figure S11**	Bayesian Posterior Probability Density Distribution: Stroke (Any Type)	Page 16
**Figure S12**	Bayesian Random-Effects Meta-Analysis & Posterior Shrinkage Plot: Any Coronary Revascularization	Page 16
**Figure S13**	Bayesian Posterior Probability Density Distribution: Any Coronary Revascularization	Page 17
**Figure S14**	Bayesian Random-Effects Meta-Analysis & Posterior Shrinkage Plot: Urgent Coronary Revascularization	Page 17
**Figure S15**	Bayesian Posterior Probability Density Distribution: Urgent Coronary Revascularization	Page 17
**Figure S16**	Bayesian Random-Effects Meta-Analysis & Posterior Shrinkage Plot: Any Bleeding	Page 18
**Figure S17**	Bayesian Posterior Probability Density Distribution: Any Bleeding	Page 18

**Figure S18**	Bayesian Random-Effects Meta-Analysis & Posterior Shrinkage Plot: Life-Threatening / Disabling Bleeding	Page 18
**Figure S19**	Bayesian Posterior Probability Density Distribution: Life-Threatening / Disabling Bleeding	Page 18
**Figure S20**	Bayesian Random-Effects Meta-Analysis & Posterior Shrinkage Plot: Minor Bleeding	Page 19
**Figure S21**	Bayesian Posterior Probability Density Distribution: Minor Bleeding	Page 19
**Figure S22**	Bayesian Heterogeneity Prior Sensitivity Analysis: All-Cause Mortality	Page 19
**Figure S23**	Bayesian Heterogeneity Prior Sensitivity Analysis: Cardiovascular Death	Page 19
**Figure S24**	Bayesian Heterogeneity Prior Sensitivity Analysis: Total Myocardial Infarction	Page 20
**Figure S25**	Bayesian Heterogeneity Prior Sensitivity Analysis: Stroke (Any Type)	Page 20
**Figure S26**	Bayesian Heterogeneity Prior Sensitivity Analysis: Acute Kidney Injury (AKI)	Page 20
**Figure S27**	Bayesian Heterogeneity Prior Sensitivity Analysis: Any Coronary Revascularization	Page 20
**Figure S28**	Bayesian Heterogeneity Prior Sensitivity Analysis: Urgent Coronary Revascularization	Page 21
**Figure S29**	Bayesian Heterogeneity Prior Sensitivity Analysis: Any Bleeding	Page 21
**Figure S30**	Bayesian Heterogeneity Prior Sensitivity Analysis: Life-Threatening / Disabling Bleeding	Page 21
**Figure S31**	Bayesian Heterogeneity Prior Sensitivity Analysis: Minor Bleeding	Page 21
**Figure S32**	Frequentist Random-Effects Meta-Analysis (REML vs. HKSJ 95% CIs): All-Cause Mortality	Page 22
**Figure S33**	Frequentist Random-Effects Meta-Analysis (REML vs. HKSJ 95% CIs): Cardiovascular Death	Page 22
**Figure S34**	Frequentist Random-Effects Meta-Analysis (REML vs. HKSJ 95% CIs): Total Myocardial Infarction	Page 22
**Figure S35**	Frequentist Random-Effects Meta-Analysis (REML vs. HKSJ 95% CIs): Stroke (Any Type)	Page 22
**Figure S36**	Frequentist Random-Effects Meta-Analysis (REML vs. HKSJ 95% CIs): Acute Kidney Injury (AKI)	Page 23
**Figure S37**	Frequentist Random-Effects Meta-Analysis (REML vs. HKSJ 95% CIs): Any Coronary Revascularization	Page 23
**Figure S38**	Frequentist Random-Effects Meta-Analysis (REML vs. HKSJ 95% CIs): Urgent Coronary Revascularization	Page 23
**Figure S39**	Frequentist Random-Effects Meta-Analysis (REML vs. HKSJ 95% CIs): Any Bleeding	Page 23
**Figure S40**	Frequentist Random-Effects Meta-Analysis (REML vs. HKSJ 95% CIs): Life-Threatening / Disabling Bleeding	Page 24
**Figure S41**	Frequentist Random-Effects Meta-Analysis (REML vs. HKSJ 95% CIs): Minor Bleeding	Page 24

**Figure S42**	Bayesian Random-Effects Meta-Analysis & Posterior Shrinkage Plot: NYHA Class I–II	Page 24
**Figure S43**	Frequentist Random-Effects Meta-Analysis (REML vs. HKSJ 95% CIs): NYHA Class I–II	Page 24
**Figure S44**	Bayesian Heterogeneity Prior Sensitivity Analysis: NYHA Class I–II	Page 25
**Figure S45**	Bayesian Random-Effects Meta-Analysis & Posterior Shrinkage Plot: NYHA Class III–IV	Page 25
**Figure S46**	Frequentist Random-Effects Meta-Analysis (REML vs. HKSJ 95% CIs): NYHA Class III–IV	Page 25
**Figure S47**	Bayesian Heterogeneity Prior Sensitivity Analysis: NYHA Class III–IV	Page 25
**Figure S48**	Bayesian Random-Effects Meta-Analysis & Posterior Shrinkage Plot: CCS Class 0 (No Angina)	Page 26
**Figure S49**	Frequentist Random-Effects Meta-Analysis (REML vs. HKSJ 95% CIs): CCS Class 0 (No Angina)	Page 26
**Figure S50**	Bayesian Heterogeneity Prior Sensitivity Analysis: CCS Class 0 (No Angina)	Page 26
**Figure S51**	Bayesian Random-Effects Meta-Analysis & Posterior Shrinkage Plot: CCS Class I–II	Page 27
**Figure S52**	Frequentist Random-Effects Meta-Analysis (REML vs. HKSJ 95% CIs): CCS Class I–II	Page 27
**Figure S53**	Bayesian Heterogeneity Prior Sensitivity Analysis: CCS Class I–II	Page 27
**Figure S54**	Bayesian Random-Effects Meta-Analysis & Posterior Shrinkage Plot: CCS Class III	Page 27
**Figure S55**	Frequentist Random-Effects Meta-Analysis (REML vs. HKSJ 95% CIs): CCS Class III	Page 28
**Figure S56**	Bayesian Heterogeneity Prior Sensitivity Analysis: CCS Class III	Page 28
**Figure S57**	Bayesian Heterogeneity Prior Sensitivity Analysis: MACE	Page 29
**Figure S58**	Trial Sequential Analysis (TSA): Spontaneous Myocardial Infarction	Page 29
**Figure S59**	Trial Sequential Analysis (TSA): Major Bleeding (Primary Safety Endpoint)	Page 30
**Figure S60**	Trial Sequential Analysis (TSA): All-Cause Mortality	Page 30
**Figure S61**	Trial Sequential Analysis (TSA): Any Coronary Revascularization	Page 31
**Figure S62**	Trial Sequential Analysis (TSA): Urgent Coronary Revascularization	
**Figure S63**	Trial Sequential Analysis (TSA): Any Bleeding	
**Figure S64**	Trial Sequential Analysis (TSA): Life-Threatening or Disabling Bleeding	

Table S1. **Comprehensive Electronic Search Strategies across Databases (Inception to April 4, 2026)**

Database	Complete Search Strategy	Yield (N)
**PubMed**	((tavi*[Title/Abstract] OR tavr*[Title/Abstract] OR "Transcatheter Aortic Valve*"[Title/Abstract] OR "Percutaneous Aortic Valve*"[Title/Abstract] OR "Transcatheter Heart Valve*"[Title/Abstract])) AND ((PCI[Title/Abstract] OR "percutaneous coronary*"[Title/Abstract] OR "coronary stent*"[Title/Abstract] OR "coronary revascular*"[Title/Abstract] OR "coronary angioplasty"[Title/Abstract] OR PTCA[Title/Abstract] OR "fractional flow reserve"[Title/Abstract] OR "instantaneous wave-free ratio"[Title/Abstract] OR FFR[Title/Abstract] OR iFR[Title/Abstract] OR "drug-eluting stent*"[Title/Abstract] OR "DES"[Title/Abstract])) AND ((random*[Title/Abstract] OR randomized[Title/Abstract] OR randomised[Title/Abstract] OR RCT[Title/Abstract] OR "clinical trial"[Title/Abstract] OR "randomly assigned"[Title/Abstract] OR "randomized controlled trial"[Title/Abstract] OR "prospective study"[Title/Abstract] OR "trial registration"[Title/Abstract]))	**127**
**Scopus**	TITLE-ABS-KEY (((tavi* OR tavr* OR "Transcatheter Aortic Valve*" OR "Percutaneous Aortic Valve*" OR "Transcatheter Heart Valve*")) AND ((PCI OR "percutaneous coronary*" OR "coronary stent*" OR "coronary revascular*" OR "coronary angioplasty" OR PTCA OR "fractional flow reserve" OR "instantaneous wave-free ratio" OR FFR OR iFR OR "drug-eluting stent*" OR "DES")) AND ((random* OR randomized OR randomised OR RCT OR "clinical trial" OR "randomly assigned" OR "randomized controlled trial" OR "prospective study" OR "trial registration"))) AND (EXCLUDE (DOCTYPE , "re") OR EXCLUDE (DOCTYPE , "ed") OR EXCLUDE (DOCTYPE , "le") OR EXCLUDE (DOCTYPE , "no") OR EXCLUDE (DOCTYPE , "cp") OR EXCLUDE (DOCTYPE , "sh") OR EXCLUDE (DOCTYPE , "er") OR EXCLUDE (DOCTYPE , "ch"))	**663**
**Embase**	(tavi*:ti,ab,kw OR tavr*:ti,ab,kw OR 'transcatheter aortic valve*':ti,ab,kw OR 'percutaneous aortic valve*':ti,ab,kw OR 'transcatheter heart valve*':ti,ab,kw) AND (pci:ti,ab,kw OR 'percutaneous coronary*':ti,ab,kw OR 'coronary stent*':ti,ab,kw OR 'coronary revascular*':ti,ab,kw OR 'coronary angioplasty':ti,ab,kw OR ptca:ti,ab,kw OR 'fractional flow reserve':ti,ab,kw OR 'instantaneous wave-free ratio':ti,ab,kw OR ffr:ti,ab,kw OR ifr:ti,ab,kw OR 'drug-eluting stent*':ti,ab,kw OR 'des':ti,ab,kw) AND (random*:ti,ab,kw OR randomized:ti,ab,kw OR randomised:ti,ab,kw OR rct:ti,ab,kw OR 'clinical trial':ti,ab,kw OR 'randomly assigned':ti,ab,kw OR 'randomized controlled trial':ti,ab,kw OR 'prospective study':ti,ab,kw OR 'trial registration':ti,ab,kw)	**261**
**Web of Science**	((tavi* OR tavr* OR "Transcatheter Aortic Valve*" OR "Percutaneous Aortic Valve*" OR "Transcatheter Heart Valve*")) AND ((PCI OR "percutaneous coronary*" OR "coronary stent*" OR "coronary revascular*" OR "coronary angioplasty" OR PTCA OR "fractional flow reserve" OR "instantaneous wave-free ratio" OR FFR OR iFR OR "drug-eluting stent*" OR "DES")) AND ((random* OR randomized OR randomised OR RCT OR "clinical trial" OR "randomly assigned" OR "randomized controlled trial" OR "prospective study" OR "trial registration")) (Topic)	**148**
**Total Records Identified**		**1,199**

Note: Search executed across electronic bibliographic databases with inception through April 4, 2026 without language restrictions.

Table S2. Definitions of Significant Coronary Artery Disease across Included Trials

Trial	Angiographic Criteria	Physiological Criteria	Additional Protocol Notes
**ACTIVATION**	≥70% diameter stenosis in a major epicardial coronary artery (or ≥50% if protected left main stem or vein graft)	Not used	Canadian Cardiovascular Society (CCS) angina class ≤2 required; unprotected left main disease excluded.
**NOTION-3**	≥90% diameter stenosis by visual assessment	FFR ≤0.80	Lesions with 50%–89% stenosis required FFR assessment; complete revascularization intended.
**PRO-TAVI**	70%–99% stenosis by visual estimation in a native coronary artery (≥2.5 mm vessel diameter) or bypass graft	FFR, iFR, or RFR for intermediate 40%–70% stenosis	Unprotected left main or left-main equivalent excluded; solitary CTO excluded.

Abbreviations: CCS = Canadian Cardiovascular Society; CTO = chronic total occlusion; FFR = fractional flow reserve; iFR = instantaneous wave-free ratio; RFR = resting full-cycle ratio; TAVI = transcatheter aortic valve implantation.

Table S3. **Detailed Clinical Endpoint Definitions across Included Randomized Controlled Trials**

Clinical Outcome / Domain	ACTIVATION Trial	NOTION-3 Trial	PRO-TAVI Trial
**Classification Framework**	VARC-2 Criteria	VARC-2 Criteria	VARC-3 Criteria
**Primary Endpoint Definition**	Composite of all-cause mortality and all-cause rehospitalization at 1 year post-TAVR.	Composite of all-cause mortality, myocardial infarction (MI), or urgent revascularization at a median follow-up of 2 years.	Composite of all-cause mortality, myocardial infarction (MI), stroke, and major bleeding (VARC-3 Type 2–4) at 1 year post-randomization.
**Spontaneous Myocardial Infarction**	>72 h post-procedure:<br>• Cardiac biomarkers (preferably troponin) rise/fall >99th percentile URL AND<br>• Objective evidence of myocardial ischemia (symptoms, new ST-T changes/LBBB, new contiguous Q-waves, or new loss of viable myocardium / regional wall motion abnormality).	>72 h post-TAVR / >48 h post-PCI:<br>• Troponin >99th percentile URL (if baseline elevated, ≥20% rise required) AND<br>• Objective evidence of ischemia from ≥1 feature: ischemic symptoms, new ischemic ECG changes in 2 contiguous leads, new pathological contiguous Q-waves, imaging loss of viable myocardium/wall motion abnormality, sudden cardiac death with ischemic signs/thrombus, or autopsy evidence of MI.	Type 1 MI (>48 h post-procedure):<br>• Rise/fall of cTn >99th percentile URL AND ≥1 criterion: acute ischemic symptoms, new ischemic ECG changes (ST-T or new LBBB), new pathological Q-waves in ≥2 contiguous leads, imaging loss of viable myocardium/wall motion abnormality, or coronary thrombus on angiography/autopsy.<br>Type 2 MI: cTn >99th percentile URL with supply/demand mismatch without acute coronary thrombosis.
**Peri-Procedural Myocardial Infarction**	≤72 h post-procedure:<br>• Peak biomarkers >15× URL for troponin or >5× URL for CK-MB (if baseline elevated, ≥50% post-procedure increase required) AND<br>• New ischemic symptoms or signs (ventricular arrhythmias, new/worsening HF, new ST-segment changes, hemodynamic instability, new contiguous Q-waves, imaging loss).	1. Post-TAVR (<72 h): Troponin >15× URL or CK-MB >5× URL (≥50% rise if baseline elevated) + ≥1: symptoms, arrhythmia, HF, ECG changes, hemodynamic instability, imaging.<br>2. Post-PCI (<48 h): Troponin or CK-MB >5× URL (≥20% rise if baseline elevated) + ≥1: symptoms, ECG changes, imaging, flow-limiting angiographic complication.<br>3. Post-CABG (<48 h): Troponin or CK-MB >10× URL (≥20% rise if baseline elevated) + new Q-waves/LBBB, imaging loss, graft/artery occlusion.	• Type 4A (PCI ≤48 h): In normal baseline CK: peak CK within 48 h ≥10× ULN (or ≥5× ULN + ischemic feature). Normal baseline cTn (no CK): cTn ≥70× ULN (or ≥35× ULN + ischemic feature). Elevated baseline: absolute increment to above levels + new ECG changes.<br>• Type 4B: Stent thrombosis (acute 0–24h, subacute 24h–30d, late 30d–1y, very late >1y).<br>• Type 5 (TAVR/SAVR/CABG ≤48 h): Same CK (≥10× / ≥5×) or cTn (≥70× / ≥35×) thresholds and ischemic criteria as Type 4A.
**Major Bleeding**	• Overt bleeding with hemoglobin drop ≥3.0 g/dL<br>• Requiring transfusion of 2–3 units of PRBC/whole blood<br>• Causing hospitalization or permanent injury, or requiring surgery<br>• BARC Type 3a (does not meet life-threatening criteria).	• Overt bleeding with hemoglobin drop ≥3.0 g/dL<br>• Requiring transfusion of 2–3 units of packed red blood cells<br>• Requiring surgical intervention<br>• BARC Type 3a.	VARC-3 Type 2 Major Bleeding:<br>• Overt bleeding requiring transfusion of 2–4 units of whole blood / RBCs (BARC 3a)<br>• Overt bleeding associated with hemoglobin drop ≥3.0 g/dL but <5.0 g/dL (>1.86 to <3.1 mmol/L) (BARC 3a).
**Life-Threatening / Disabling / Fatal Bleeding**	Any of:<br>• Fatal bleeding (BARC 5)<br>• Bleeding in a critical organ (intracranial, intraspinal, intraocular, pericardial with pericardiocentesis, intramuscular with compartment syndrome; BARC 3b/3c)	Any of:<br>• Fatal bleeding<br>• Bleeding into critical organs or causing shock<br>• Overt bleeding with hemoglobin drop >5.0 g/dL or transfusion >4 units.	VARC-3 Type 3 (Life-Threatening):<br>• Bleeding in critical organ / compartment syndrome (BARC 3b, 3c)<br>• Hypovolemic shock or severe hypotension (SBP <90 mmHg >30 min) requiring vasopressors/surgery<br>• Reoperation / surgical exploration for

	• Bleeding causing hypovolemic shock or severe hypotension requiring vasopressors/surgery (BARC 3b)<br>• Hemoglobin drop ≥5.0 g/dL or transfusion ≥4 units (BARC 3b).		bleeding<br>• Post-thoracotomy chest tube output ≥2 L / 24 h (BARC 4)<br>• Transfusion ≥5 units (BARC 3a) or Hb drop ≥5.0 g/dL (BARC 3b).<br>VARC-3 Type 4: Overt bleeding leading to death (probable or definite).
**Minor Bleeding**	Any clinically relevant overt bleeding (e.g., access-site hematoma) that does not qualify as life-threatening, disabling, or major (BARC 2).	Clinically overt bleeding requiring non-surgical medical intervention, causing hospitalization, or warranting formal medical evaluation; not qualifying as major or life-threatening (BARC 2).	VARC-3 Type 1 Minor Bleeding:<br>• Overt bleeding requiring medical intervention by a healthcare professional (hospitalization, escalated care, or medical evaluation; BARC 2)<br>• Overt bleeding requiring transfusion of exactly 1 unit of whole blood/PRBC (BARC 3a).
**Cardiovascular Mortality & Causes of Death**	-	Deaths from proximate cardiac causes (MI, tamponade, worsening HF), non-coronary vascular conditions (stroke, pulmonary embolism, aortic causes), procedural/valve-related deaths, sudden or unwitnessed deaths, or deaths of unknown cause.	Deaths related to heart failure, cardiogenic shock, bioprosthetic valve dysfunction (structural deterioration, non-structural dysfunction, thrombosis, endocarditis), MI, stroke, thromboembolism, bleeding, tamponade, vascular complication, arrhythmia, infection; intraprocedural death; sudden death; or death of unknown cause.
**Stroke & Neurological Events**	• Stroke: Acute focal/global neurological deficit lasting ≥24 h, or <24 h if neuroimaging documents new infarction/hemorrhage, or resulting in death.<br>• Disabling stroke: mRS ≥2 at 90 days and increase ≥1 from baseline.<br>• TIA: Focal deficit <24 h without neuroimaging infarct/hemorrhage.	• Stroke: Acute focal or global neurological deficit lasting >24 h, or duration <24 h with neuroimaging documentation of new CNS hemorrhage or infarction.	• Ischemic Stroke: Acute signs/symptoms conforming to vascular territory lasting ≥24 h or <24 h with neuroimaging/pathology proof of CNS infarction (NeuroARC Type 1a/1aH).<br>• Hemorrhagic Stroke: Non-traumatic intracranial/subarachnoid bleed.<br>• Stroke NOS: Persisting ≥24 h without definite etiology.<br>• TIA: Transient focal deficit <24 h without infarction.
**Coronary Revascularization**	Unplanned coronary revascularization (PCI or CABG) performed during the follow-up period.	• Any Revascularization: Any unplanned PCI or CABG performed after random allocation.<br>• Urgent Revascularization: Any revascularization performed during an unplanned hospitalization for acute coronary syndrome (MI or unstable angina with objective ischemia).	Classified by urgency per ACC/AHA:<br>• Elective: Outpatient/scheduled.<br>• Urgent: Inpatient before discharge due to risk of ischemia, MI, or death.<br>• Emergency: Immediate procedure due to imminent mortality risk.<br>• Salvage: Last-resort / cardiogenic shock.<br>Subdivided by target per ARC-2: Study Lesion (±5 mm margin), Study Vessel, Non-Study Vessel.
**Functional & Angina Classification**	-	• NYHA Functional Class (I–IV)<br>• CCS Angina Class (0–IV: Class 0 = no angina, Class 1 = strenuous exertion, Class 2 = slight limit, Class 3 = marked limit, Class 4 = rest angina)	• NYHA Functional Class (I–IV)<br>• CCS Angina Class (0–IV)

**Major Adverse Cardiovascular Events (MACE)**	Composite of all-cause mortality, myocardial infarction, stroke, or acute kidney injury at 1 year post-procedure.	Composite of all-cause mortality, myocardial infarction, or urgent revascularization up to a median follow-up of 2 years.	Secondary composite of all-cause mortality, myocardial infarction, and stroke at 1 year post-randomization.

Abbreviations: ACC/AHA = American College of Cardiology / American Heart Association; AKI = acute kidney injury; ARC-2 = Academic Research Consortium-2; BARC = Bleeding Academic Research Consortium; CABG = coronary artery bypass grafting; CAD = coronary artery disease; CCS = Canadian Cardiovascular Society; CK = creatine kinase; CK-MB = creatine kinase-myocardial band; CNS = central nervous system; cTn = cardiac troponin; DAPT = dual antiplatelet therapy; ECG = electrocardiogram; FFR = fractional flow reserve; HF = heart failure; LAD = left anterior descending artery; LBBB = left bundle branch block; MACE = major adverse cardiovascular events; MI = myocardial infarction; mRS = modified Rankin Scale; NeuroARC = Neurological Academic Research Consortium; NOS = not otherwise specified; NYHA = New York Heart Association; PCI = percutaneous coronary intervention; PRBC = packed red blood cells; SAVR = surgical aortic valve replacement; SBP = systolic blood pressure; TAVI = transcatheter aortic valve implantation; TIA = transient ischemic attack; ULN = upper limit of normal; URL = upper reference limit; VARC-2 / VARC-3 = Valve Academic Research Consortium-2 / Consortium-3.

Table S4. **Additional Baseline Clinical, Procedural, and Echocardiographic Characteristics of Included Studies**

Characteristic	ACTIVATION		NOTION-3		PRO-TAVI	
	Routine PCI	Deferred PCI	Routine PCI	Deferred PCI	Routine PCI	Deferred PCI
**PCI Timing Relative to TAVI**						
• Before TAVI	100% (116/116)	—	74% (163/219)	—	93% (213/229)	—
• Concomitant with TAVI	0% (0/116)	—	17% (37/219)	—	7% (15/229)	—
• After TAVI	0% (0/116)	—	9% (19/219)	—	—	—
**Procedural Intervals & Timelines**						
**Time to TAVI, median (IQR)**	41 days	27 days	34 days (7–62)	25 days (2–54)	31 days (14–47)	25 days (12–43)
**Time from Randomization to PCI, median (IQR)**	—	—	9 days (1–26)	—	13 days (6–23)	—
**Vascular Access Route**						
• Transfemoral	89% (98/110)	80% (86/107)	97% (212/219)	98% (220/225)	93% (214/230)	93% (214/229)
• Other / Non-transfemoral access	11% (12/110)	20% (21/107)	3% (7/219)	2% (5/225)	7% (16/230)	7% (15/229)
**Antithrombotic Therapy**						
**Baseline Oral Anticoagulant (OAC) Use**	26% (31/119)	16% (18/116)	36% (82/227)	32% (74/228)	37% (86/233)	36% (83/233)

Abbreviations: IQR = interquartile range; OAC = oral anticoagulant; PCI = percutaneous coronary intervention; TAVI = transcatheter aortic valve implantation. Data presented as % (n/N) or median (IQR).

Table S5. GRADE Evidence Profile and Summary of Certainty of Evidence across Primary and Secondary Endpoints

Outcome	№ of Studies (Patients)	Certainty Assessment (GRADE Domains)					Event Rates (%)		Effect Estimates		Certainty	Importance
		Risk of Bias	Inconsistency	Indirectness	Imprecision	Other	Routine PCI	Deferred PCI	Relative Effect (95% CI)	Absolute Effect (95% CI)		
**Primary Efficacy Outcome**												
**Spontaneous Myocardial Infarction**	3 RCTs (1,156)	Not serious[1]	Not serious[2] (I^2=0%)	Not serious	Serious[5]	None	29/579 (5.0%)	36/577 (6.2%)	RR 0.80 (0.49 to 1.31)	12 fewer per 1,000 (from 32 fewer to 19 more)	⊕⊕⊕◯ MODERATE	CRITICAL
**Primary Safety Outcome**												
**Major Bleeding**	3 RCTs (1,156)	Not serious[1]	Not serious[2] (I^2=38%)	Not serious	Serious[6]	None	91/579 (15.7%)	57/577 (9.9%)	RR 1.57 (1.07 to 2.32)	56 more per 1,000 (from 7 more to 131 more)	⊕⊕⊕◯ MODERATE	CRITICAL
**Secondary Clinical Outcomes**												
**All-cause Mortality**	3 RCTs (1,156)	Not serious[1]	Not serious[2] (I^2=0%)	Not serious	Serious[5]	None	94/579 (16.2%)	104/577 (18.0%)	RR 0.90 (0.70 to 1.15)	18 fewer per 1,000 (from 54 fewer to 27 more)	⊕⊕⊕◯ MODERATE	CRITICAL
**Cardiovascular Death**	3 RCTs (1,156)	Not serious[1]	Not serious[2] (I^2=0%)	Not serious	Serious[5]	None	43/579 (7.4%)	58/577 (10.1%)	RR 0.74 (0.51 to 1.08)	26 fewer per 1,000 (from 49 fewer to 8 more)	⊕⊕⊕◯ MODERATE	CRITICAL
**Stroke**	3 RCTs (1,156)	Not serious[1]	Not serious[2] (I^2=0%)	Not serious	Serious[5]	None	40/579 (6.9%)	57/577 (9.9%)	RR 0.70 (0.48 to 1.03)	30 fewer per 1,000 (from 51 fewer to 3 more)	⊕⊕⊕◯ MODERATE	CRITICAL
**Myocardial Infarction (Spontaneous or Procedural)**	3 RCTs (1,156)	Not serious[1]	Not serious[2] (I^2=50%)	Not serious	Serious[5]	None	37/579 (6.4%)	48/577 (8.3%)	RR 0.86 (0.45 to 1.63)	12 fewer per 1,000 (from 46 fewer to 52 more)	⊕⊕⊕◯ MODERATE	IMPORTANT
**Any Revascularization**	3 RCTs (1,156)	Serious[1]	Serious[3] (I^2=66%)	Not serious	Serious[5]	None	17/579 (2.9%)	72/577 (12.5%)	RR 0.28 (0.09 to 0.85)	90 fewer per 1,000 (from 19 fewer to 114 fewer)	⊕◯◯◯ VERY LOW	IMPORTANT
**Urgent Revascularization**	3 RCTs (1,156)	Serious[1]	Not serious[2] (I^2=17%)	Not serious	Serious[5]	None	12/579 (2.1%)	38/577 (6.6%)	RR 0.37 (0.16 to 0.86)	42 fewer per 1,000 (from 9 fewer to 55 fewer)	⊕⊕◯◯ LOW	IMPORTANT
**Any Bleeding**	3 RCTs (1,156)	Not serious[1]	Serious[3] (I^2=67%)	Not serious	Serious[6]	None	215/579 (37.1%)	118/577 (20.5%)	RR 1.77 (1.27 to 2.47)	158 more per 1,000 (from 55 more to 301 more)	⊕⊕◯◯ LOW	IMPORTANT

**Life-threatening / Disabling Bleeding**	3 RCTs (1,156)	Not serious[1]	Not serious[2] (I^2=0%)	Not serious	Not serious	None	60/579 (10.4%)	34/577 (5.9%)	RR 1.74 (1.16 to 2.61)	44 more per 1,000 (from 9 more to 95 more)	⊕⊕⊕◯ **MODERATE**	**CRITICAL**
**Minor Bleeding**	3 RCTs (1,156)	Not serious[1]	Serious[3] (I^2=61%)	Not serious	Not serious	None	141/579 (24.4%)	70/577 (12.1%)	RR 1.98 (1.27 to 3.10)	119 more per 1,000 (from 33 more to 254 more)	⊕⊕⊕◯ **MODERATE**	**IMPORTANT**
**Acute Kidney Injury**	3 RCTs (1,156)	Not serious[1]	Serious[3] (I^2=76%)	Not serious	Serious[5]	None	27/579 (4.7%)	32/577 (5.5%)	RR 1.22 (0.34 to 4.31)	12 more per 1,000 (from 36 fewer to 182 more)	⊕⊕◯◯ **LOW**	**IMPORTANT**
**Major Adverse Cardiovascular Events (MACE)**	3 RCTs (1,156)	Not serious[1]	Not serious[2] (I^2=0%)	Very Serious[4]	Not serious	None	121/579 (20.9%)	151/577 (26.2%)	RR 0.80 (0.65 to 0.98)	52 fewer per 1,000 (from 5 fewer to 92 fewer)	⊕⊕◯◯ **LOW**	**CRITICAL**

Abbreviations: CI, confidence interval; PCI, percutaneous coronary intervention; RR, risk ratio.

Explanations and GRADE Footnotes:

[1] Risk of Bias: Not serious for most clinical outcomes based on RoB 2; downgraded 1 level for revascularization outcomes due to unblinded, open-label design where operator awareness of untreated bystander stenosis directly influences discretionary catheterization/revascularization decisions.

[2] Inconsistency: Not serious; low-to-moderate heterogeneity with a consistent direction of effect across trials ($I^2 < 50\%$).

[3] Inconsistency: Serious; substantial statistical heterogeneity observed across trials ($I^2 > 50\%$).

[4] Indirectness: Very serious; the definition of Major Adverse Cardiovascular Events (MACE) varied substantially across trials. This definitional heterogeneity introduces indirectness because the composite outcome does not represent the same construct across all trials. When NOTION-3 was excluded in a sensitivity analysis, the effect was attenuated and was no longer statistically significant (risk ratio 0.86, 95% CI 0.63–1.18; $p = 0.36$).

[5] Imprecision: Serious; the 95% confidence interval crosses the null value of 1.0 (indicating uncertainty about both direction and magnitude of effect) or total event counts are sparse.

[6] Imprecision (Trial Sequential Analysis & Required Information Size): Serious; although nominal statistical significance was reached ($p < 0.05$), Trial Sequential Analysis demonstrates that the cumulative evidence base has not crossed the Required Information Size (RIS) monitoring boundaries, and the lower CI boundary is adjacent to minimal clinical risk.

Figure S1. **Cochrane Risk of Bias 2 (RoB 2) Tool Detailed Domain Evaluations and Summary Plot**

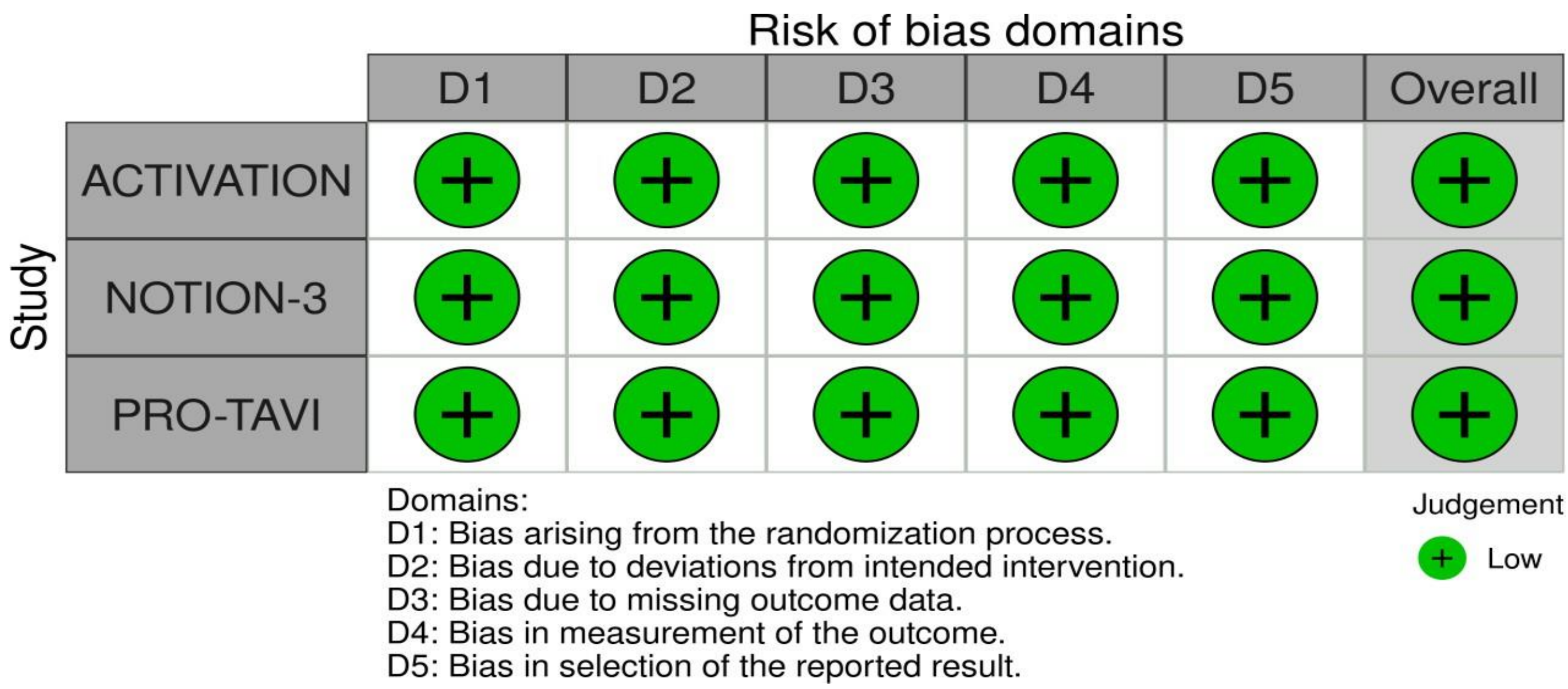

Figure S2. **Bayesian Random-Effects Meta-Analysis & Posterior Shrinkage Plot: All-Cause Mortality.**

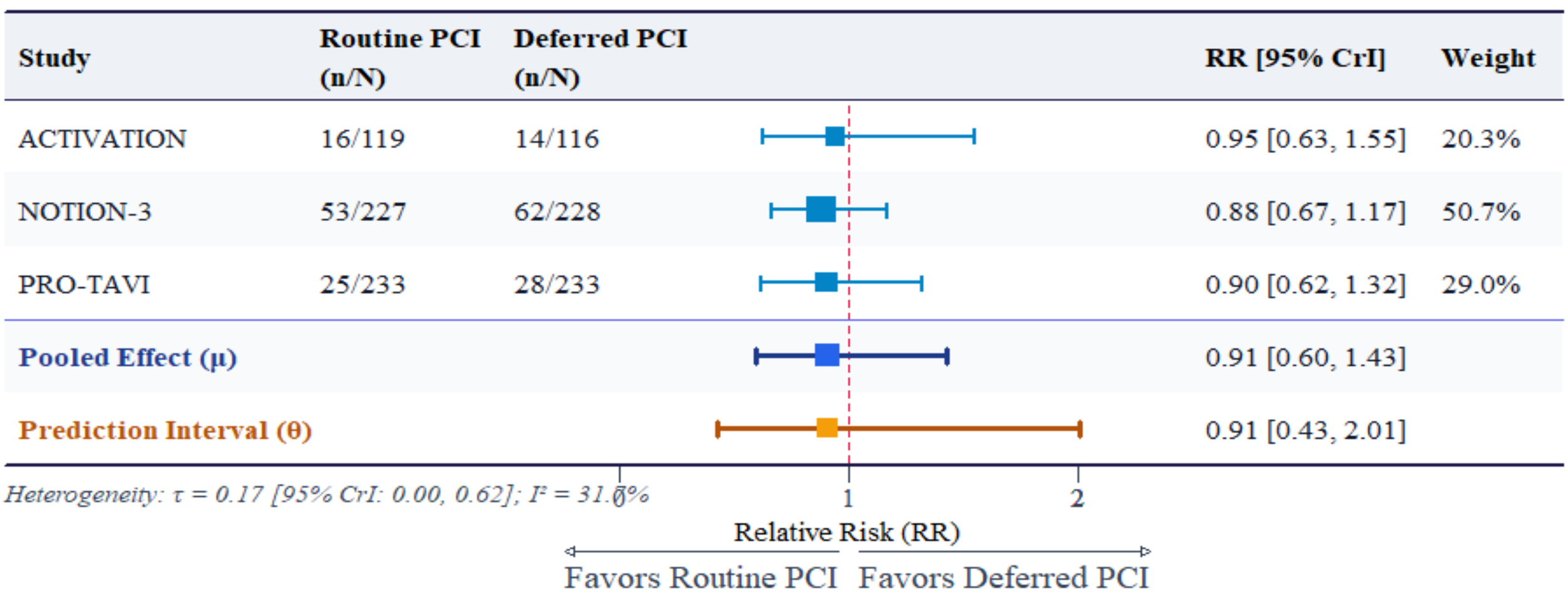

Figure S3. **Bayesian Posterior Probability Density Distribution: All-Cause Mortality**

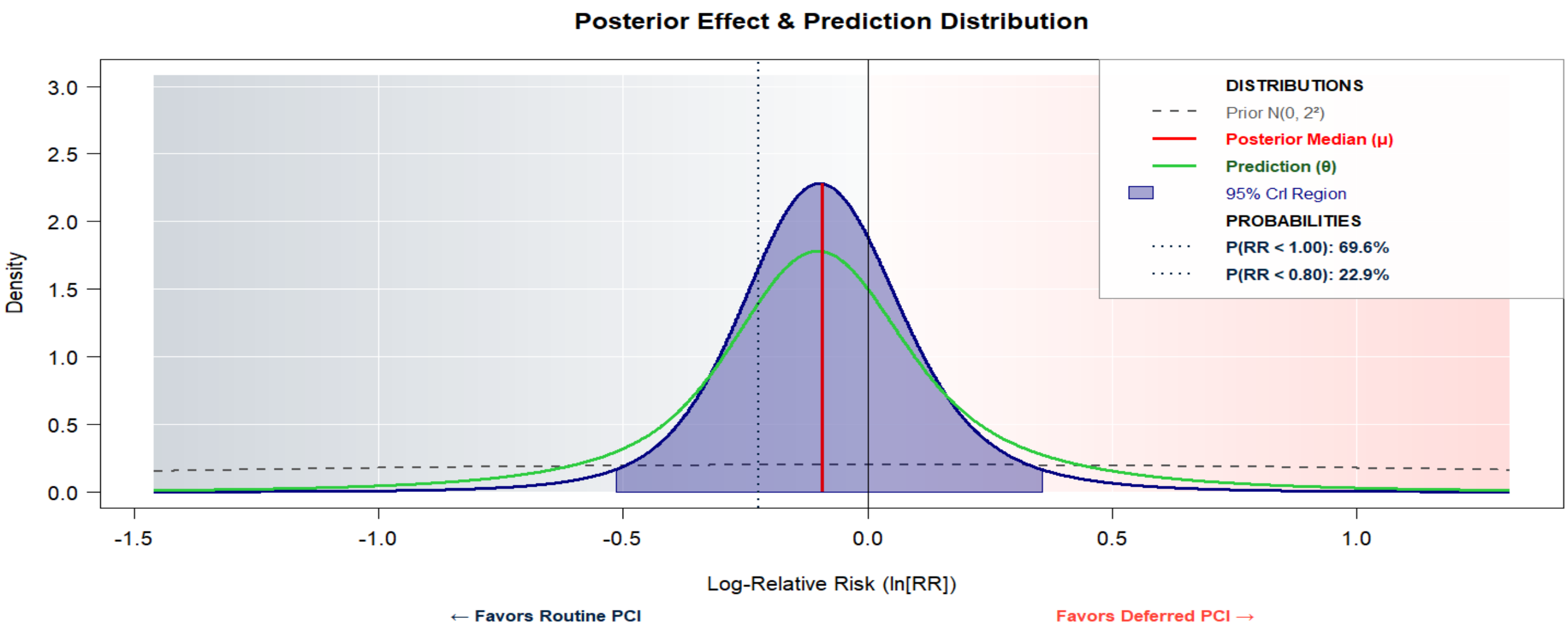

Figure S4. **Bayesian Random-Effects Meta-Analysis & Posterior Shrinkage Plot: Cardiovascular Death**

Study	Routine PCI (n/N)	Deferred PCI (n/N)	RR [95% CrI]	Weight
ACTIVATION	7/119	11/116	0.72 [0.39, 1.27]	21.6%
NOTION-3	20/227	30/228	0.72 [0.46, 1.11]	44.5%
PRO-TAVI	16/233	17/233	0.80 [0.49, 1.36]	34.0%
**Pooled Effect (μ)**			0.74 [0.43, 1.29]	
**Prediction Interval (θ)**			0.74 [0.30, 1.87]	

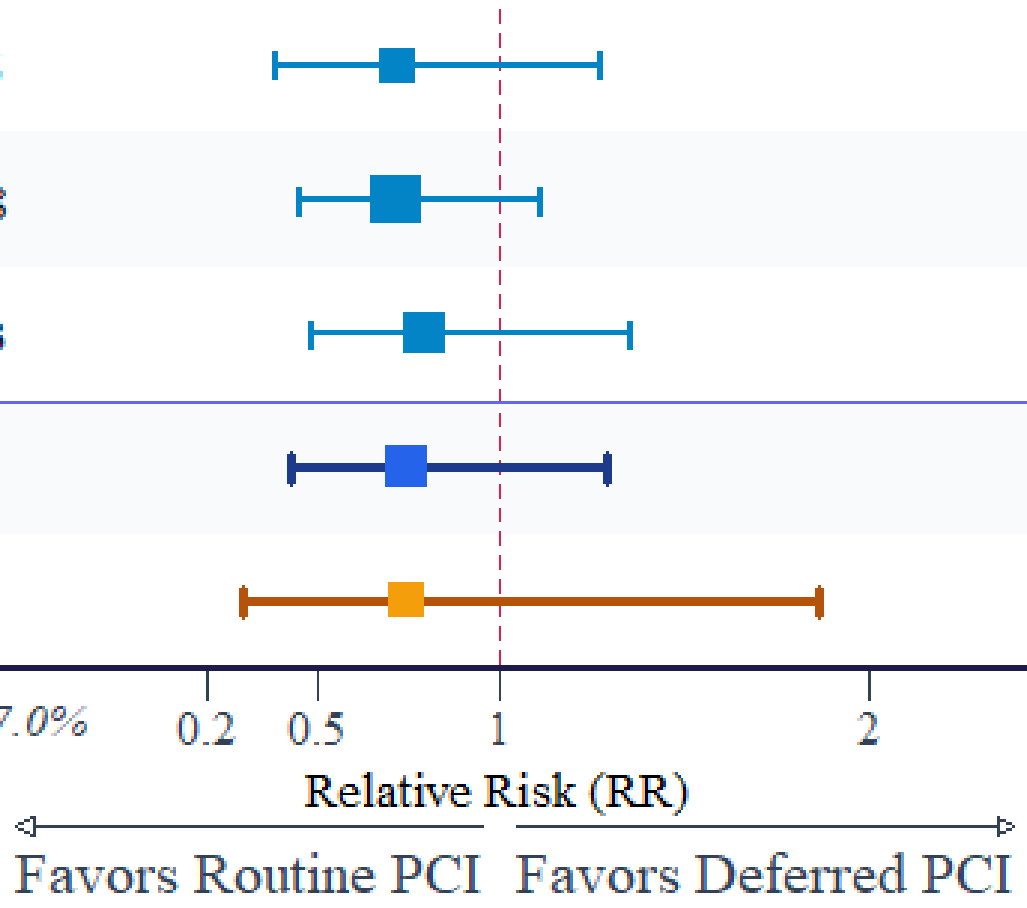

Heterogeneity: τ = 0.21 [95% CrI: 0.00, 0.70]; I² = 27.0%

Figure S5. **Bayesian Posterior Probability Density Distribution: Cardiovascular Death**

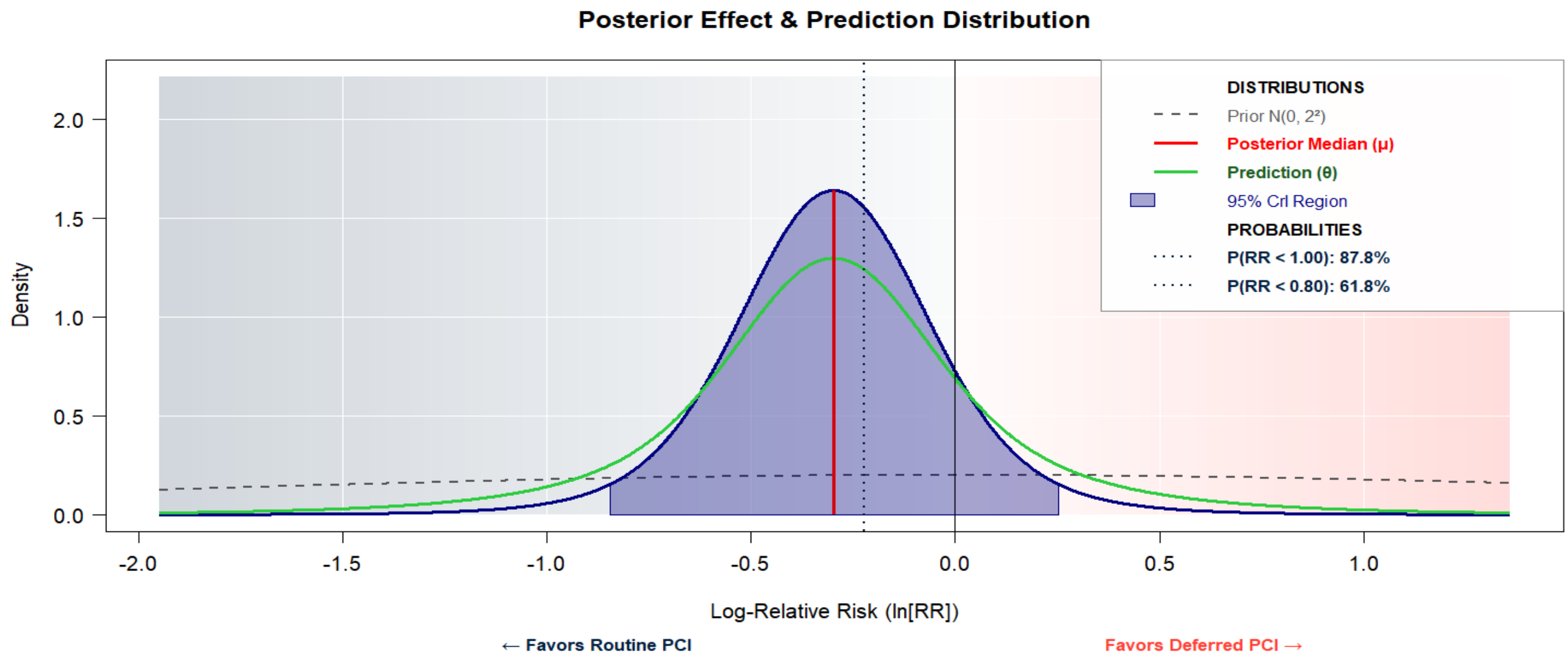

Figure S6. **Bayesian Random-Effects Meta-Analysis & Posterior Shrinkage Plot: Total Myocardial Infarction**

Study	Routine PCI (n/N)	Deferred PCI (n/N)	RR [95% CrI]	Weight
ACTIVATION	8/119	4/116	0.99 [0.48, 2.67]	19.6%
NOTION-3	17/227	31/228	0.67 [0.39, 1.11]	46.9%
PRO-TAVI	12/233	13/233	0.85 [0.47, 1.61]	33.5%
**Pooled Effect (μ)**			0.83 [0.43, 1.75]	
**Prediction Interval (θ)**			0.82 [0.26, 3.01]	

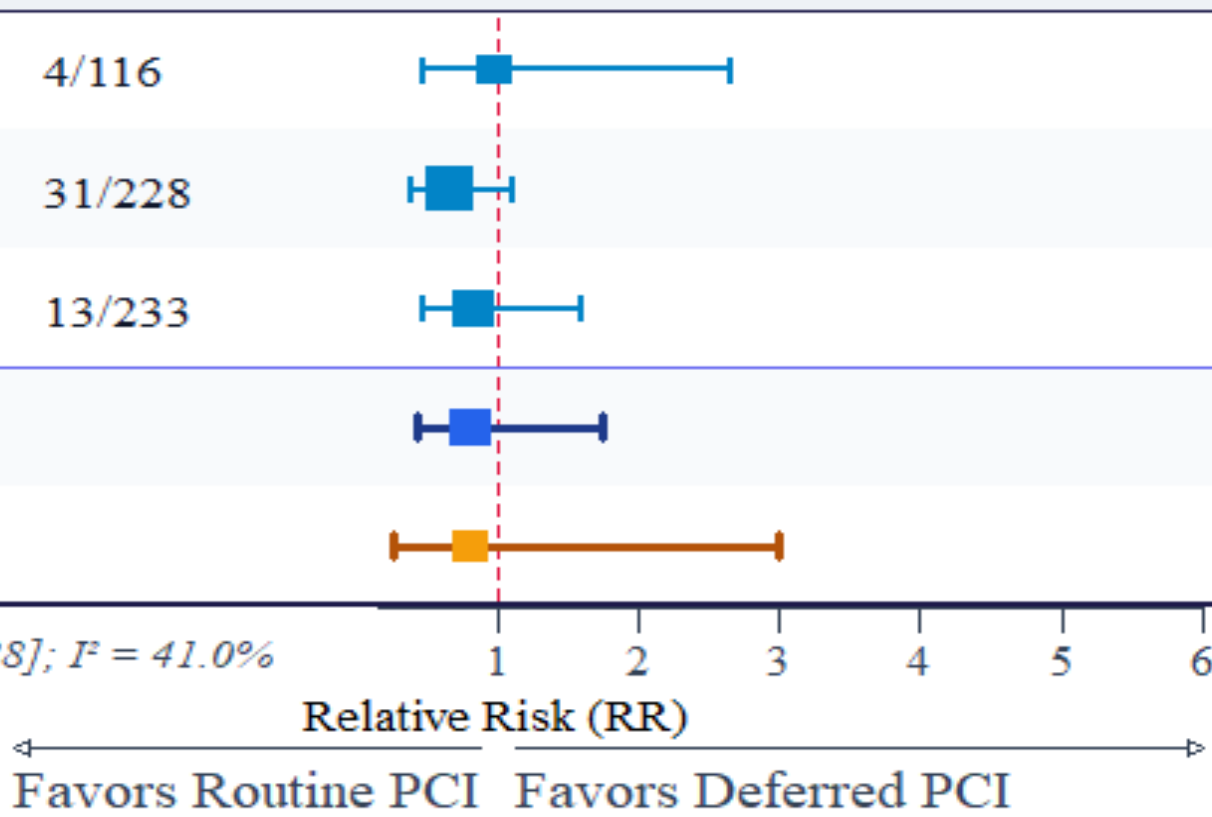

Heterogeneity: τ = 0.34 [95% CrI: 0.00, 0.88]; I² = 41.0%

Figure S7. Bayesian Posterior Probability Density Distribution: Total Myocardial Infarction

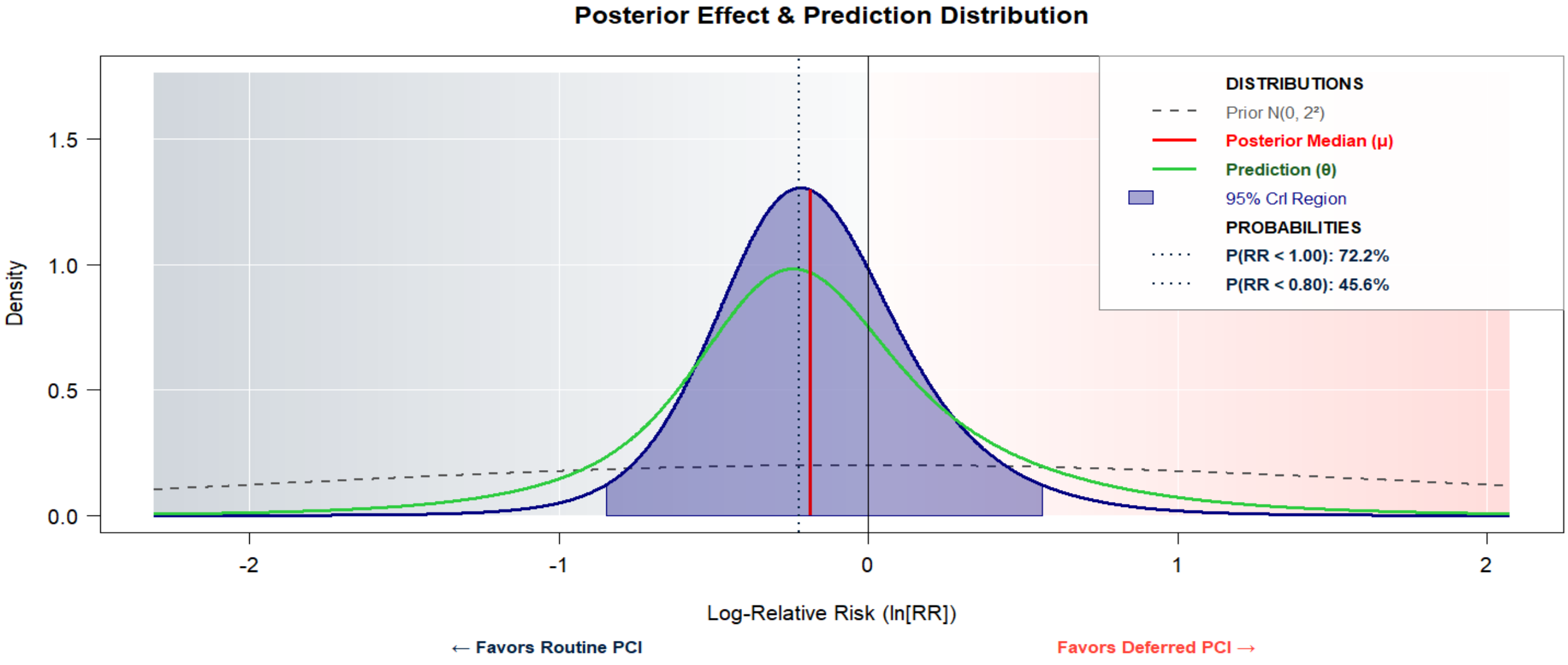

Figure S8. Bayesian Random-Effects Meta-Analysis & Posterior Shrinkage Plot: Acute Kidney Injury (AKI)

Study	Routine PCI (n/N)	Deferred PCI (n/N)	RR [95% CrI]	Weight
NOTION-3	12/227	26/228	0.60 [0.31, 1.15]	51.5%
ACTIVATION	12/119	5/116	1.50 [0.59, 3.99]	35.4%
PRO-TAVI	3/233	1/233	1.20 [0.37, 5.32]	13.1%
**Pooled Effect (μ)**			1.02 [0.41, 2.91]	
**Prediction Interval (θ)**			1.00 [0.21, 5.99]	

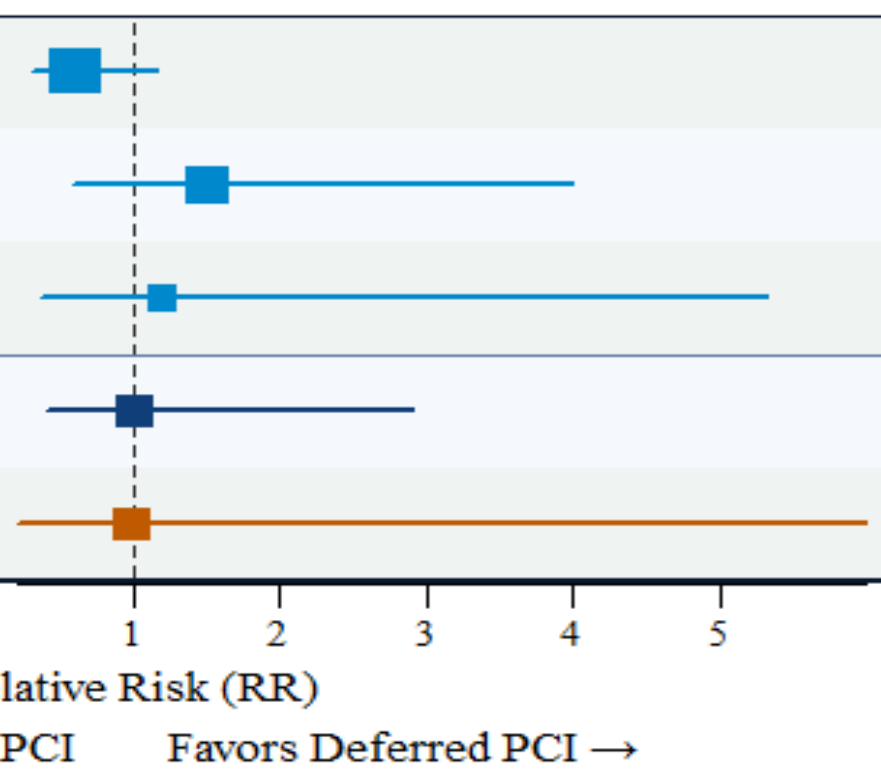

Heterogeneity: τ = 0.56 [95% CrI: 0.00, 1.10]; I² = 49.7%

Figure S9. Bayesian Posterior Probability Density Distribution: Acute Kidney Injury (AKI)

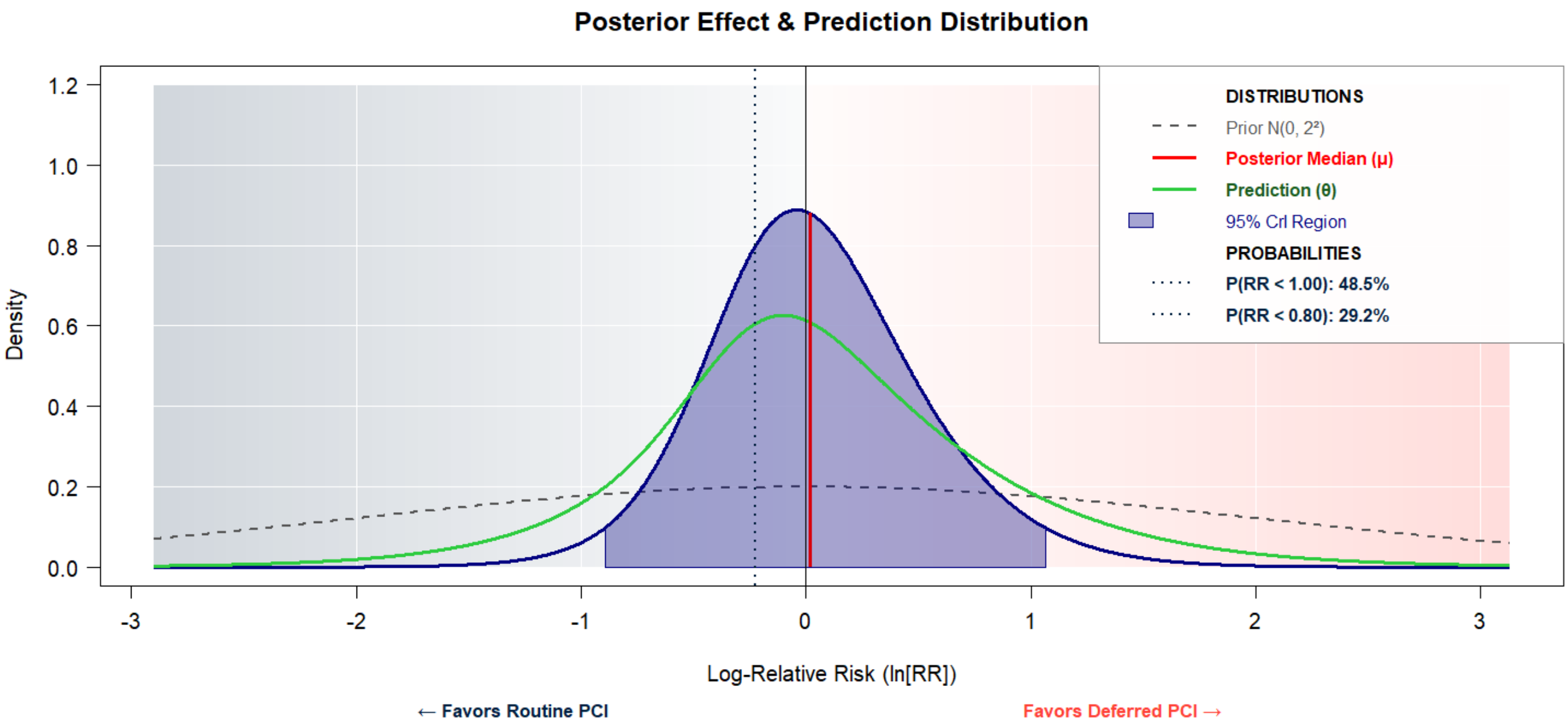

Figure S10. Bayesian Random-Effects Meta-Analysis & Posterior Shrinkage Plot: Stroke (Any Type)

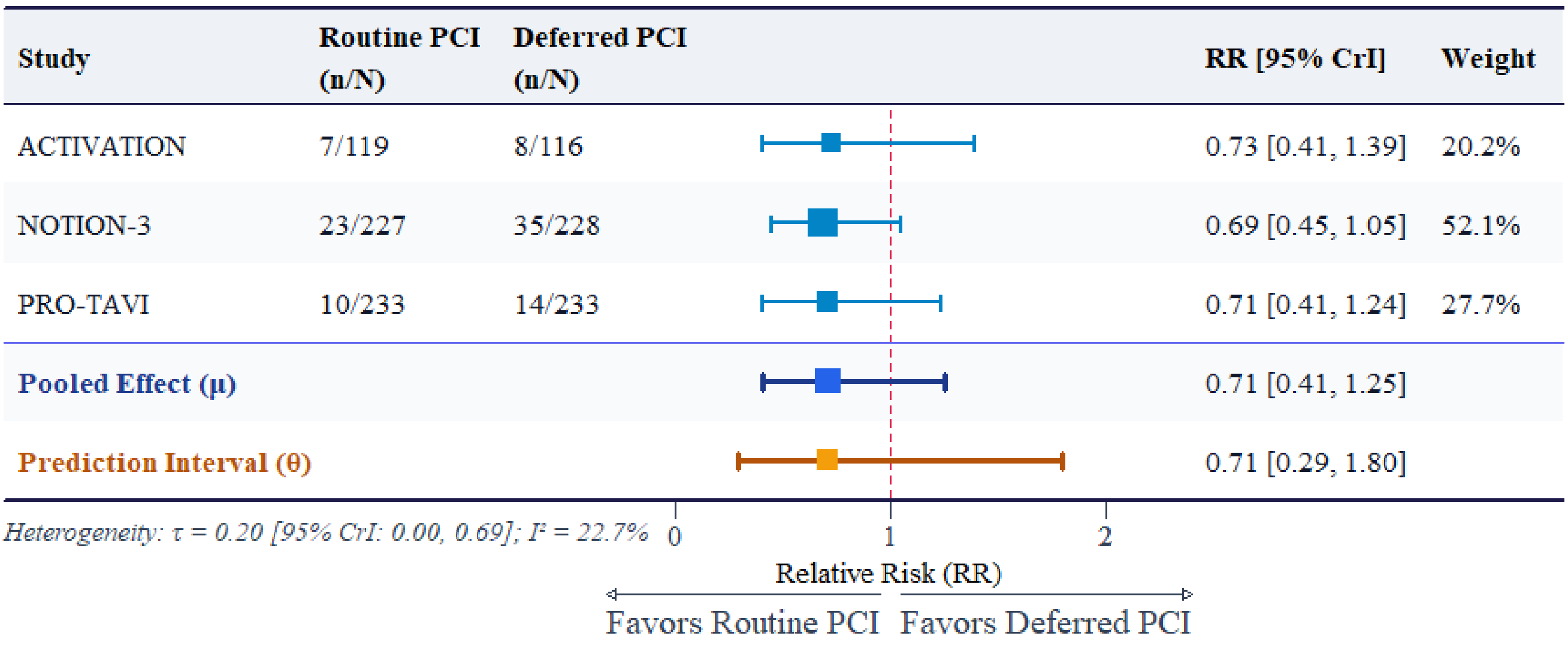

Figure S11. Bayesian Posterior Probability Density Distribution: Stroke (Any Type)

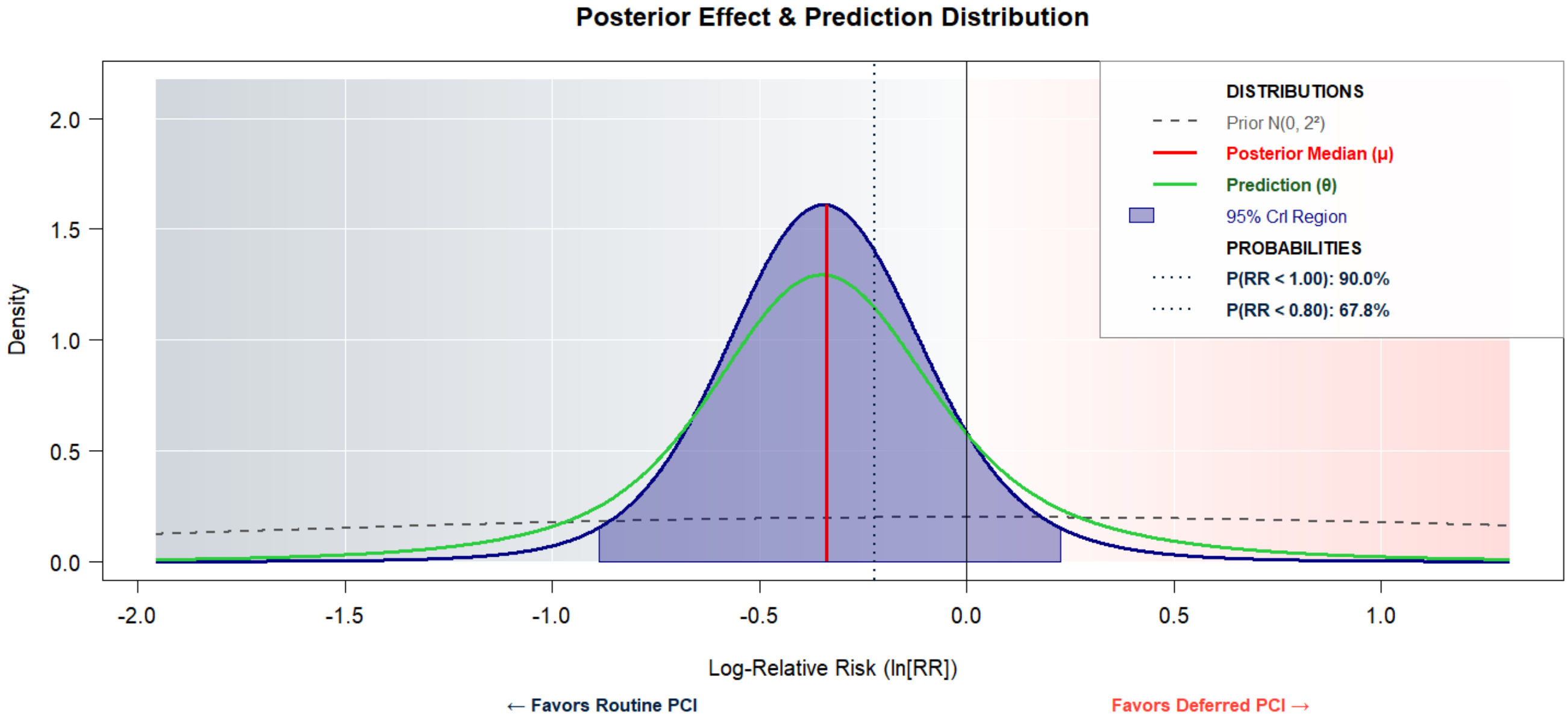

Figure S12. Bayesian Random-Effects Meta-Analysis & Posterior Shrinkage Plot: Any Coronary Revascularization

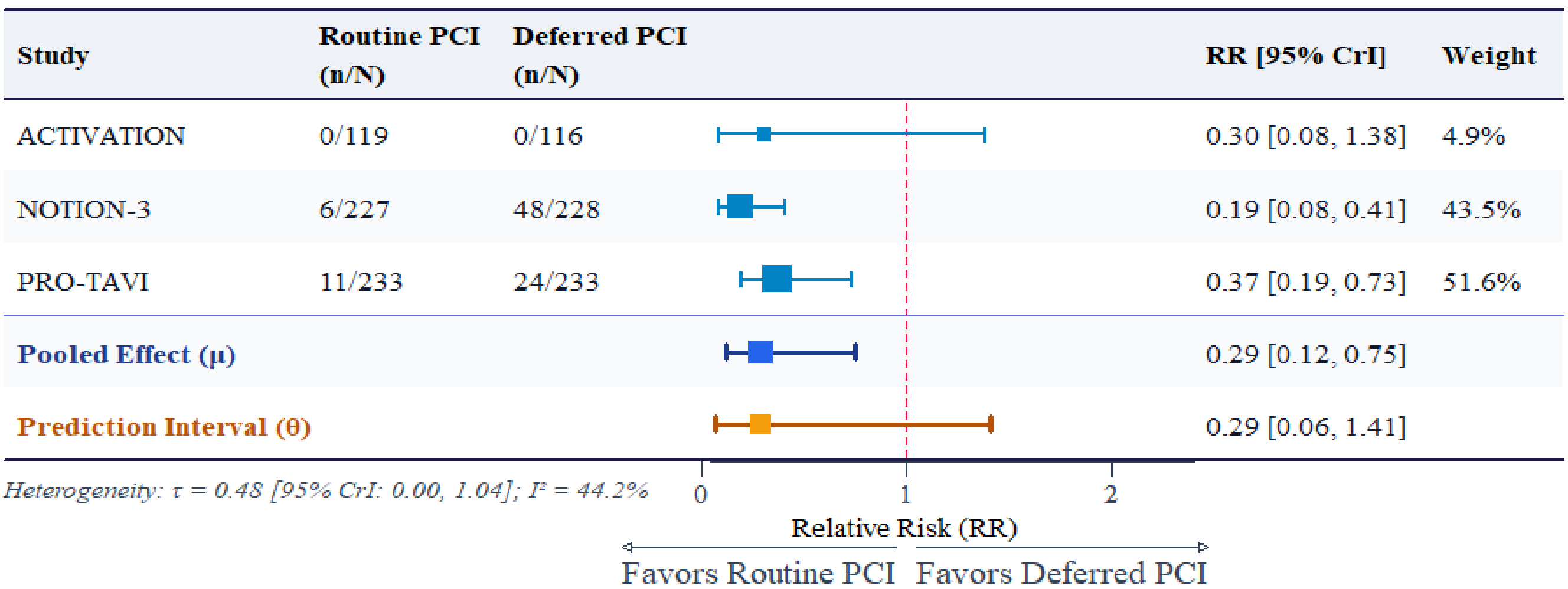

Figure S13. **Bayesian Posterior Probability Density Distribution: Any Coronary Revascularization**

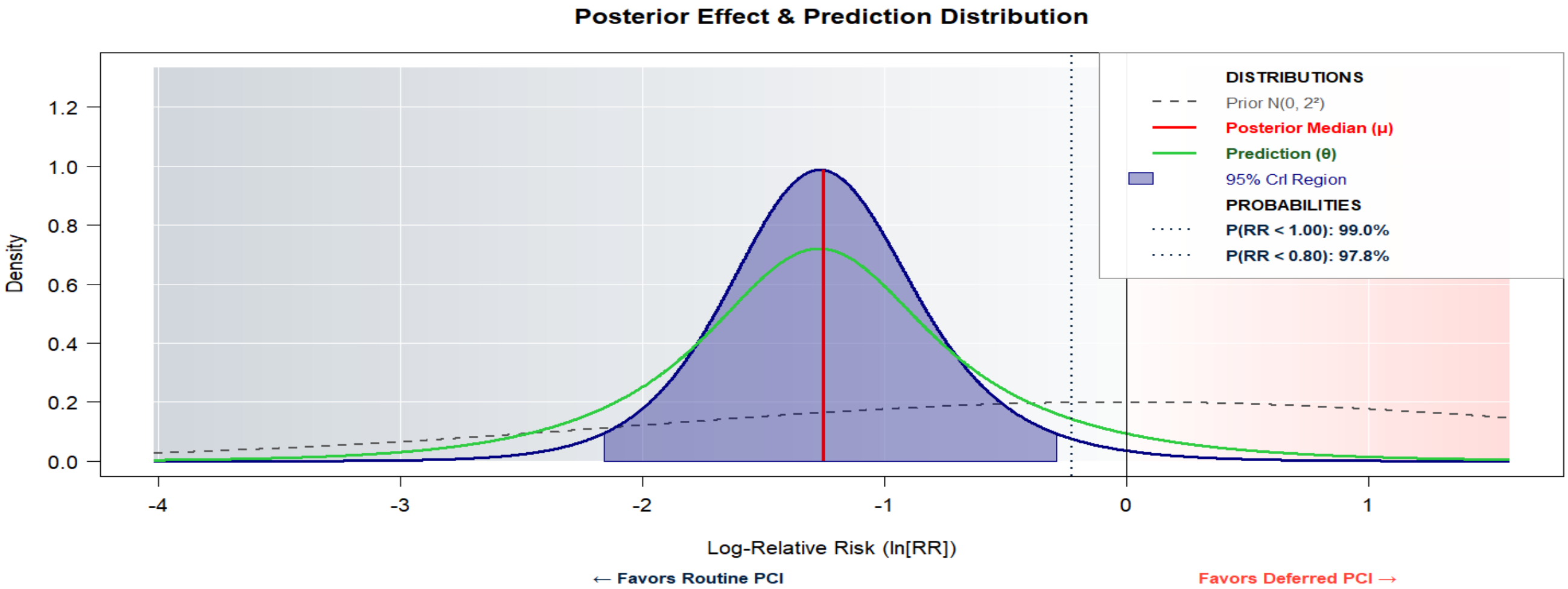

Figure S14. **Bayesian Random-Effects Meta-Analysis & Posterior Shrinkage Plot: Urgent Coronary Revascularization**

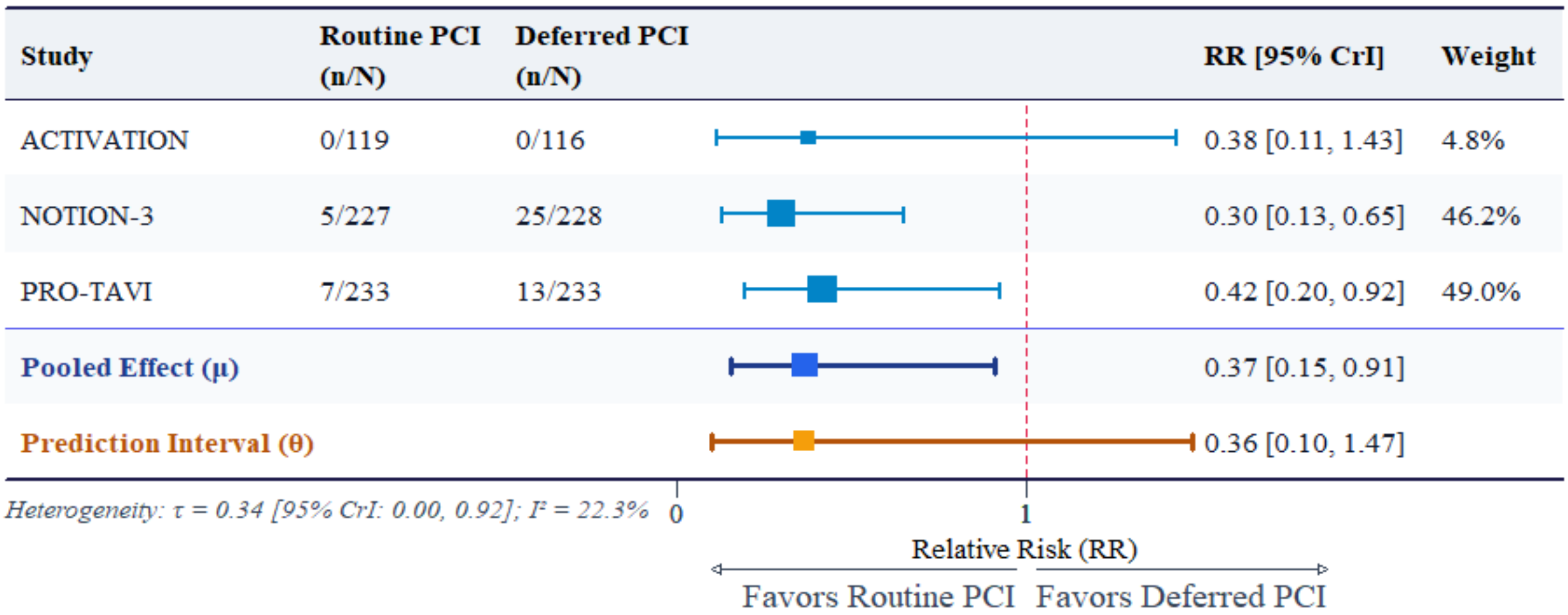

Study	Routine PCI (n/N)	Deferred PCI (n/N)	RR [95% CrI]	Weight
ACTIVATION	0/119	0/116	0.38 [0.11, 1.43]	4.8%
NOTION-3	5/227	25/228	0.30 [0.13, 0.65]	46.2%
PRO-TAVI	7/233	13/233	0.42 [0.20, 0.92]	49.0%
**Pooled Effect (μ)**			0.37 [0.15, 0.91]	
**Prediction Interval (θ)**			0.36 [0.10, 1.47]	

Figure S15. **Bayesian Posterior Probability Density Distribution: Urgent Coronary Revascularization**

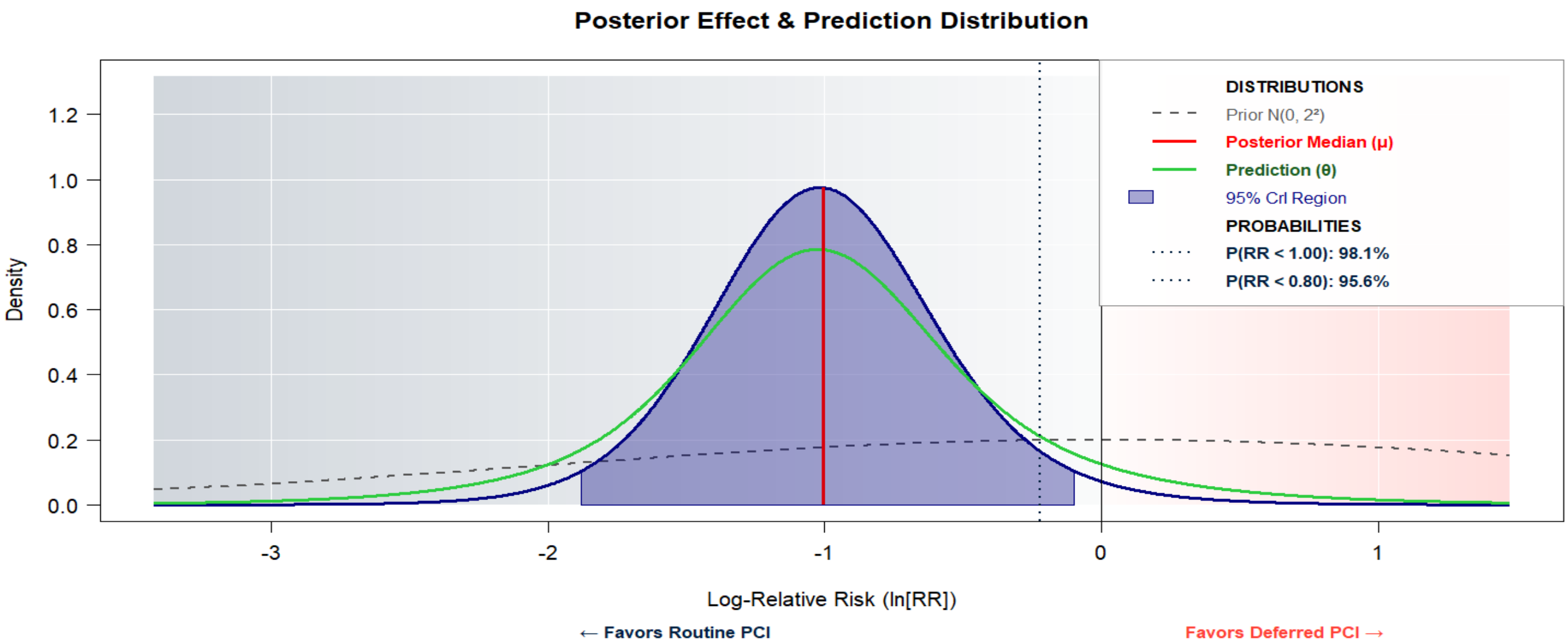

Figure S16. **Bayesian Random-Effects Meta-Analysis & Posterior Shrinkage Plot: Any Bleeding**

Study	Routine PCI (n/N)	Deferred PCI (n/N)	RR [95% CrI]	Weight
ACTIVATION	53/119	33/116	1.65 [1.19, 2.22]	32.2%
NOTION-3	64/227	45/228	1.55 [1.12, 2.09]	33.4%
PRO-TAVI	98/233	40/233	2.19 [1.60, 3.04]	34.4%
**Pooled Effect (μ)**			1.76 [1.06, 2.88]	
**Prediction Interval (θ)**			1.77 [0.66, 4.60]	

Heterogeneity: τ = 0.28 [95% CrI: 0.00, 0.74]; I² = 73.3%

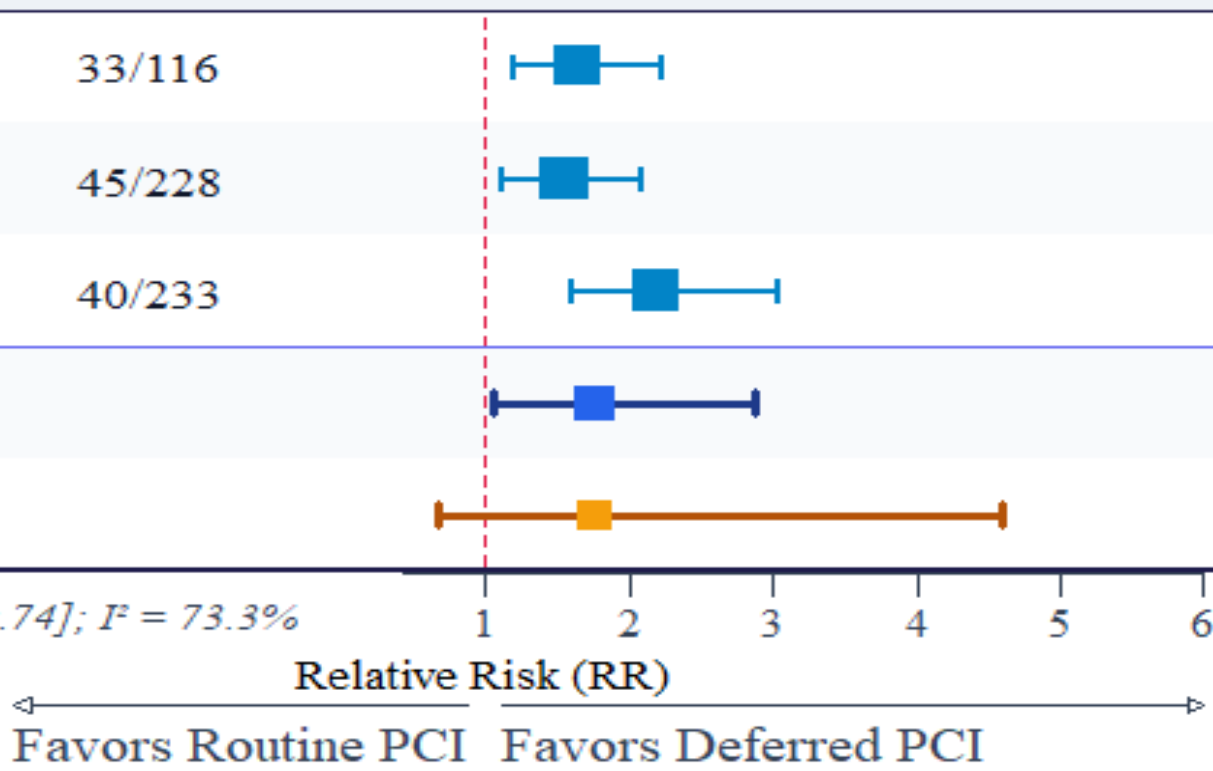

Figure S17. **Bayesian Posterior Probability Density Distribution: Any Bleeding**

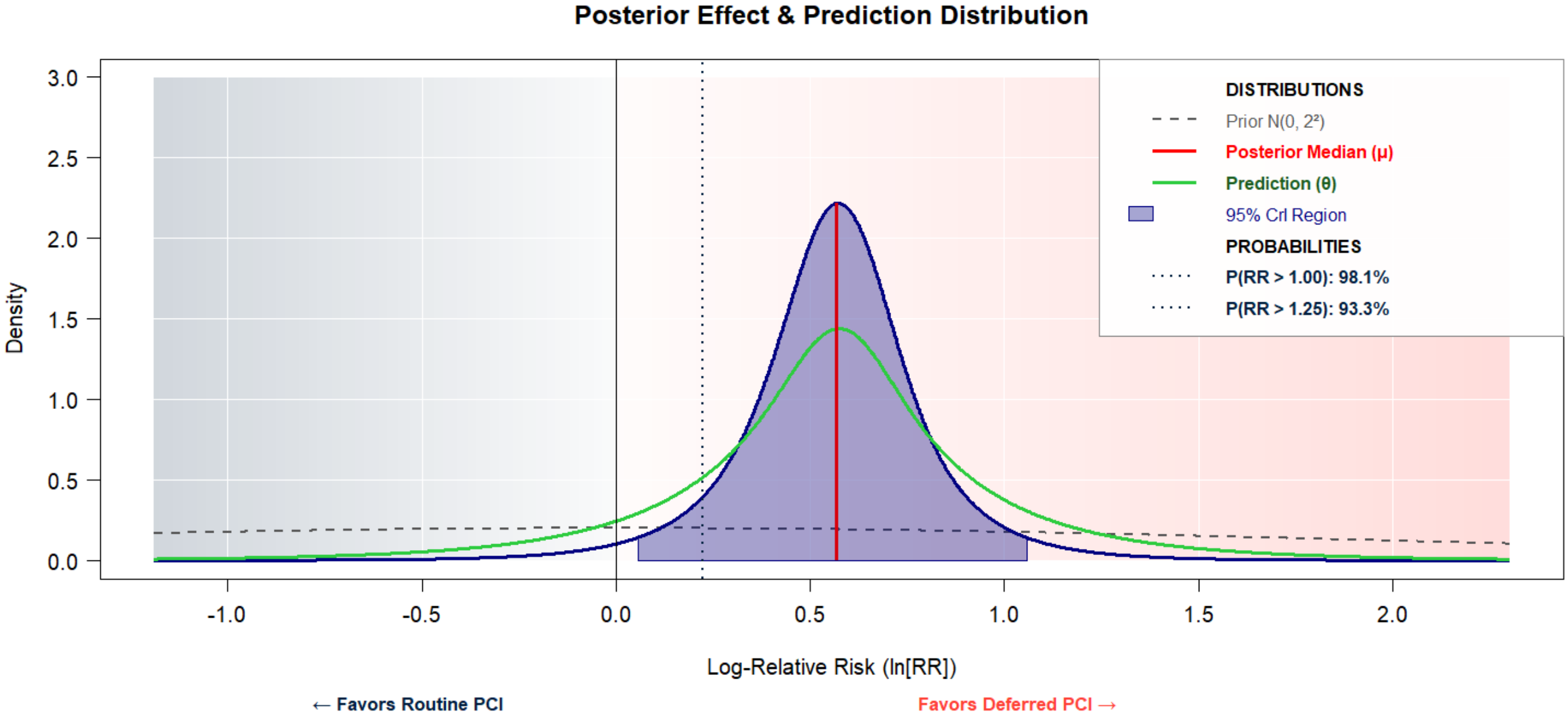

Figure S18. **Bayesian Random-Effects Meta-Analysis & Posterior Shrinkage Plot: Life-Threatening / Disabling Bleeding**

Study	Routine PCI (n/N)	Deferred PCI (n/N)	RR [95% CrI]	Weight
ACTIVATION	19/119	10/116	1.77 [1.05, 3.03]	32.3%
NOTION-3	23/227	16/228	1.64 [0.99, 2.65]	40.4%
PRO-TAVI	18/233	8/233	1.85 [1.07, 3.38]	27.3%
**Pooled Effect (μ)**			1.74 [1.00, 3.07]	
**Prediction Interval (θ)**			1.74 [0.69, 4.44]	

Heterogeneity: τ = 0.21 [95% CrI: 0.00, 0.70]; I² = 25.8%

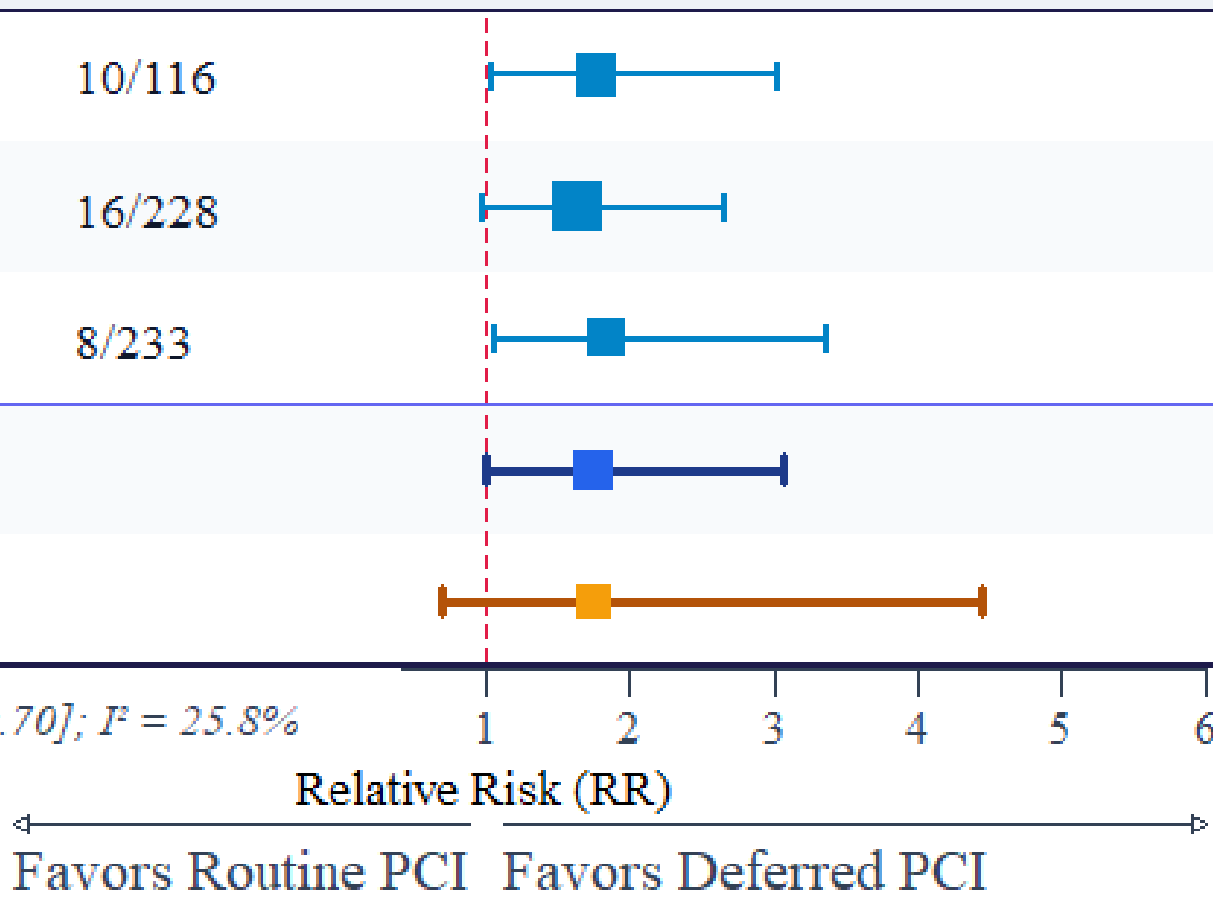

Figure S19. **Bayesian Posterior Probability Density Distribution: Life-Threatening / Disabling Bleeding**

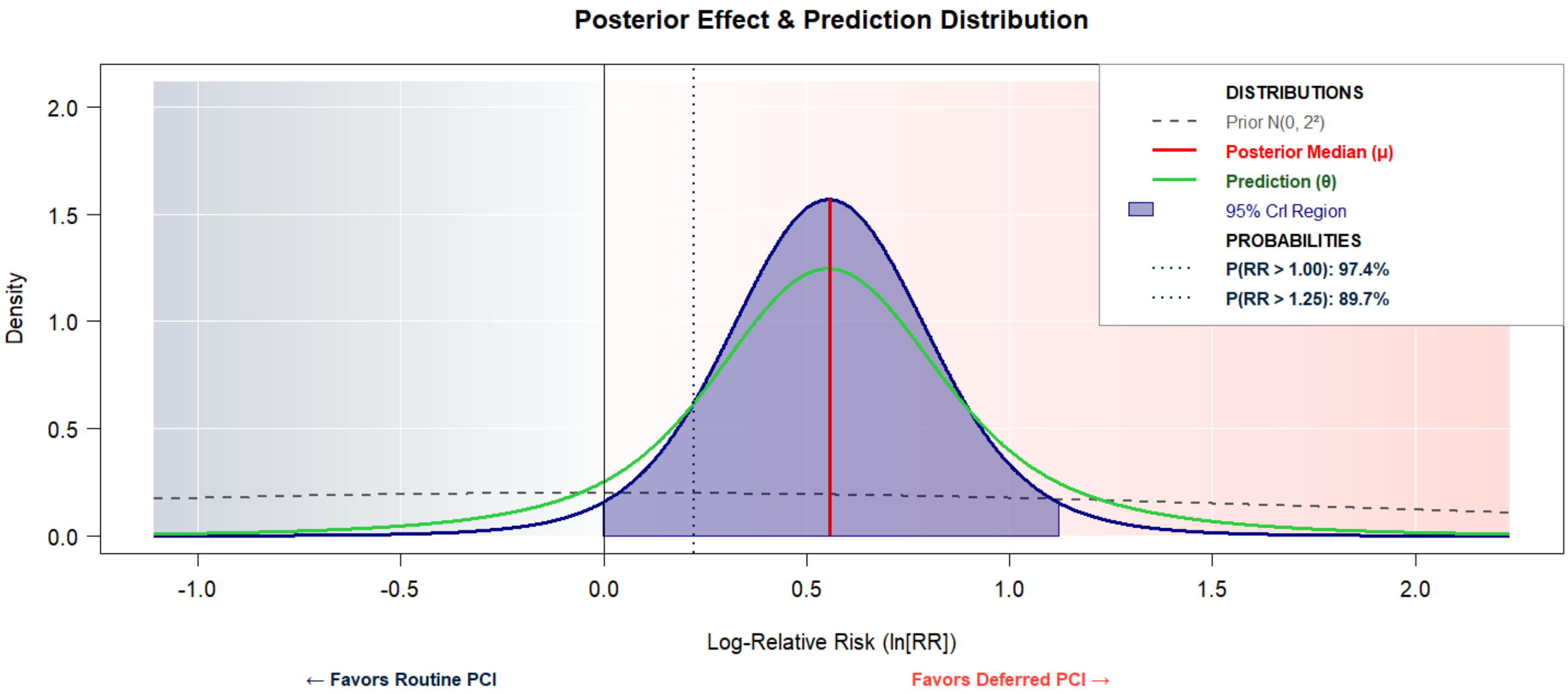

Figure S20. **Bayesian Random-Effects Meta-Analysis & Posterior Shrinkage Plot: Minor Bleeding**

Study	Routine PCI (n/N)	Deferred PCI (n/N)	RR [95% CrI]	Weight
ACTIVATION	15/119	8/116	1.92 [1.04, 3.43]	20.2%
NOTION-3	53/227	36/228	1.64 [1.12, 2.36]	41.0%
PRO-TAVI	73/233	26/233	2.44 [1.65, 3.68]	38.8%
**Pooled Effect (μ)**			1.96 [1.09, 3.48]	
**Prediction Interval (θ)**			1.96 [0.66, 5.72]	

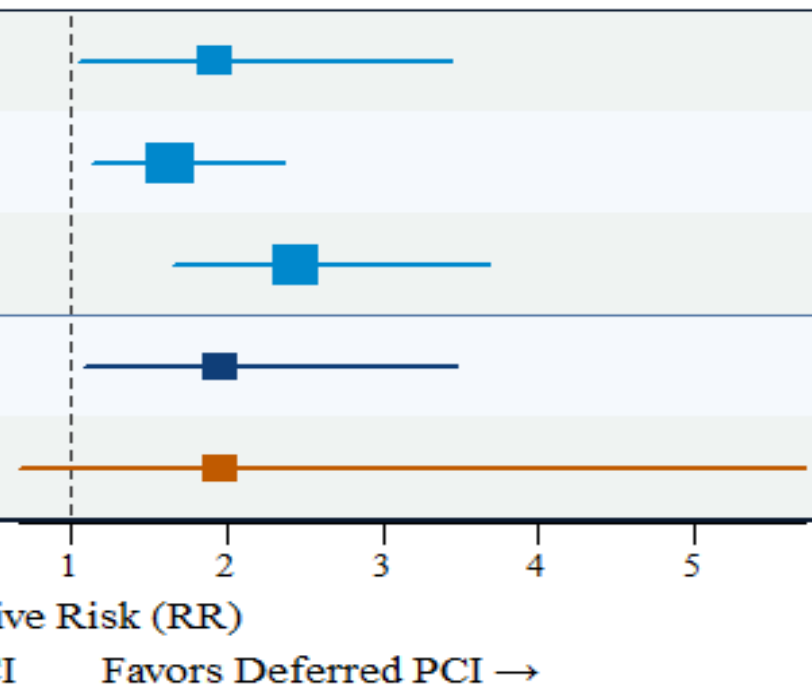

Heterogeneity: τ = 0.32 [95% CrI: 0.00, 0.80]; I² = 62.3%

Figure S21. **Bayesian Posterior Probability Density Distribution: Minor Bleeding**

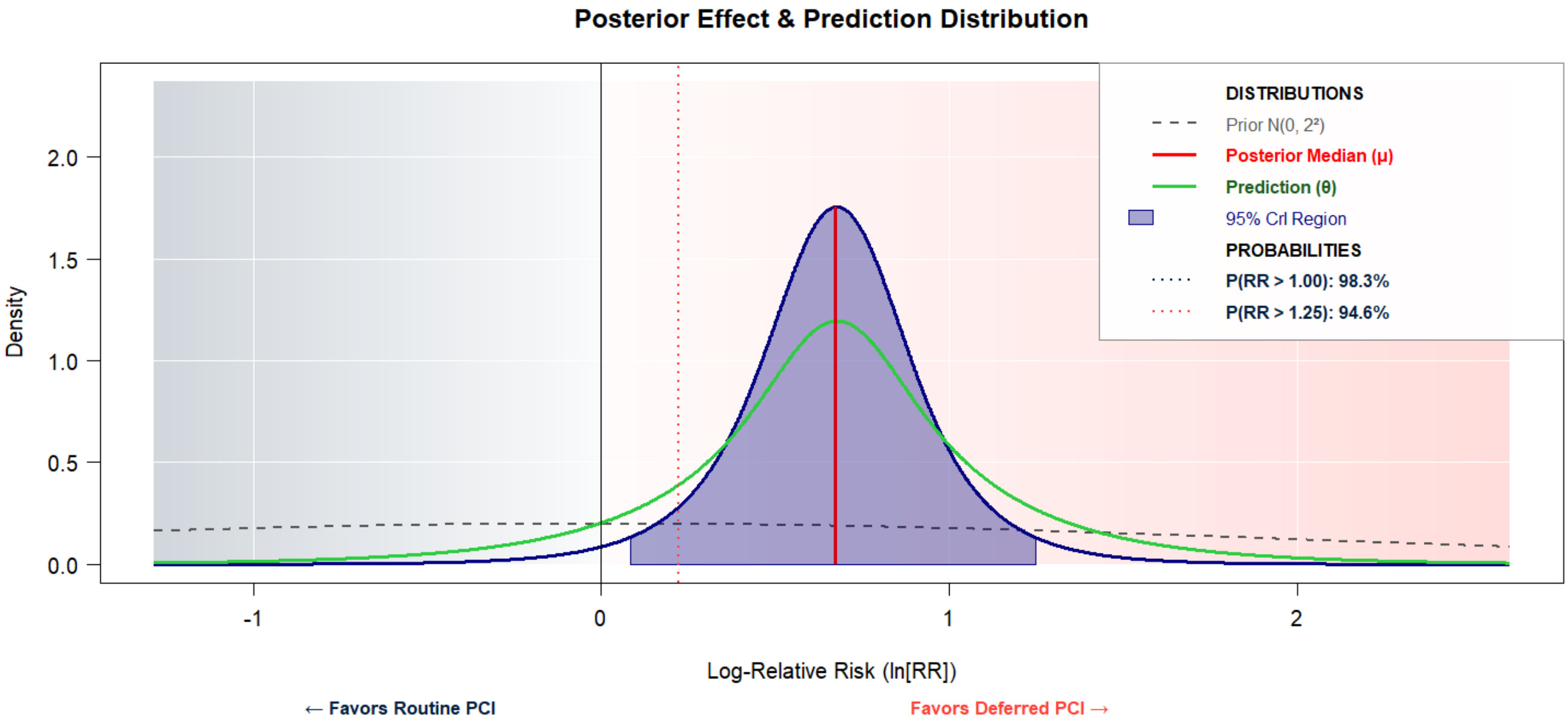

Figure S22. **Bayesian Heterogeneity Prior Sensitivity Analysis: All-Cause Mortality**

Model	RR [95% CrI]	P(RR < 1.0)	P(RR < 0.8)	Heterogeneity
Model 1: Half-Normal (0.5)	0.91 [0.60, 1.43]	69.6%	22.9%	τ = 0.17 (I^2 = 31.7%)
Model 2: Half-Normal (1.0)	0.91 [0.53, 1.65]	67.6%	24.6%	τ = 0.21 (I^2 = 42.1%)
Model 3: Half-Cauchy (0.5)	0.91 [0.59, 1.46]	69.8%	22.9%	τ = 0.16 (I^2 = 29.8%)
Model 4: Half-Cauchy (1.0)	0.91 [0.53, 1.64]	67.9%	24.4%	τ = 0.20 (I^2 = 40.0%)

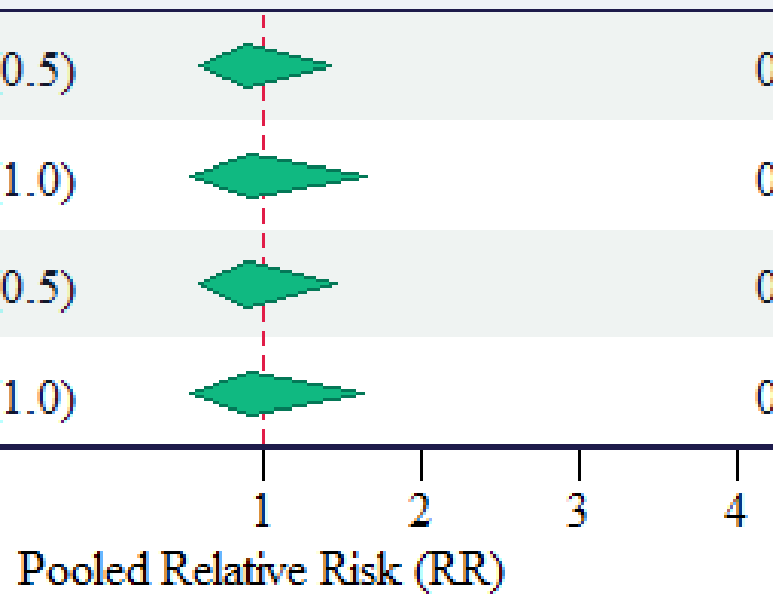

Figure S23. **Bayesian Heterogeneity Prior Sensitivity Analysis: Cardiovascular Death**

Model	RR [95% CrI]	P(RR < 1.0)	P(RR < 0.8)	Heterogeneity
Model 1: Half-Normal (0.5)	0.74 [0.43, 1.29]	87.8%	61.8%	τ = 0.21 (I^2 = 27.0%)
Model 2: Half-Normal (1.0)	0.74 [0.37, 1.51]	84.9%	60.6%	τ = 0.28 (I^2 = 39.6%)
Model 3: Half-Cauchy (0.5)	0.74 [0.42, 1.33]	87.5%	61.7%	τ = 0.20 (I^2 = 25.7%)
Model 4: Half-Cauchy (1.0)	0.74 [0.37, 1.52]	85.1%	60.7%	τ = 0.27 (I^2 = 37.4%)

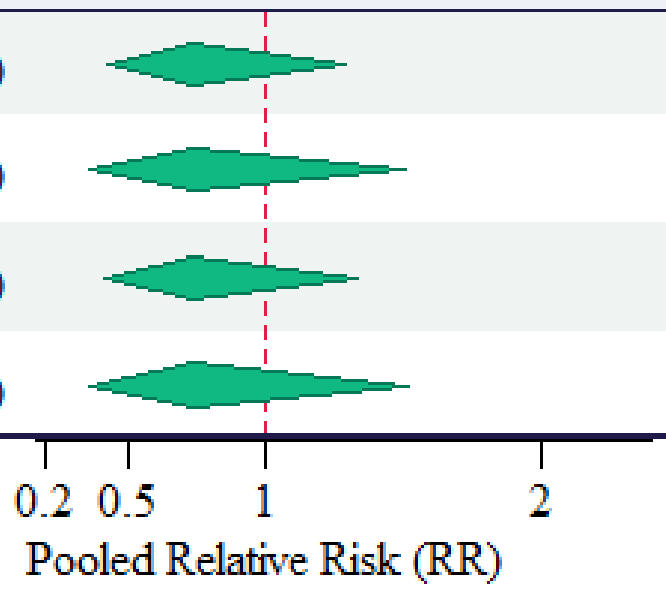

Figure S24. **Bayesian Heterogeneity Prior Sensitivity Analysis: Total Myocardial Infarction**

Model	RR [95% CrI]	P(RR < 1.0)	P(RR < 0.8)	Heterogeneity
Model 1: Half-Normal (0.5)	0.83 [0.43, 1.75]	72.2%	45.6%	τ = 0.34 (I^2 = 41.0%)
Model 2: Half-Normal (1.0)	0.85 [0.36, 2.39]	66.3%	43.2%	τ = 0.50 (I^2 = 60.9%)
Model 3: Half-Cauchy (0.5)	0.83 [0.39, 2.03]	71.0%	45.5%	τ = 0.35 (I^2 = 43.3%)
Model 4: Half-Cauchy (1.0)	0.85 [0.34, 2.56]	66.5%	43.6%	τ = 0.49 (I^2 = 59.7%)

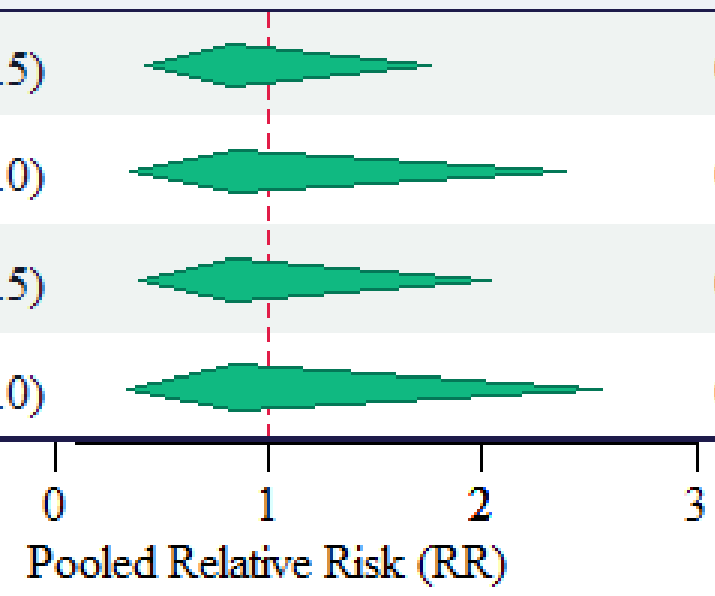

Figure S25. **Bayesian Heterogeneity Prior Sensitivity Analysis: Stroke (Any Type)**

Model	RR [95% CrI]	P(RR < 1.0)	P(RR < 0.8)	Heterogeneity
Model 1: Half-Normal (0.5)	0.71 [0.41, 1.25]	90.0%	67.8%	τ = 0.20 (I^2 = 22.7%)
Model 2: Half-Normal (1.0)	0.72 [0.36, 1.48]	86.8%	65.7%	τ = 0.27 (I^2 = 33.9%)
Model 3: Half-Cauchy (0.5)	0.71 [0.40, 1.29]	89.6%	67.8%	τ = 0.20 (I^2 = 21.5%)
Model 4: Half-Cauchy (1.0)	0.72 [0.36, 1.48]	87.0%	65.9%	τ = 0.26 (I^2 = 31.8%)

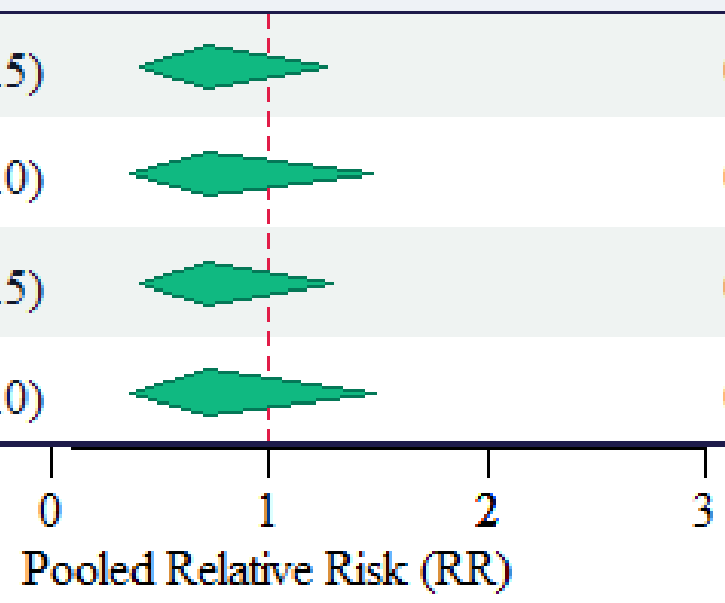

Figure S26. **Bayesian Heterogeneity Prior Sensitivity Analysis: Acute Kidney Injury (AKI)**

Model	RR [95% CrI]	P(RR < 1.0)	P(RR < 0.8)	Heterogeneity
Model 1: Half-Normal (0.5)	1.02 [0.41, 2.91]	48.5%	29.2%	τ = 0.56 (I^2 = 49.7%)
Model 2: Half-Normal (1.0)	1.10 [0.32, 4.42]	43.4%	28.3%	τ = 0.84 (I^2 = 69.5%)
Model 3: Half-Cauchy (0.5)	1.05 [0.33, 4.28]	46.4%	29.6%	τ = 0.70 (I^2 = 61.2%)
Model 4: Half-Cauchy (1.0)	1.10 [0.28, 5.30]	43.7%	29.1%	τ = 0.89 (I^2 = 71.7%)

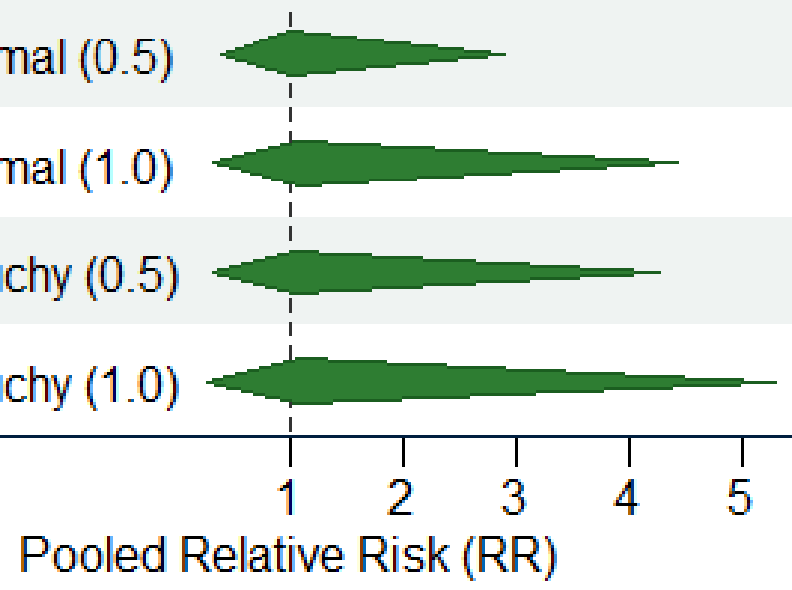

Figure S27. **Bayesian Heterogeneity Prior Sensitivity Analysis: Any Coronary Revascularization**

Model	RR [95% CrI]	P(RR < 1.0)	P(RR < 0.8)	Heterogeneity
Model 1: Half-Normal (0.5)	0.29 [0.12, 0.75]	99.0%	97.8%	τ = 0.48 (I^2 = 44.2%)
Model 2: Half-Normal (1.0)	0.30 [0.09, 1.24]	95.4%	92.6%	τ = 0.75 (I^2 = 66.3%)
Model 3: Half-Cauchy (0.5)	0.30 [0.09, 1.16]	95.9%	93.8%	τ = 0.58 (I^2 = 54.0%)
Model 4: Half-Cauchy (1.0)	0.31 [0.08, 1.60]	93.4%	90.4%	τ = 0.78 (I^2 = 68.2%)

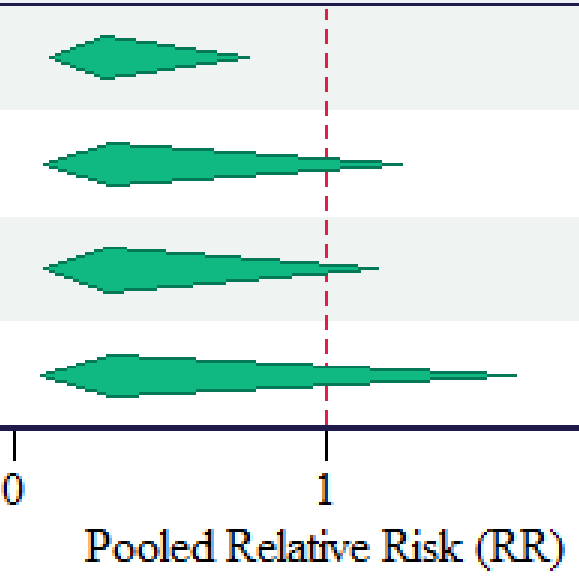

Figure S28. **Bayesian Heterogeneity Prior Sensitivity Analysis: Urgent Coronary Revascularization**

Model	RR [95% CrI]	P(RR < 1.0)	P(RR < 0.8)	Heterogeneity
Model 1: Half-Normal (0.5)	0.37 [0.15, 0.91]	98.1%	95.6%	τ = 0.34 (I^2 = 22.3%)
Model 2: Half-Normal (1.0)	0.38 [0.12, 1.34]	94.3%	90.3%	τ = 0.56 (I^2 = 43.5%)
Model 3: Half-Cauchy (0.5)	0.37 [0.13, 1.14]	95.9%	92.9%	τ = 0.38 (I^2 = 25.9%)
Model 4: Half-Cauchy (1.0)	0.38 [0.11, 1.56]	93.1%	89.1%	τ = 0.56 (I^2 = 43.5%)

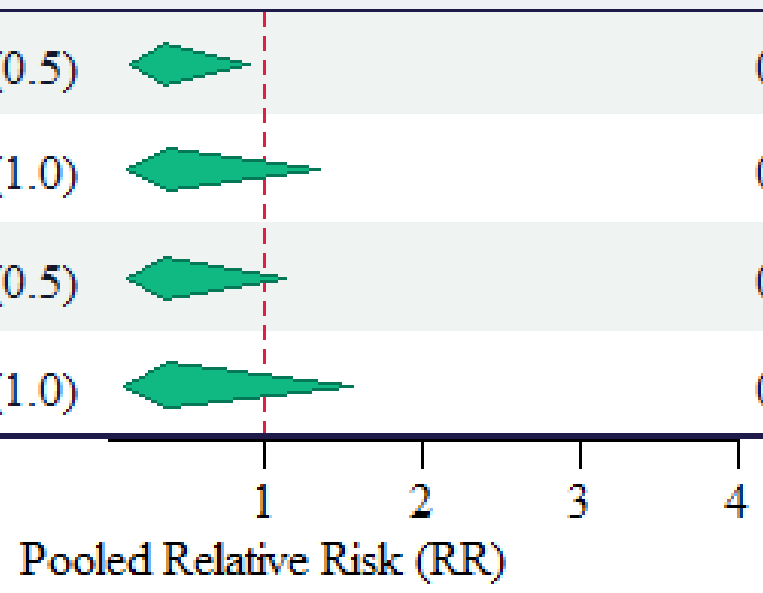

Figure S29. **Bayesian Heterogeneity Prior Sensitivity Analysis: Any Bleeding**

Model	RR [95% CrI]	P(RR > 1.00)	P(RR > 1.25)	Heterogeneity
Model 1: Half-Normal (0.5)	1.76 [1.06, 2.88]	98.1%	93.3%	τ = 0.28 (I^2 = 73.3%)
Model 2: Half-Normal (1.0)	1.76 [0.87, 3.43]	95.6%	89.4%	τ = 0.36 (I^2 = 81.4%)
Model 3: Half-Cauchy (0.5)	1.76 [1.00, 3.03]	97.3%	92.6%	τ = 0.28 (I^2 = 72.9%)
Model 4: Half-Cauchy (1.0)	1.76 [0.85, 3.47]	95.5%	89.6%	τ = 0.34 (I^2 = 80.3%)

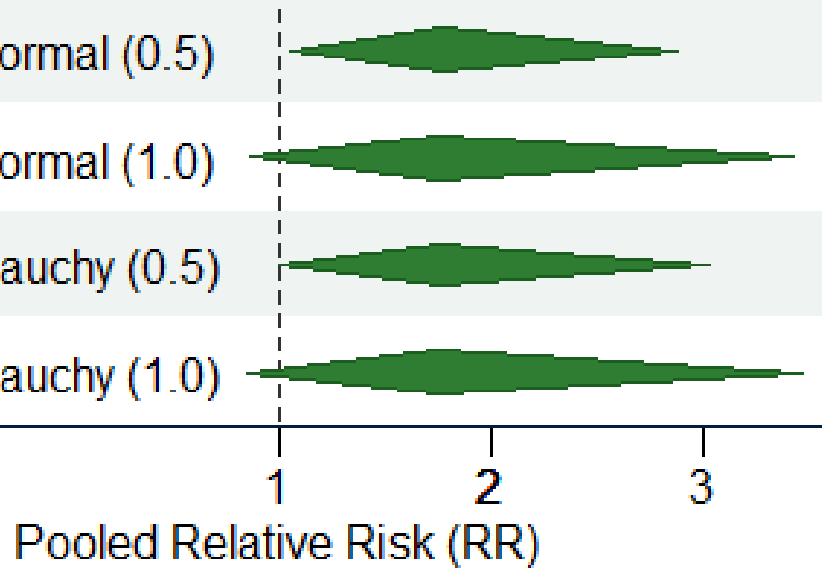

Figure S30. **Bayesian Heterogeneity Prior Sensitivity Analysis: Life-Threatening / Disabling Bleeding**

Model	RR [95% CrI]	P(RR > 1.00)	P(RR > 1.25)	Heterogeneity
Model 1: Half-Normal (0.5)	1.74 [1.00, 3.07]	97.4%	89.7%	$\tau = 0.21$ ($I^2 = 25.8\%$)
Model 2: Half-Normal (1.0)	1.74 [0.86, 3.57]	95.2%	86.8%	$\tau = 0.29$ ($I^2 = 38.3\%$)
Model 3: Half-Cauchy (0.5)	1.74 [0.96, 3.18]	96.9%	89.2%	$\tau = 0.21$ ($I^2 = 24.6\%$)
Model 4: Half-Cauchy (1.0)	1.74 [0.85, 3.59]	95.2%	86.9%	$\tau = 0.27$ ($I^2 = 36.0\%$)

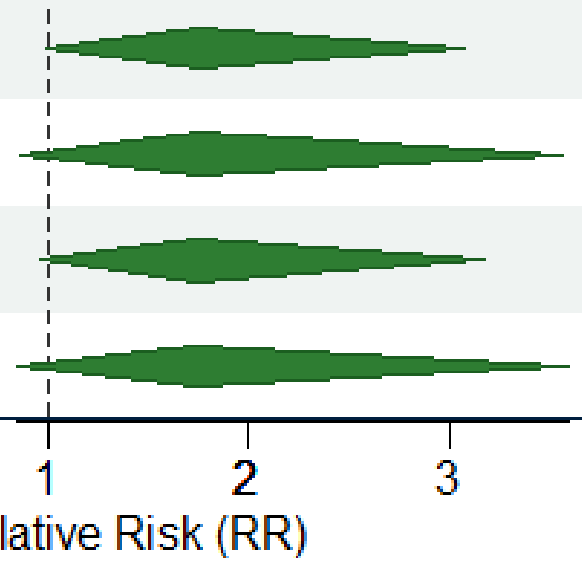

Figure S31. **Bayesian Heterogeneity Prior Sensitivity Analysis: Minor Bleeding**

Model	RR [95% CrI]	P(RR > 1.00)	P(RR > 1.25)	Heterogeneity
Model 1: Half-Normal (0.5)	1.96 [1.09, 3.48]	98.3%	94.6%	$\tau = 0.32$ ($I^2 = 62.3\%$)
Model 2: Half-Normal (1.0)	1.95 [0.86, 4.19]	95.7%	90.6%	$\tau = 0.42$ ($I^2 = 74.0\%$)
Model 3: Half-Cauchy (0.5)	1.96 [1.00, 3.73]	97.3%	93.5%	$\tau = 0.32$ ($I^2 = 62.6\%$)
Model 4: Half-Cauchy (1.0)	1.95 [0.84, 4.30]	95.5%	90.5%	$\tau = 0.41$ ($I^2 = 72.7\%$)

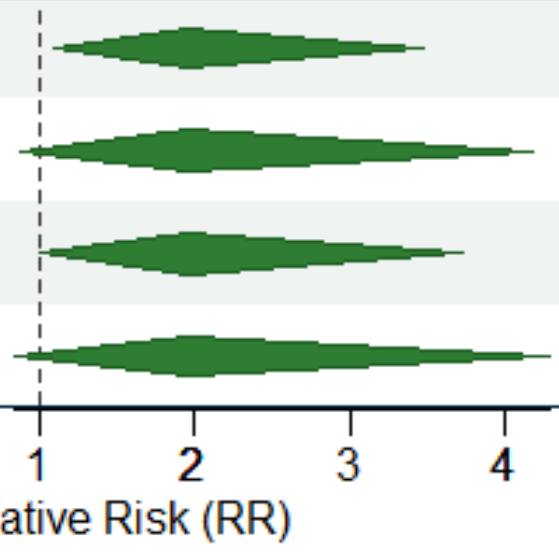

Figure S32. **Frequentist Random-Effects Meta-Analysis (REML vs. HKSJ 95% CIs): All-Cause Mortality**

Study or subgroup	No of events / total: Routine PCI	Deferred PCI	RR	(95% CI)	Weight (%)
ACTIVATION	16 / 119	14 / 116	1.11	[0.57; 2.18]	13.9
NOTION-3	53 / 227	62 / 228	0.86	[0.63; 1.18]	61.9
PRO-TAVI	25 / 233	28 / 233	0.89	[0.54; 1.48]	24.2
Random effects (Standard)	94 / 579	104 / 577	0.90	[0.70; 1.15]	.
Random effects (Hartung-Knapp)			0.90	[0.52; 1.55]	100.0
95% Prediction interval				[0.52; 1.55]	

Heterogeneity: $\tau^2=0$; $\chi^2=0.47$, df=2, P=0.7887; $I^2=0.0\%$
Test for overall effect (Standard): Z = -0.84, P = 0.402
Test for overall effect (Hartung-Knapp): Z = -0.84, P = 0.490

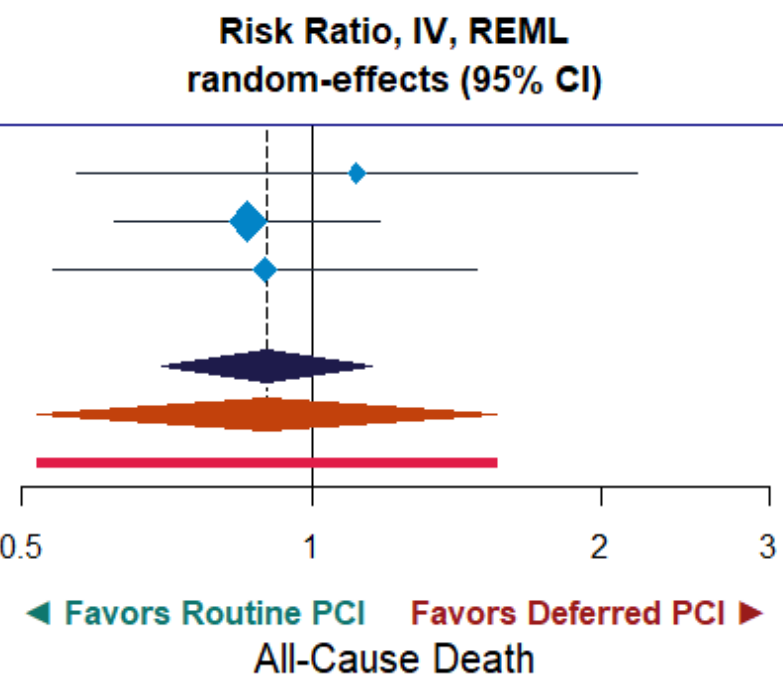

Figure S33. **Frequentist Random-Effects Meta-Analysis (REML vs. HKSJ 95% CIs): Cardiovascular Death**

Study or subgroup	No of events / total: Routine PCI	Deferred PCI	RR	(95% CI)	Weight (%)
ACTIVATION	7 / 119	11 / 116	0.62	[0.25; 1.54]	17.2
NOTION-3	20 / 227	30 / 228	0.67	[0.39; 1.14]	49.9
PRO-TAVI	16 / 233	17 / 233	0.94	[0.49; 1.82]	33.0
Random effects (Standard)	43 / 579	58 / 577	0.74	[0.51; 1.08]	.
Random effects (Hartung-Knapp)			0.74	[0.32; 1.69]	100.0
95% Prediction interval				[0.32; 1.69]	

Heterogeneity: $\tau^2=0$; $\chi^2=0.79$, df=2, P=0.6735; $I^2=0.0\%$
Test for overall effect (Standard): Z = -1.57, P = 0.117
Test for overall effect (Hartung-Knapp): Z = -1.57, P = 0.258

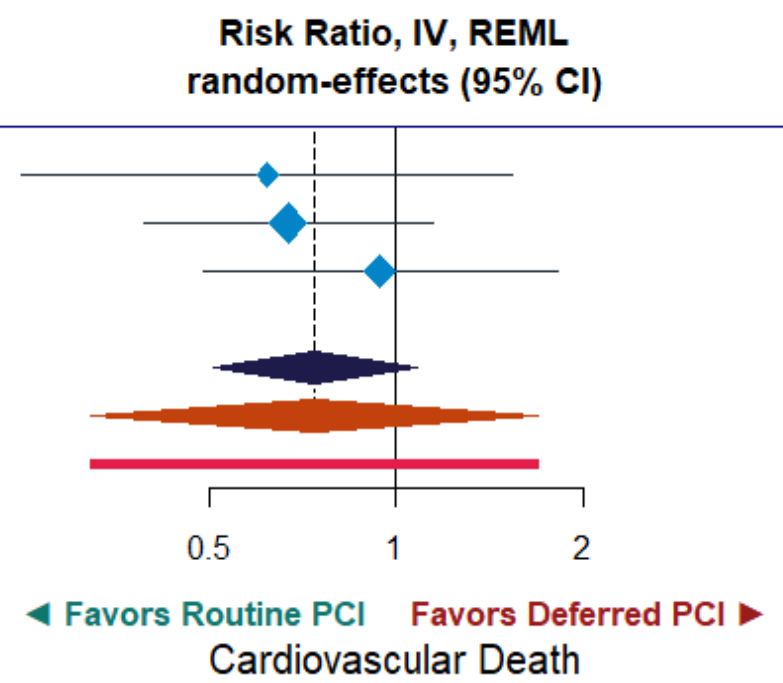

Figure S34. **Frequentist Random-Effects Meta-Analysis (REML vs. HKSJ 95% CIs): Total Myocardial Infarction**

Study or subgroup	No of events / total: Routine PCI	Deferred PCI	RR	(95% CI)	Weight (%)
ACTIVATION	8 / 119	4 / 116	1.95	[0.60; 6.30]	20.8
NOTION-3	17 / 227	31 / 228	0.55	[0.31; 0.97]	44.6
PRO-TAVI	12 / 233	13 / 233	0.92	[0.43; 1.98]	34.6
Random effects (Standard)	37 / 579	48 / 577	0.86	[0.45; 1.63]	.
Random effects (Hartung-Knapp)			0.86	[0.20; 3.68]	100.0
95% Prediction interval				[0.09; 7.92]	

Heterogeneity: τ^2=0.16; χ^2=3.99, df=2, P=0.1362; I^2=49.8%
Test for overall effect (Standard): Z = -1.27, P = 0.638
Test for overall effect (Hartung-Knapp): Z = -0.47, P = 0.693

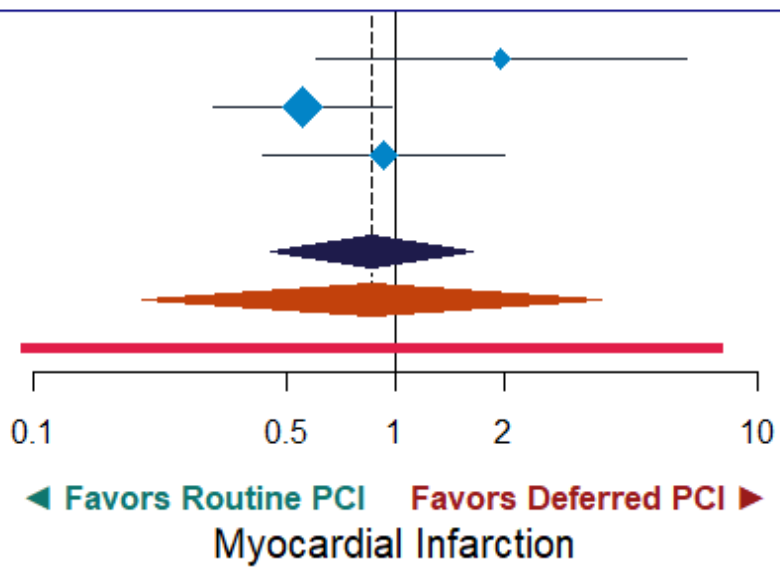

Figure S35. **Frequentist Random-Effects Meta-Analysis (REML vs. HKSJ 95% CIs): Stroke (Any Type)**

Study or subgroup	No of events / total: Routine PCI	Deferred PCI	RR	(95% CI)	Weight (%)
ACTIVATION	7 / 119	8 / 116	0.85	[0.32; 2.28]	15.4
NOTION-3	23 / 227	35 / 228	0.66	[0.40; 1.08]	61.0
PRO-TAVI	10 / 233	14 / 233	0.71	[0.32; 1.58]	23.7
Random effects (Standard)	40 / 579	57 / 577	0.70	[0.48; 1.03]	.
Random effects (Hartung-Knapp)			0.70	[0.30; 1.63]	100.0
95% Prediction interval				[0.30; 1.63]	

Heterogeneity: τ^2=0; χ^2=0.21, df=2, P=0.8991; I^2=0.0%
Test for overall effect (Standard): Z = -1.82, P = 0.069
Test for overall effect (Hartung-Knapp): Z = -1.82, P = 0.210

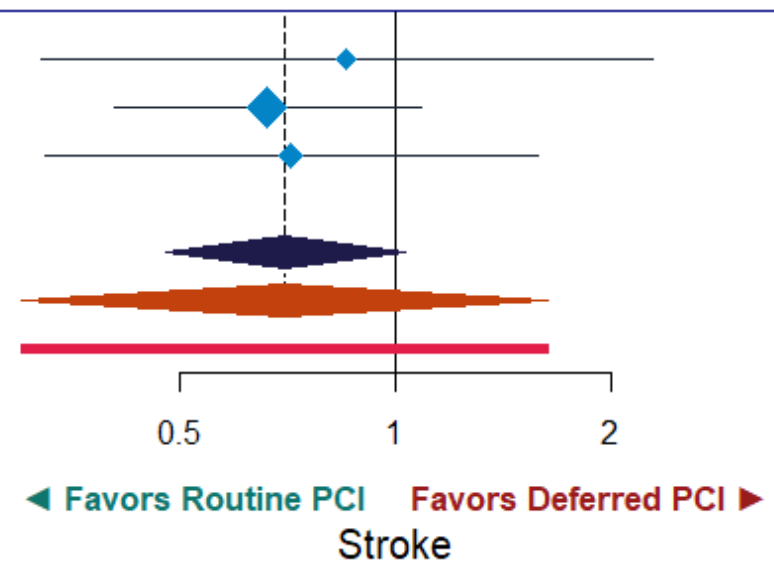

Figure S36. **Frequentist Random-Effects Meta-Analysis (REML vs. HKSJ 95% CIs): Acute Kidney Injury (AKI)**

Study or subgroup	No of events / total: Routine PCI	Deferred PCI	RR	(95% CI)	Weight (%)
ACTIVATION	12 / 119	5 / 116	2.34	[0.85; 6.43]	37.4
NOTION-3	12 / 227	26 / 228	0.46	[0.24; 0.90]	43.4
PRO-TAVI	3 / 233	1 / 233	3.00	[0.31; 28.63]	19.2
Random effects (Standard)	27 / 579	32 / 577	1.22	[0.34; 4.31]	.
Random effects (Hartung-Knapp)			1.22	[0.08; 19.59]	100.0
95% Prediction interval				[0.01; 154.13]	

Heterogeneity: τ^2=0.85; χ^2=8.27, df=2, P=0.0160; I^2=75.8%
Test for overall effect (Standard): Z = -0.76, P = 0.762
Test for overall effect (Hartung-Knapp): Z = 0.30, P = 0.791

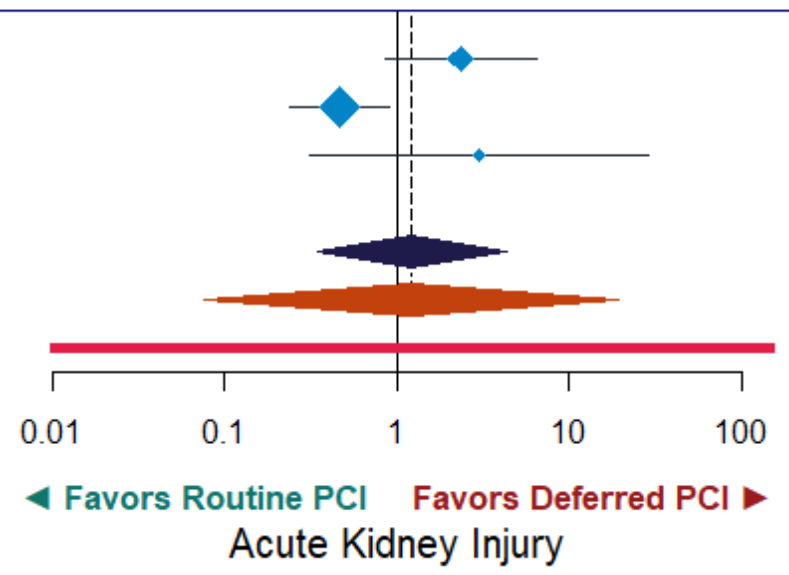

Figure S37. **Frequentist Random-Effects Meta-Analysis (REML vs. HKSJ 95% CIs): Any Coronary Revascularization**

Study or subgroup	No of events / total: Routine PCI	Deferred PCI	RR	(95% CI)	Weight (%)
ACTIVATION	0 / 119	0 / 116	0.97	[0.02; 48.72]	7.0
NOTION-3	6 / 227	48 / 228	0.13	[0.06; 0.30]	44.9
PRO-TAVI	11 / 233	24 / 233	0.47	[0.24; 0.92]	48.1
Random effects (Standard)	17 / 579	72 / 577	0.28	[0.09; 0.85]	.
Random effects (Hartung-Knapp)			0.28	[0.03; 3.17]	100.0
95% Prediction interval				[0.01; 15.07]	

Heterogeneity: τ^2=0.54; χ^2=5.86, df=2, P=0.0533; I^2=65.9%
Test for overall effect (Standard): Z = -4.82, P = 0.024
Test for overall effect (Hartung-Knapp): Z = -2.25, P = 0.153

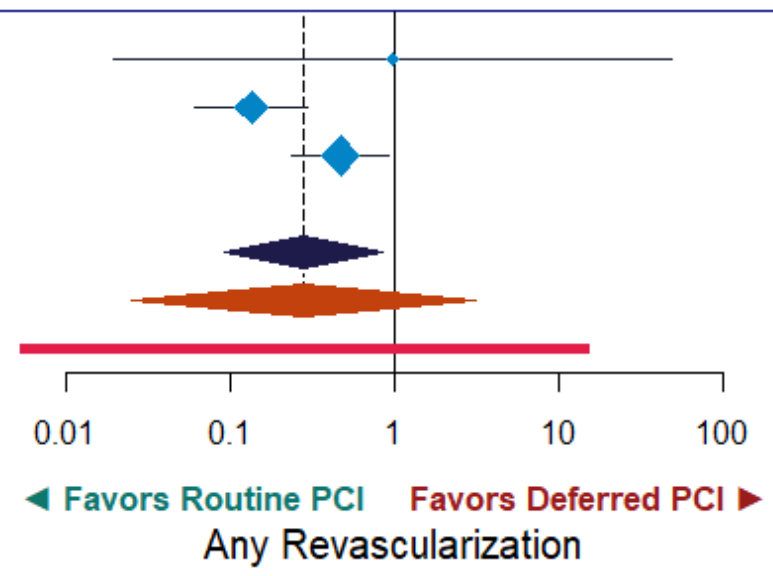

Figure S38. Frequentist Random-Effects Meta-Analysis (REML vs. HKSJ 95% CIs): Urgent Coronary Revascularization

Study or subgroup	No of events / total: Routine PCI	No of events / total: Deferred PCI	RR	(95% CI)	Weight (%)
ACTIVATION	0 / 119	0 / 116	0.97	[0.02; 48.72]	4.6
NOTION-3	5 / 227	25 / 228	0.22	[0.09; 0.53]	46.9
PRO-TAVI	7 / 233	13 / 233	0.56	[0.23; 1.33]	48.5
Random effects (Standard)	12 / 579	38 / 577	0.37	[0.16; 0.86]	.
Random effects (Hartung-Knapp)			0.37	[0.06; 2.40]	100.0
95% Prediction interval				[0.03; 5.30]	

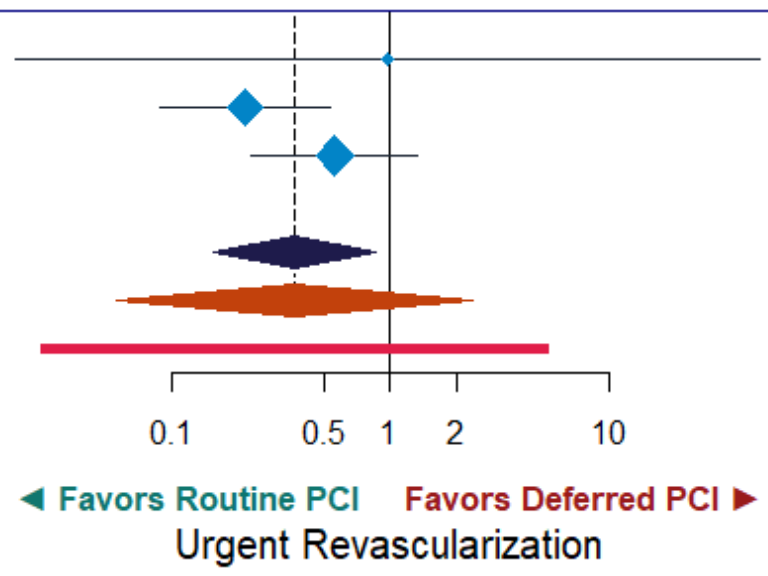

Heterogeneity: τ^2=0.19; χ^2=2.41, df=2, P=0.2995; I^2=17.0%
Test for overall effect (Standard): Z = -3.22, P = 0.022
Test for overall effect (Hartung-Knapp): Z = -2.30, P = 0.148

Figure S39. Frequentist Random-Effects Meta-Analysis (REML vs. HKSJ 95% CIs): Any Bleeding

Study or subgroup	No of events / total: Routine PCI	No of events / total: Deferred PCI	RR	(95% CI)	Weight (%)
ACTIVATION	53 / 119	33 / 116	1.57	[1.10; 2.22]	32.2
NOTION-3	64 / 227	45 / 228	1.43	[1.02; 2.00]	33.4
PRO-TAVI	98 / 233	40 / 233	2.45	[1.78; 3.37]	34.4
Random effects (Standard)	215 / 579	118 / 577	1.77	[1.27; 2.47]	.
Random effects (Hartung-Knapp)			1.77	[0.85; 3.67]	100.0
95% Prediction interval				[0.50; 6.25]	

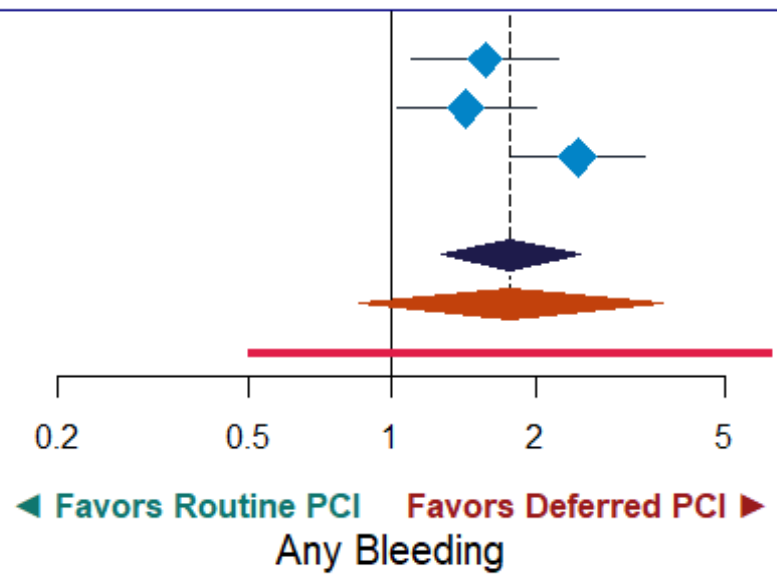

Heterogeneity: τ^2=0.06; χ^2=6.01, df=2, P=0.0495; I^2=66.7%
Test for overall effect (Standard): Z = 5.90, P < 0.001
Test for overall effect (Hartung-Knapp): Z = 3.37, P = 0.078

Figure S40. Frequentist Random-Effects Meta-Analysis (REML vs. HKSJ 95% CIs): Life-Threatening / Disabling Bleeding

Study or subgroup	No of events / total: Routine PCI	No of events / total: Deferred PCI	RR	(95% CI)	Weight (%)
ACTIVATION	19 / 119	10 / 116	1.85	[0.90; 3.81]	31.4
NOTION-3	23 / 227	16 / 228	1.44	[0.78; 2.66]	43.8
PRO-TAVI	18 / 233	8 / 233	2.25	[1.00; 5.07]	24.8
Random effects (Standard)	60 / 579	34 / 577	1.74	[1.16; 2.61]	.
Random effects (Hartung-Knapp)			1.74	[0.72; 4.23]	100.0
95% Prediction interval				[0.72; 4.23]	

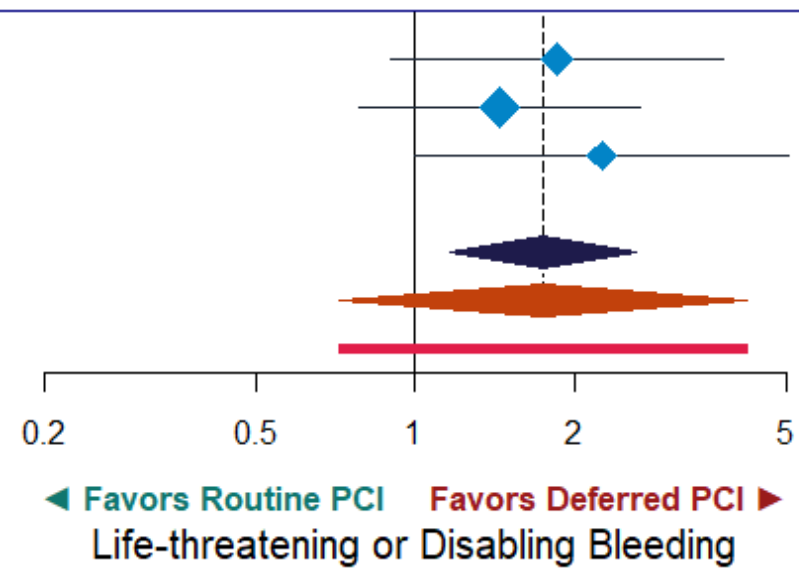

Heterogeneity: τ^2=0; χ^2=0.77, df=2, P=0.6801; I^2=0.0%
Test for overall effect (Standard): Z = 2.69, P = 0.007
Test for overall effect (Hartung-Knapp): Z = 2.69, P = 0.115

Figure S41. Frequentist Random-Effects Meta-Analysis (REML vs. HKSJ 95% CIs): Minor Bleeding

Study or subgroup	No of events / total: Routine PCI	No of events / total: Deferred PCI	RR	(95% CI)	Weight (%)
ACTIVATION	15 / 119	8 / 116	1.83	[0.81; 4.15]	19.8
NOTION-3	53 / 227	36 / 228	1.48	[1.01; 2.17]	41.0
PRO-TAVI	73 / 233	26 / 233	2.81	[1.86; 4.23]	39.2
Random effects (Standard)	141 / 579	70 / 577	1.98	[1.27; 3.10]	.
Random effects (Hartung-Knapp)			1.98	[0.74; 5.30]	100.0
95% Prediction interval				[0.39; 10.01]	

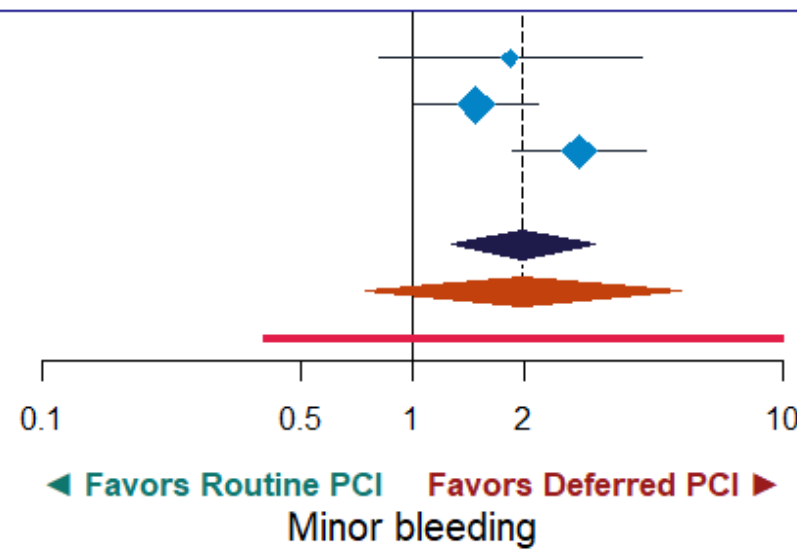

Heterogeneity: τ^2=0.09; χ^2=5.09, df=2, P=0.0786; I^2=60.7%
Test for overall effect (Standard): Z = 5.05, P = 0.003
Test for overall effect (Hartung-Knapp): Z = 3.00, P = 0.096

Figure S42. **Bayesian Random-Effects Meta-Analysis & Posterior Shrinkage Plot: NYHA Class I–II**

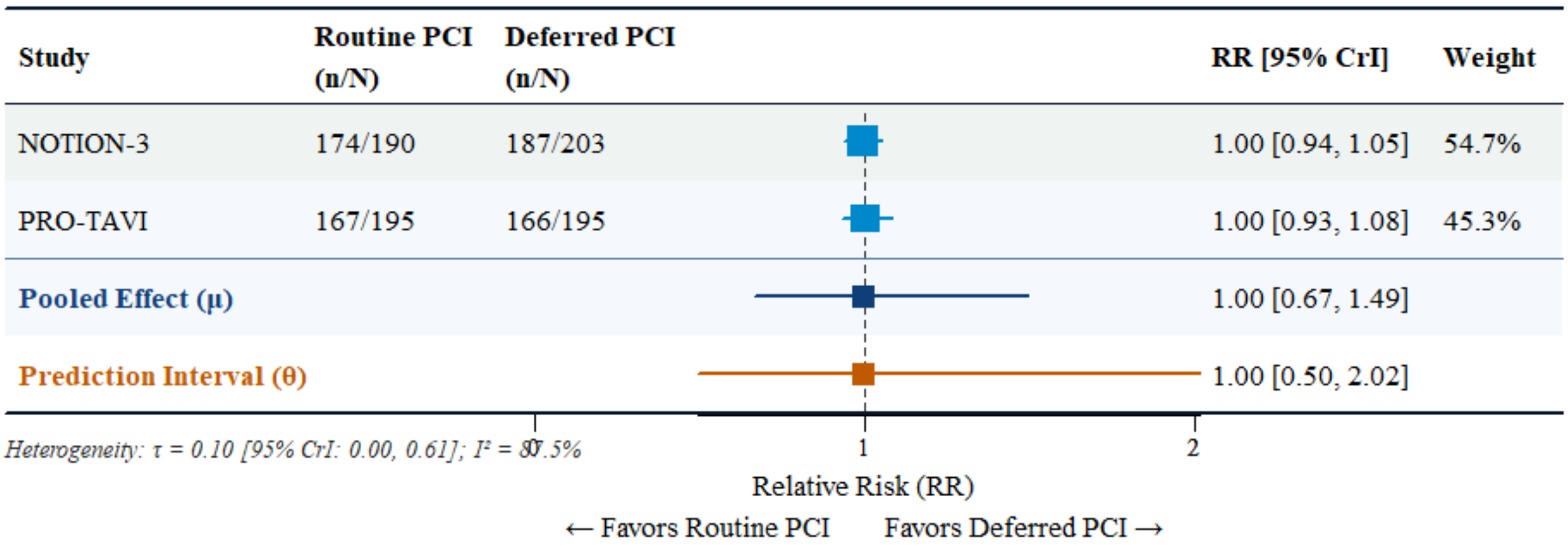

Figure S43. **Frequentist Random-Effects Meta-Analysis (REML vs. HKSJ 95% CIs): NYHA Class I–II**

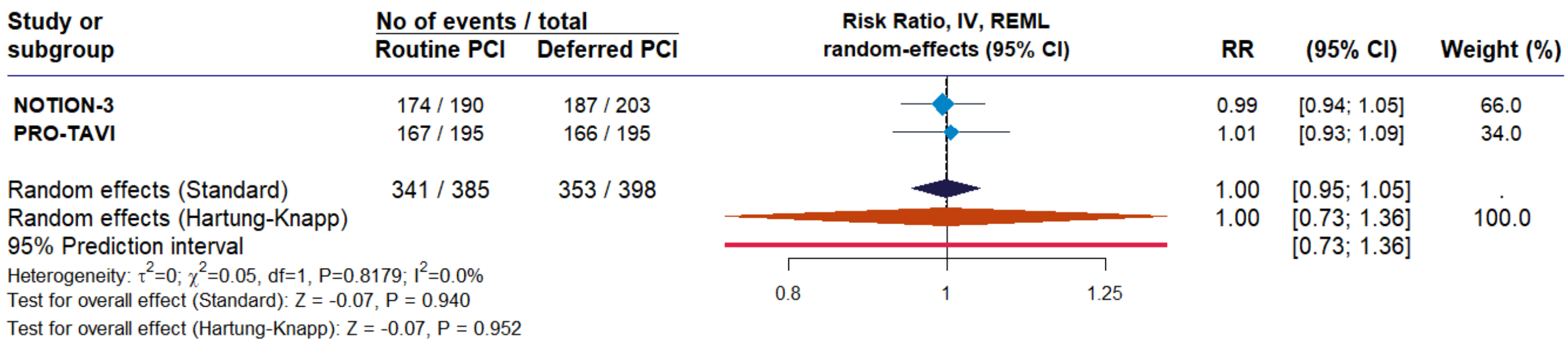

Figure S44. **Bayesian Heterogeneity Prior Sensitivity Analysis: NYHA Class I–II**

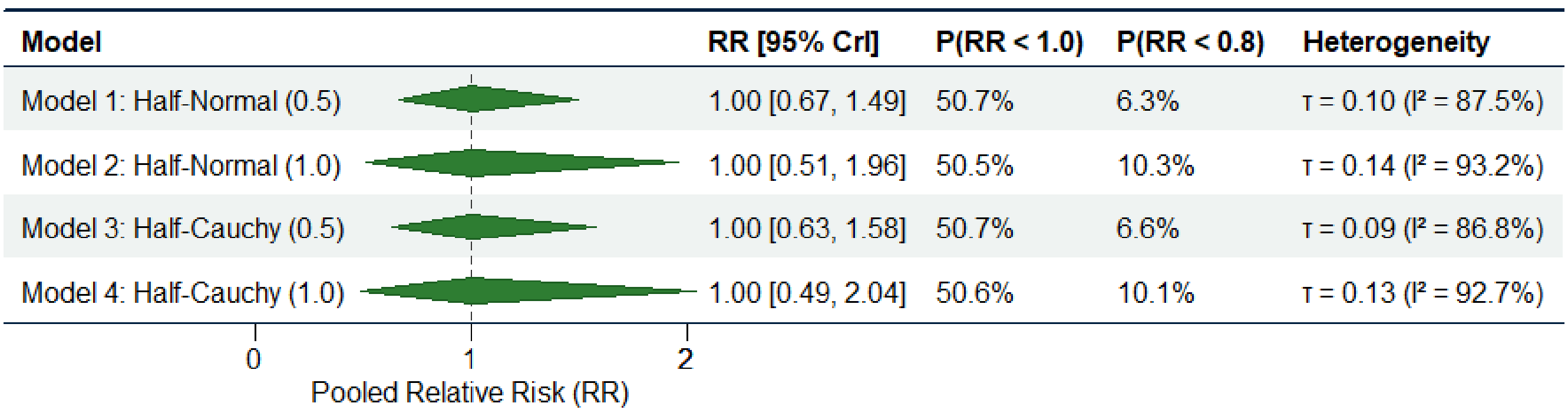

Figure S45. **Bayesian Random-Effects Meta-Analysis & Posterior Shrinkage Plot: NYHA Class III–IV**

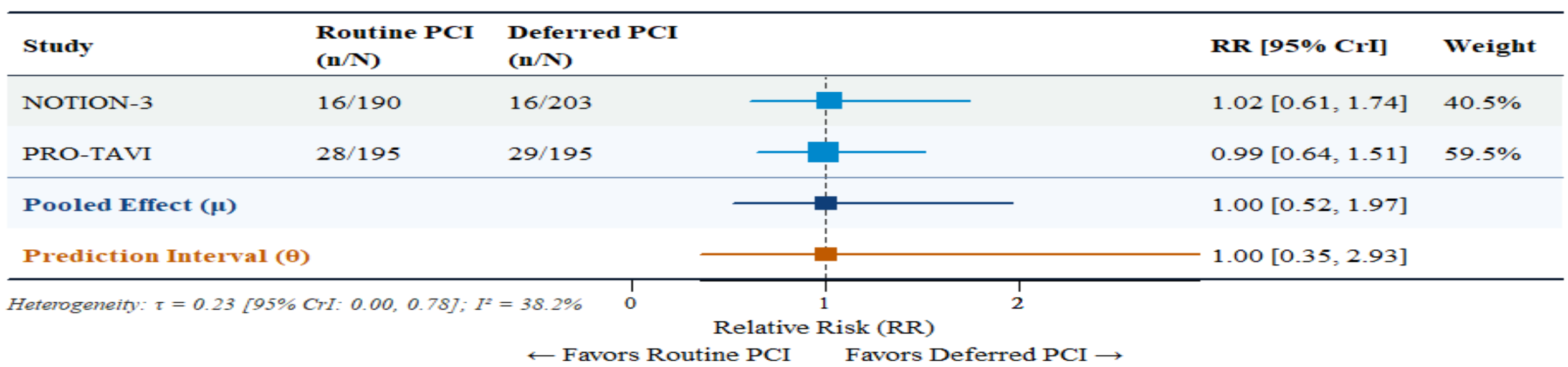

Figure S46. **Frequentist Random-Effects Meta-Analysis (REML vs. HKSJ 95% CIs): NYHA Class III–IV**

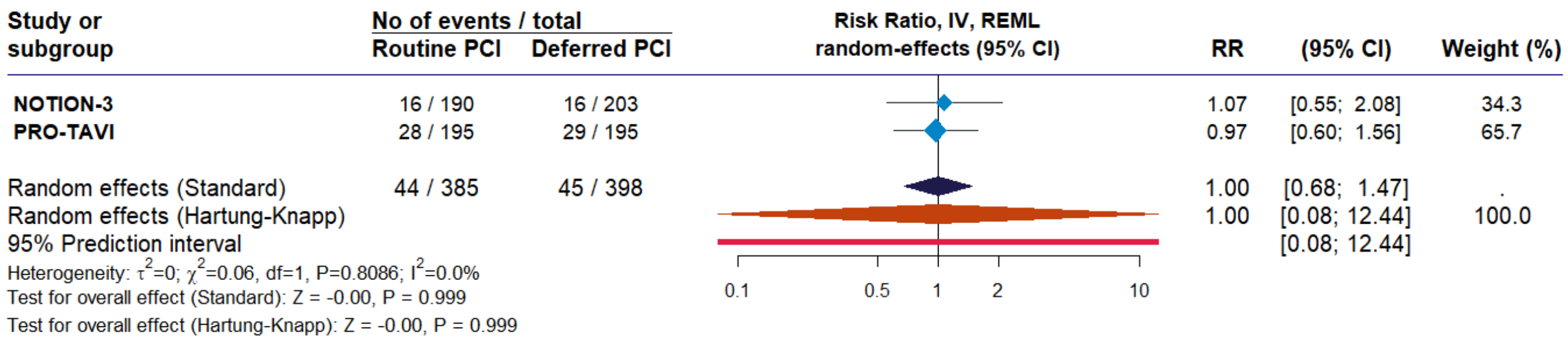

Figure S47. **Bayesian Heterogeneity Prior Sensitivity Analysis: NYHA Class III–IV**

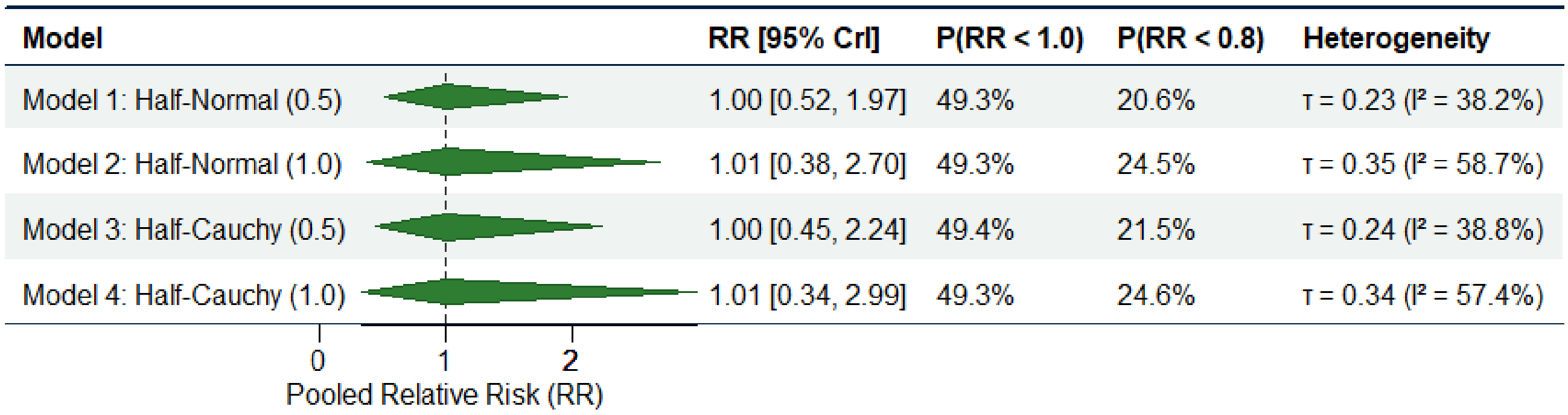

Figure S48. **Bayesian Random-Effects Meta-Analysis & Posterior Shrinkage Plot: CCS Class 0 (No Angina)**

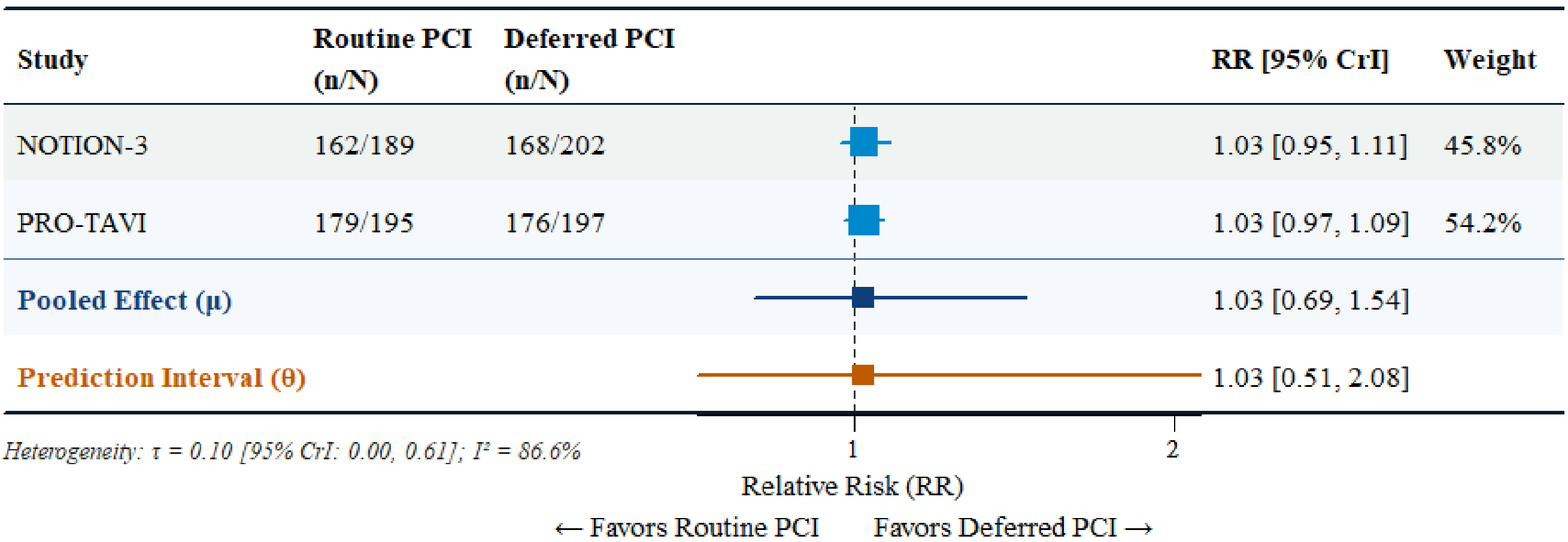

Figure S49. **Frequentist Random-Effects Meta-Analysis (REML vs. HKSJ 95% CIs): CCS Class 0 (No Angina)**

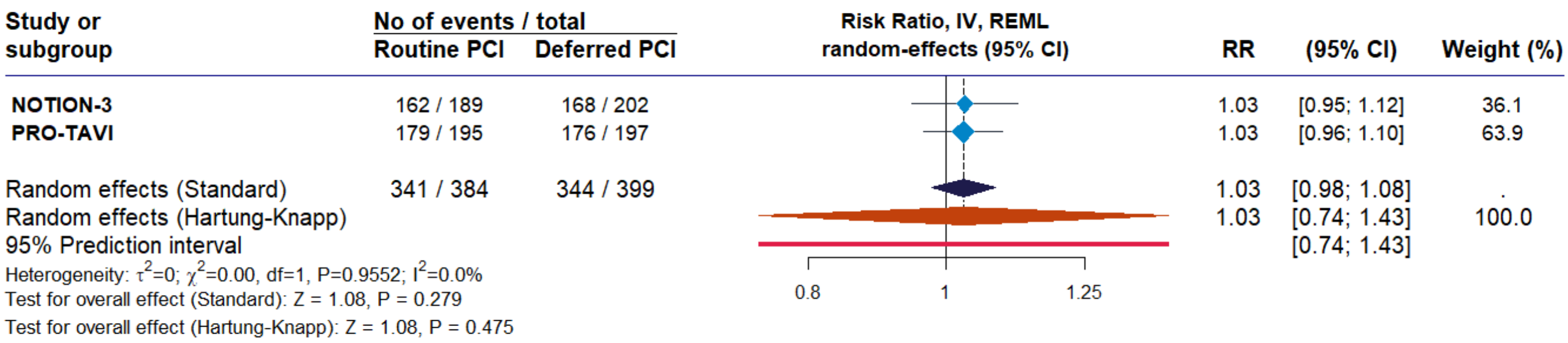

Figure S50. **Bayesian Heterogeneity Prior Sensitivity Analysis: CCS Class 0 (No Angina)**

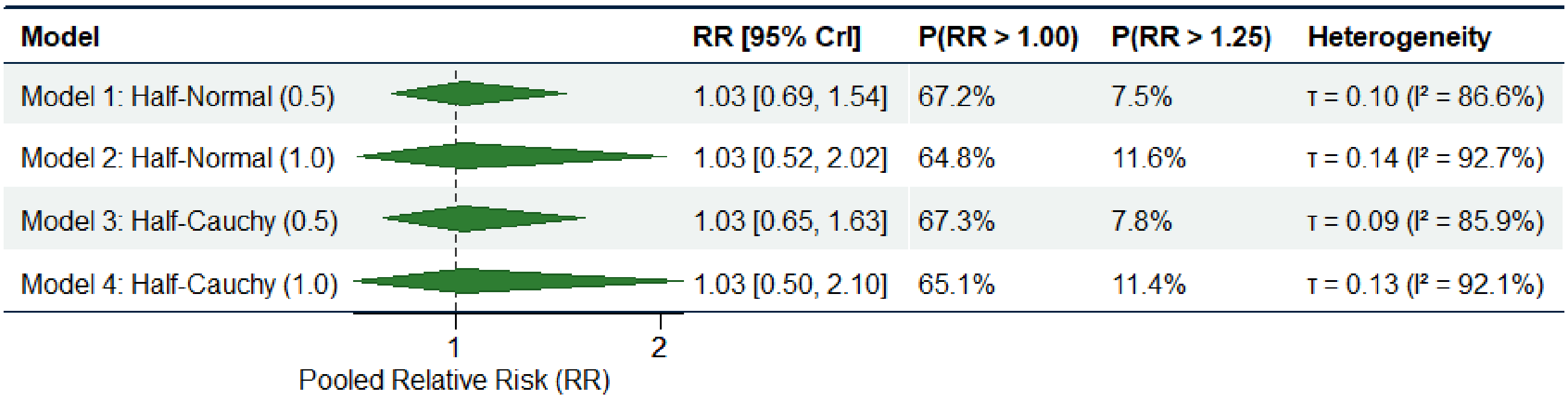

Figure S51. **Bayesian Random-Effects Meta-Analysis & Posterior Shrinkage Plot: CCS Class I–II**

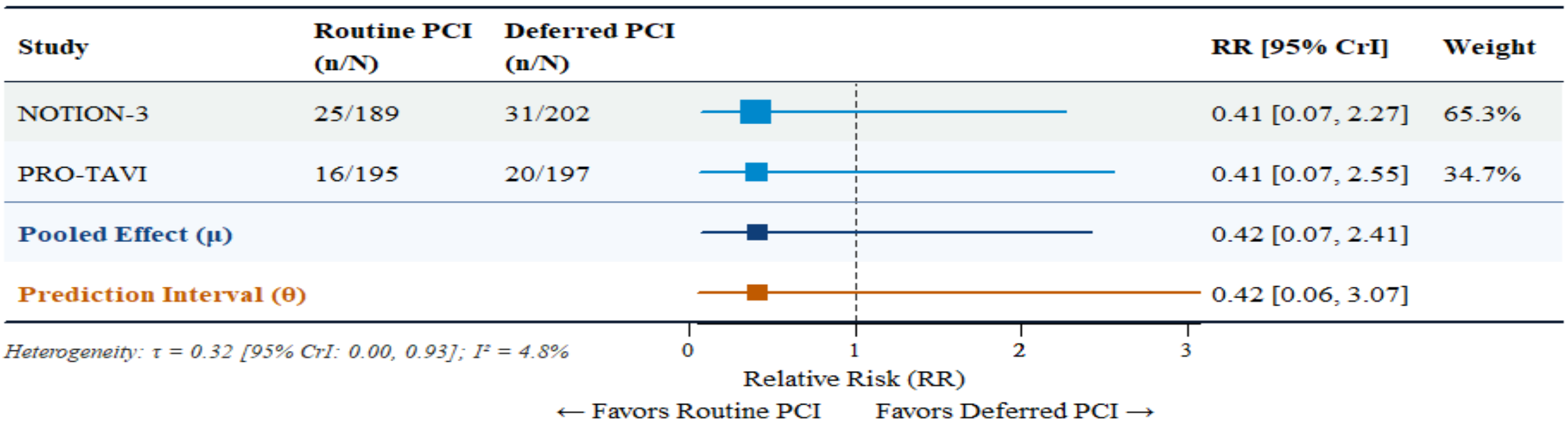

Figure S52. **Frequentist Random-Effects Meta-Analysis (REML vs. HKSJ 95% CIs): CCS Class I–II**

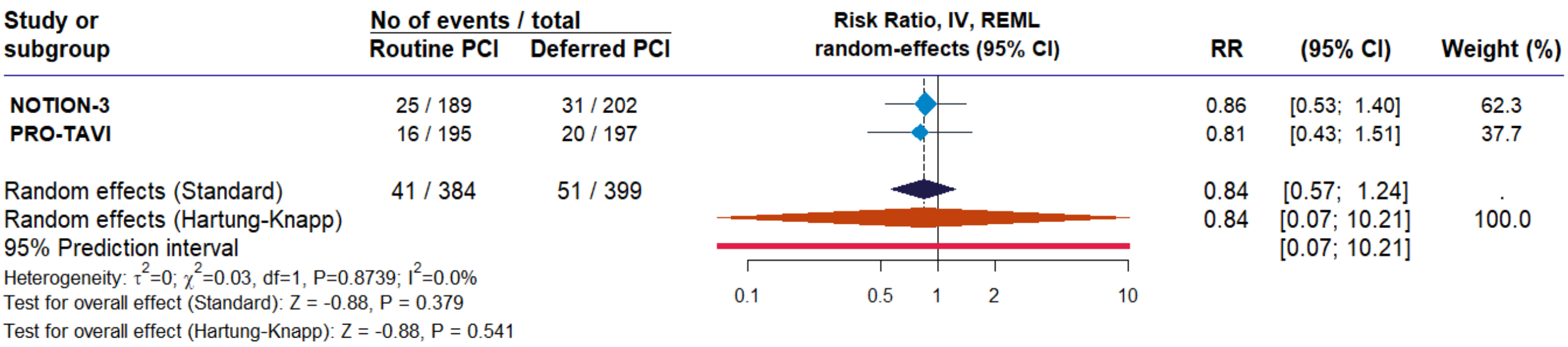

Figure S53. **Bayesian Heterogeneity Prior Sensitivity Analysis: CCS Class I–II**

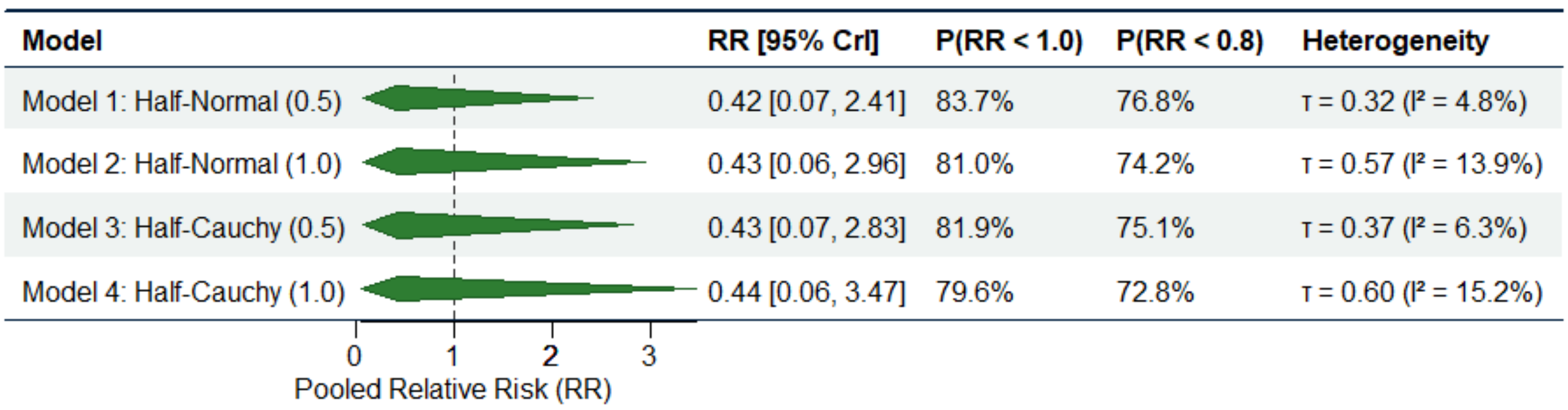

Figure S54. **Bayesian Random-Effects Meta-Analysis & Posterior Shrinkage Plot: CCS Class III**

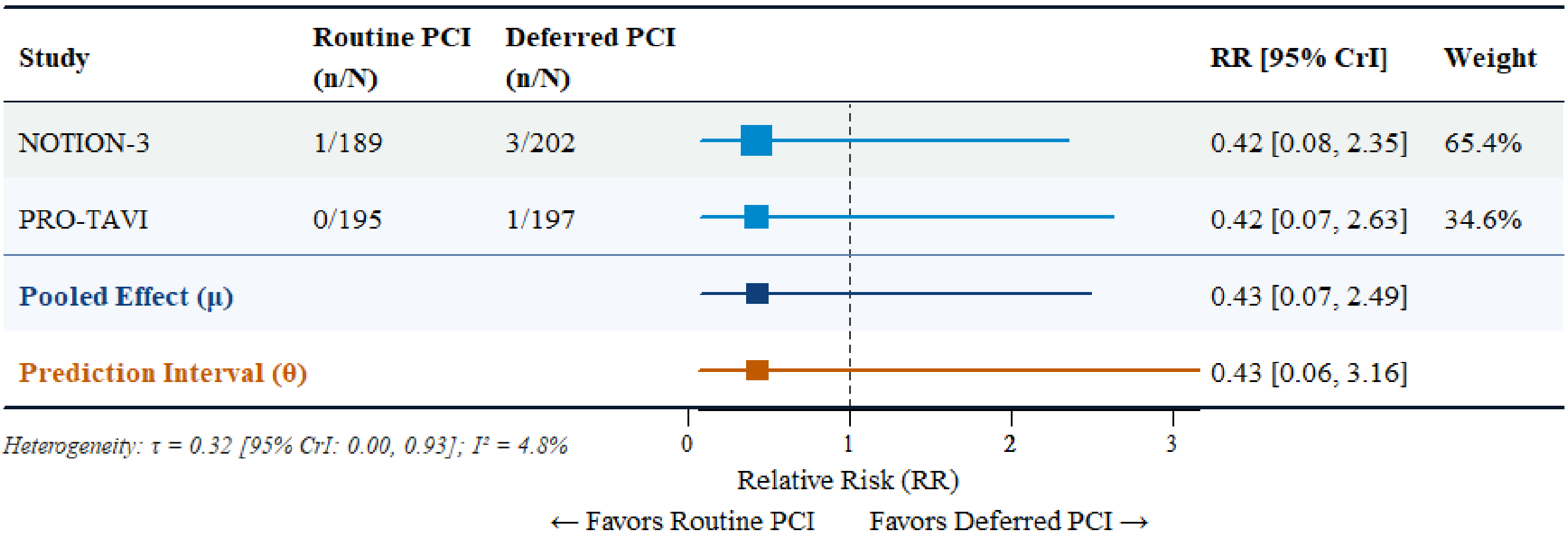

Figure S55. **Frequentist Random-Effects Meta-Analysis (REML vs. HKSJ 95% CIs): CCS Class III**

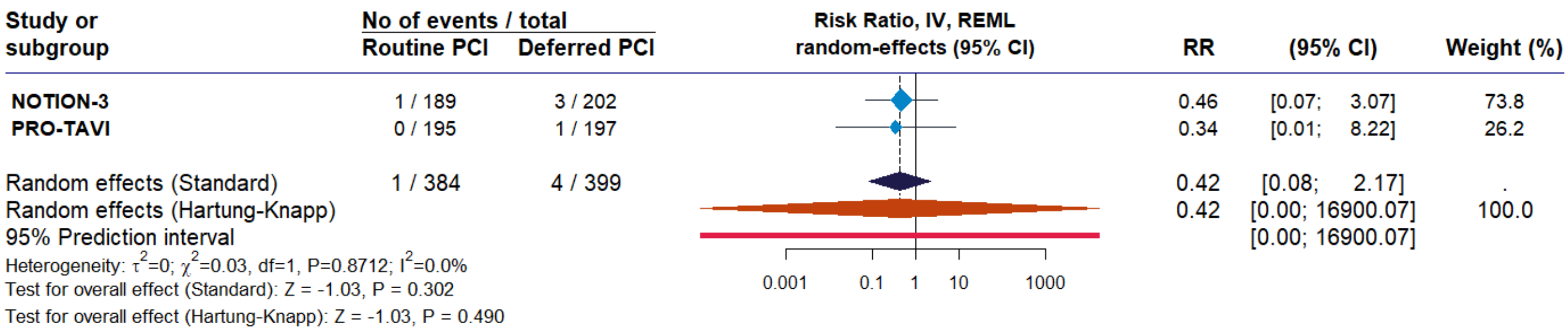

Figure S56. **Bayesian Heterogeneity Prior Sensitivity Analysis: CCS Class III**

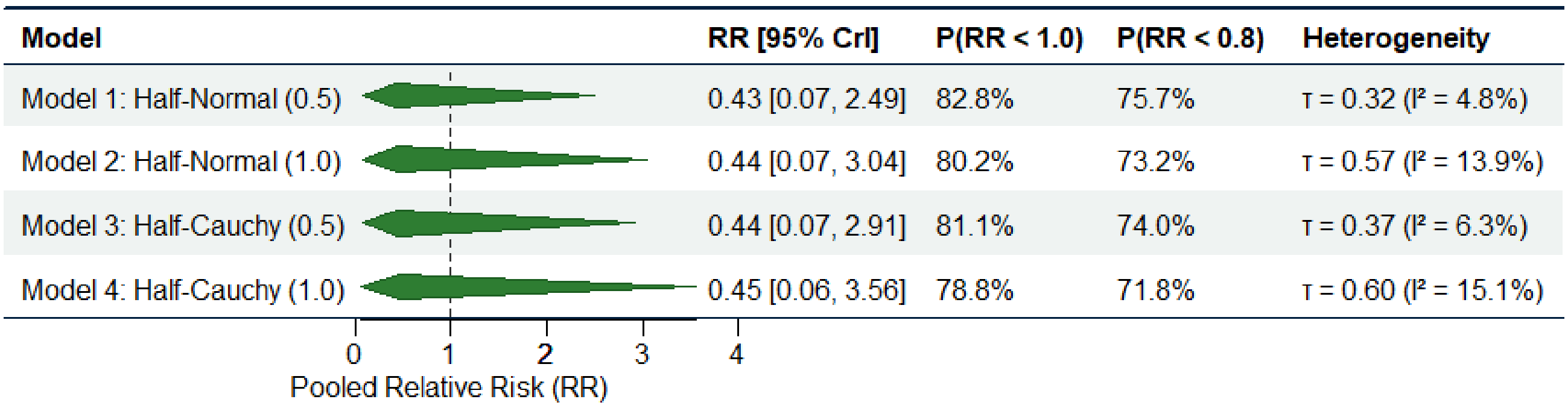

Figure S57. **Bayesian Heterogeneity Prior Sensitivity Analysis: MACE**

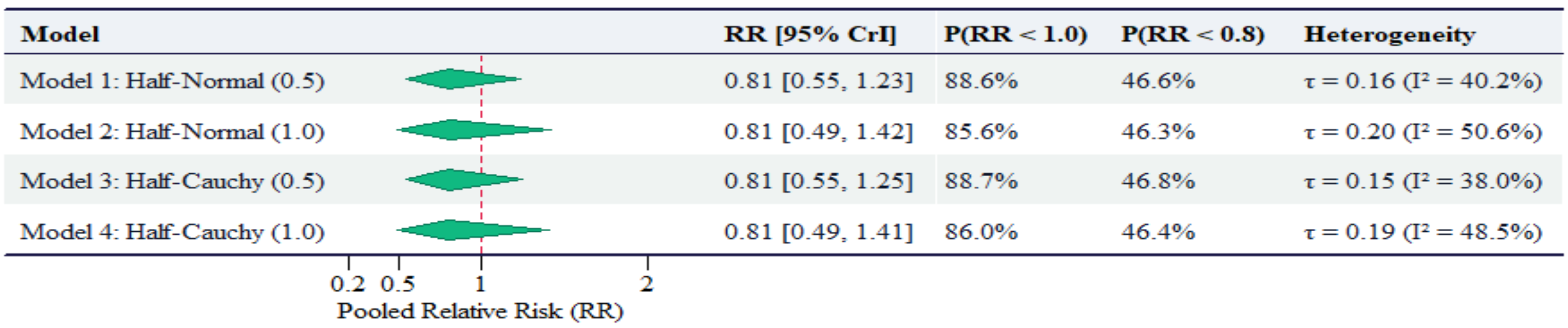

Figure S58. **Trial Sequential Analysis (TSA): Spontaneous Myocardial Infarction**

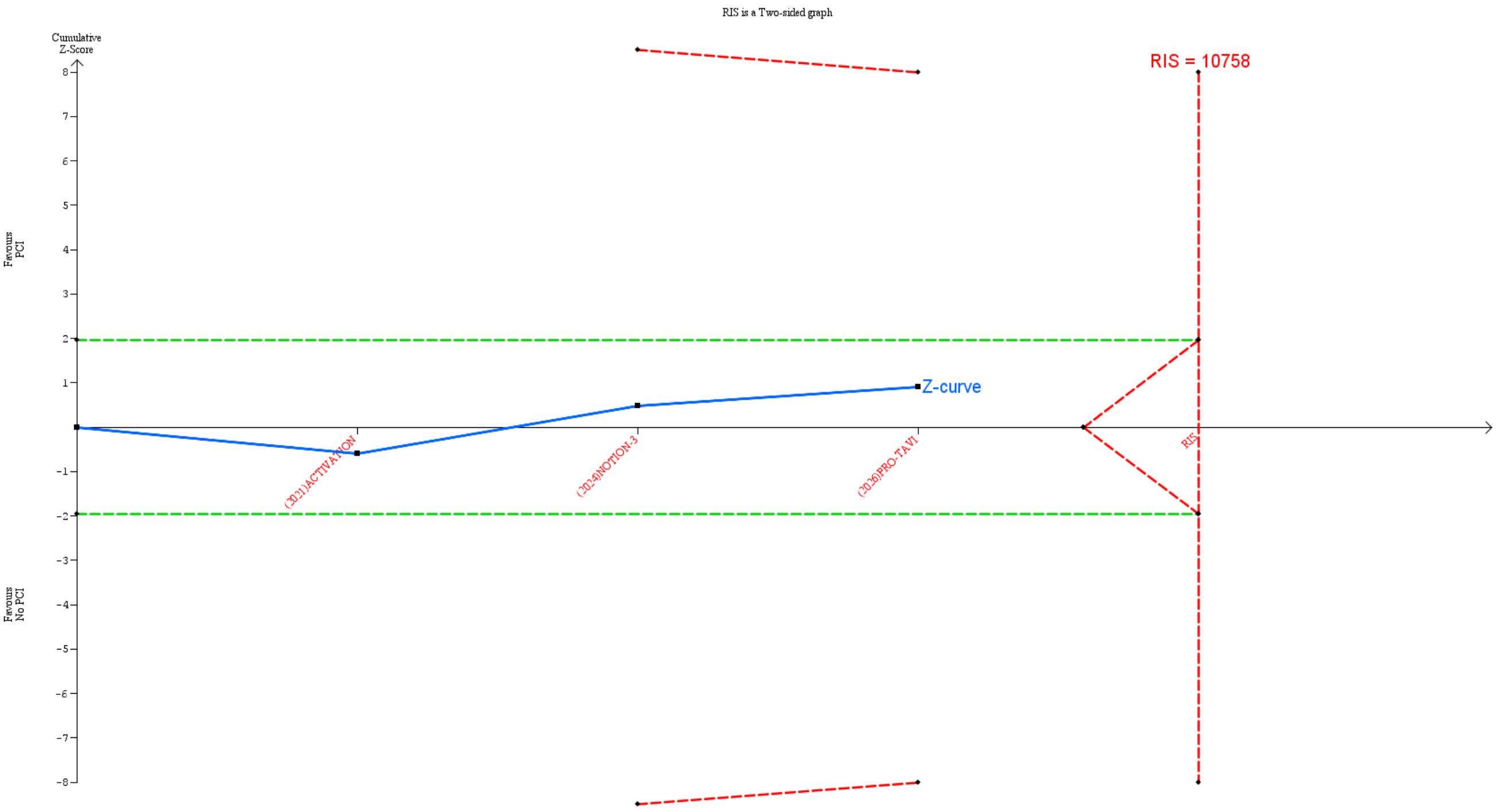

Figure S59. **Trial Sequential Analysis (TSA): Major Bleeding (Primary Safety Endpoint)**

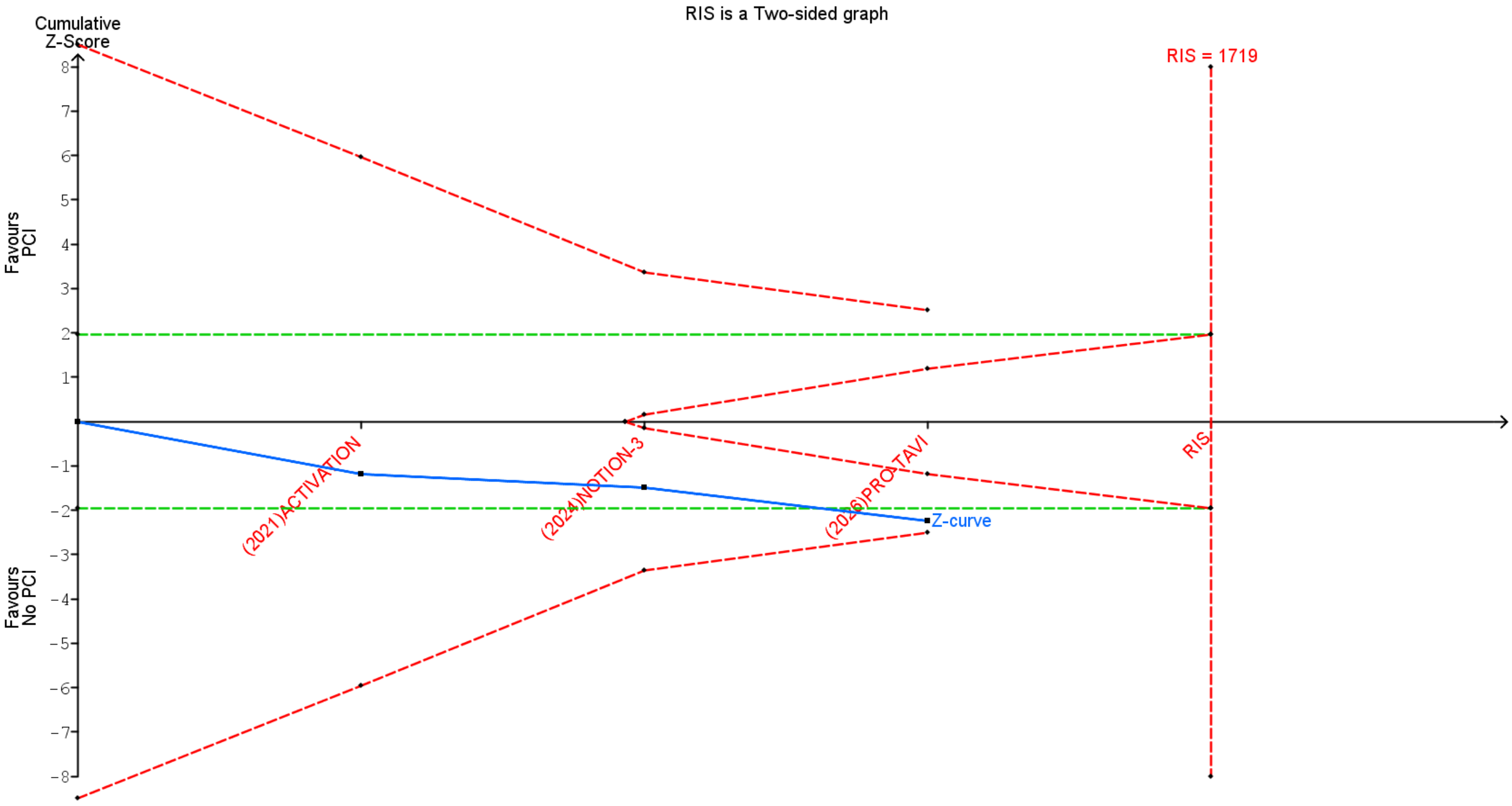

Figure S60. **Trial Sequential Analysis (TSA): All-Cause Mortality**

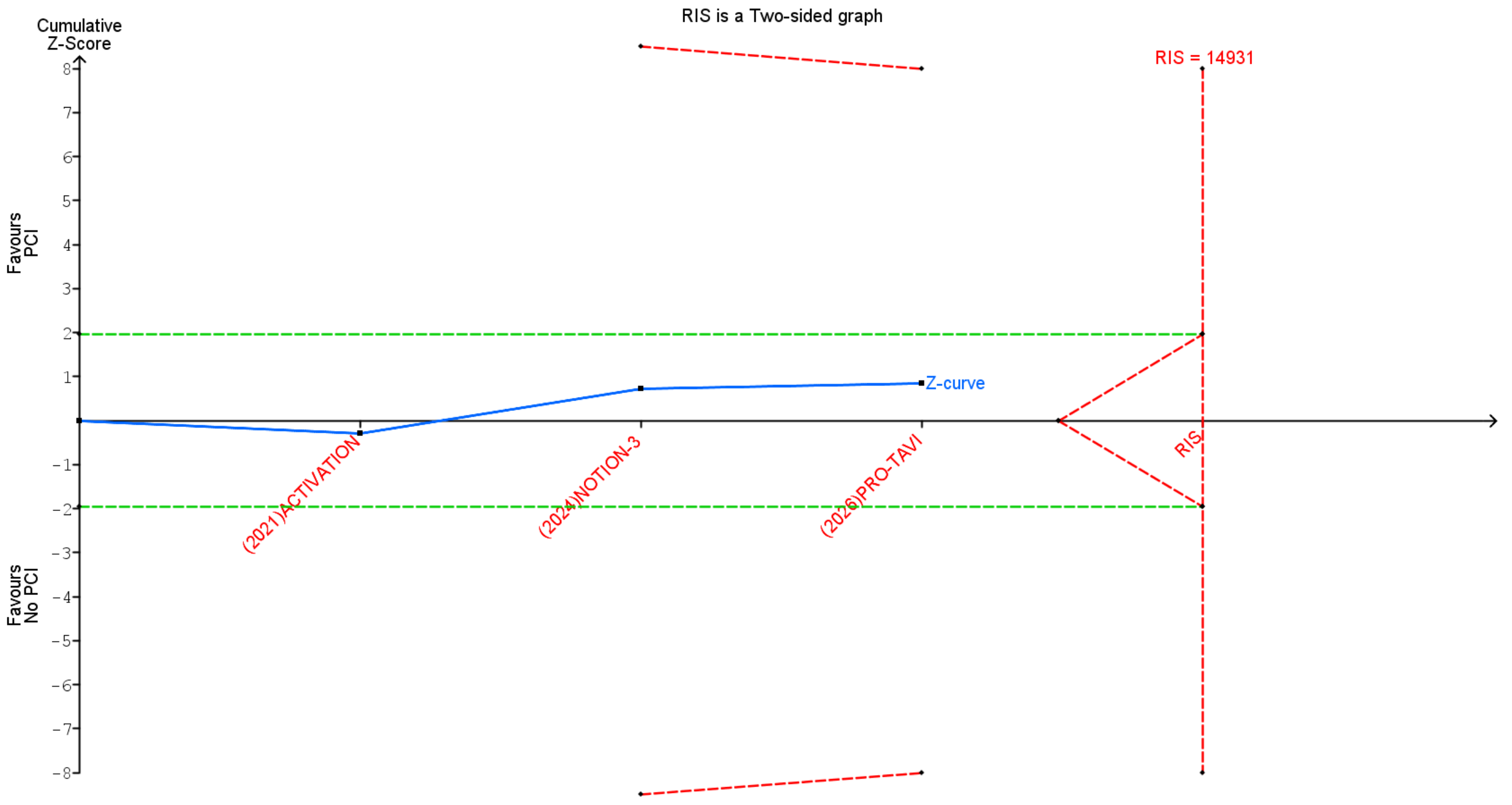

Figure S61. **Trial Sequential Analysis (TSA): Any Coronary Revascularization**

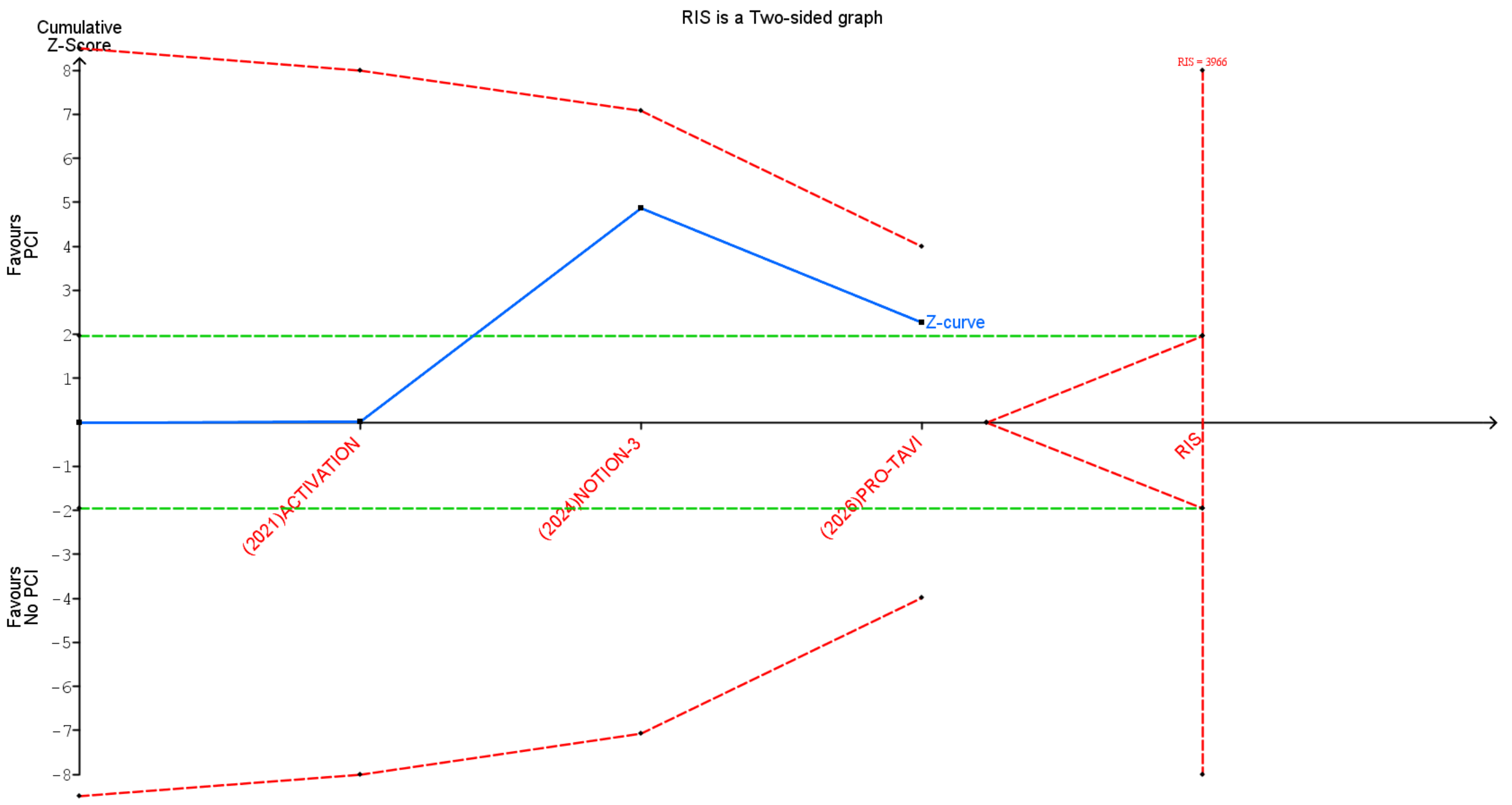

Figure S62. **Trial Sequential Analysis (TSA): Urgent Coronary Revascularization**

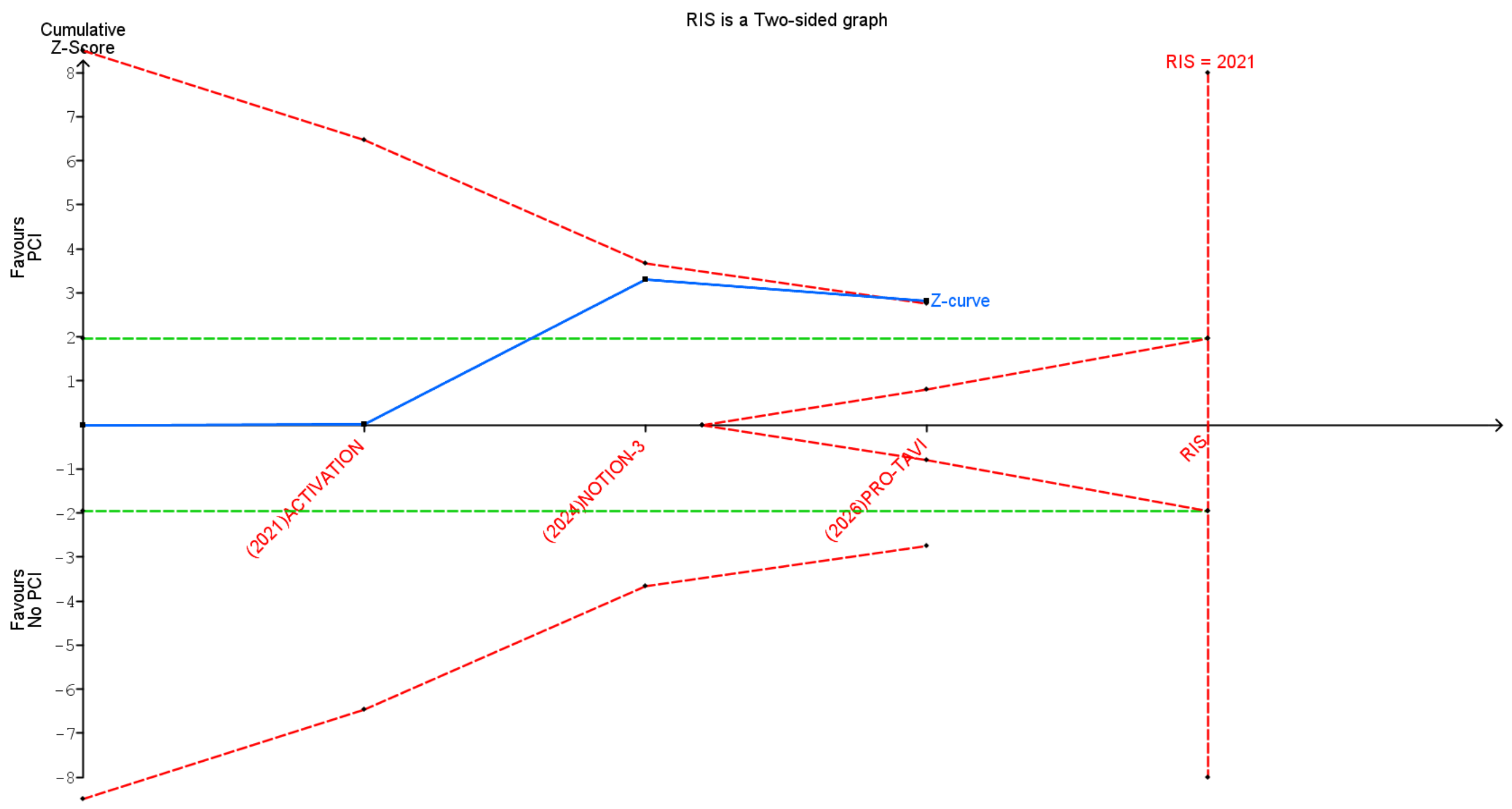

Figure S63. **Trial Sequential Analysis (TSA): Any Bleeding**

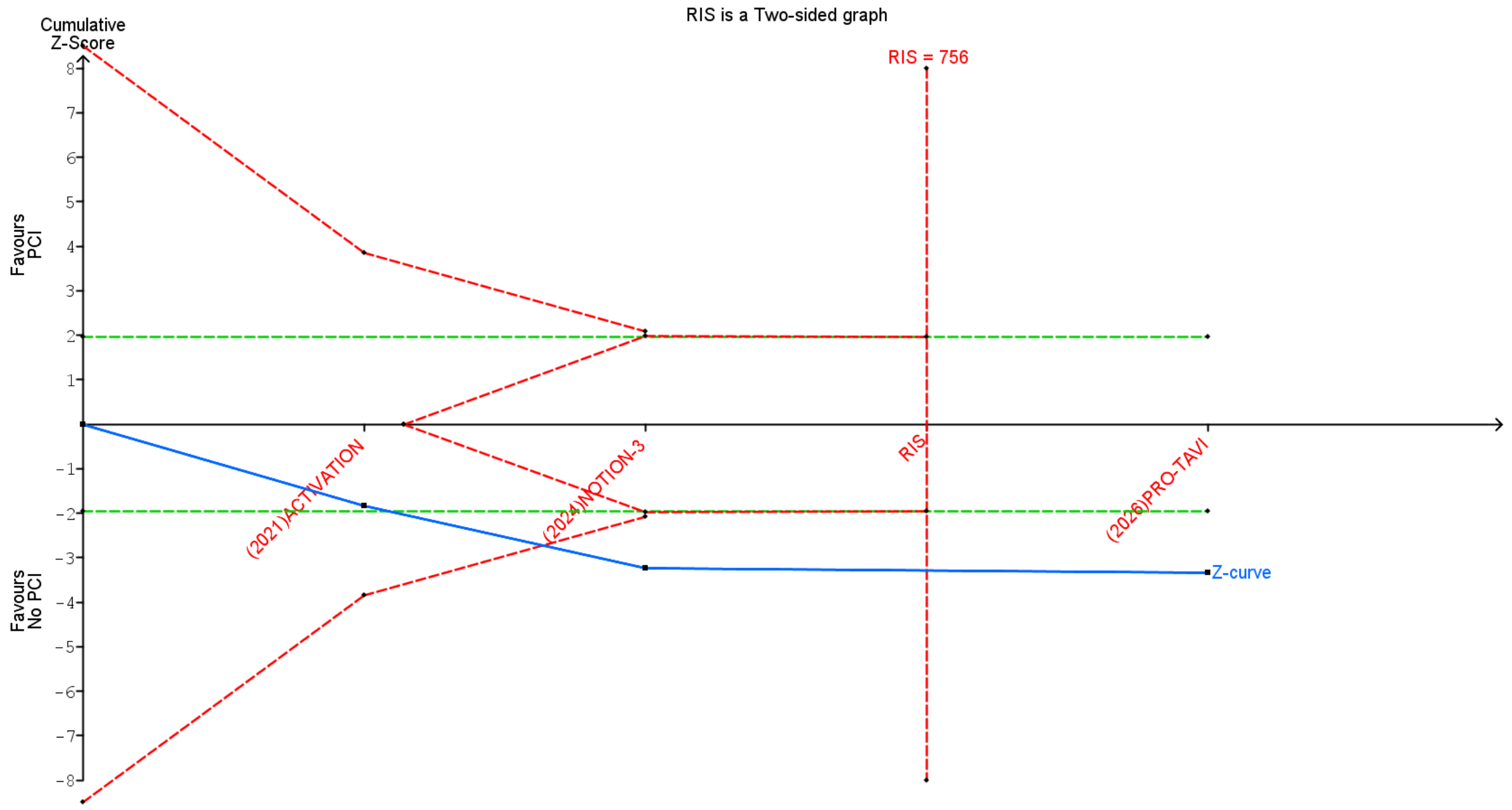

Figure S64. **Trial Sequential Analysis (TSA): Life-Threatening or Disabling Bleeding**

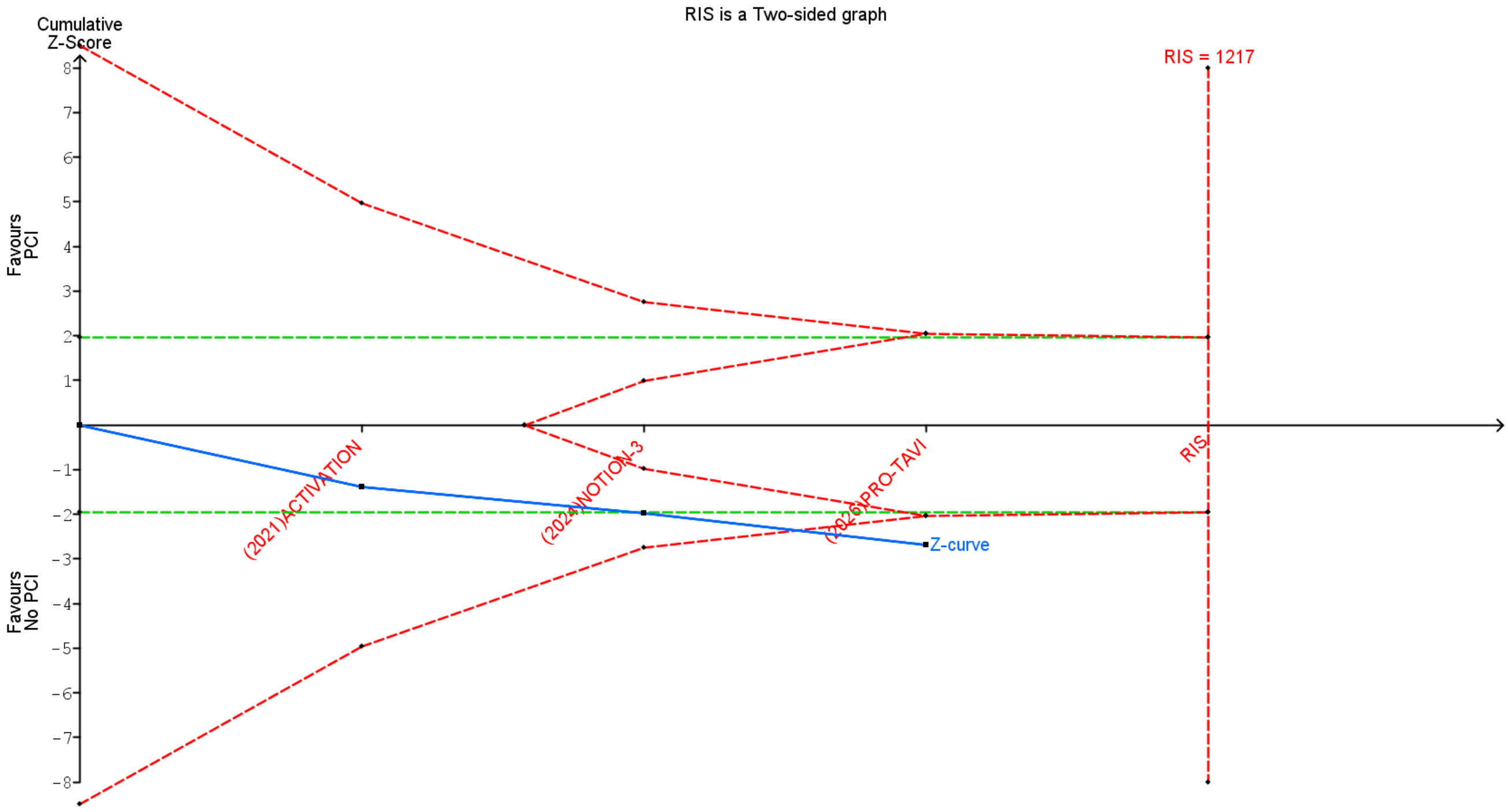

Supplement: Supplementary file 1 [file mmc1.pdf]
